# Development of 2-(4-pyridyl)-benzimidazoles as PKN2 chemical tools to probe cancer

**DOI:** 10.1016/j.bmcl.2020.127040

**Published:** 2020-04-15

**Authors:** Fiona Scott, Angela M. Fala, Lewis E. Pennicott, Tristan D. Reuillon, Katlin B. Massirer, Jonathan M. Elkins, Simon E. Ward

**Affiliations:** a Sussex Drug Discovery Centre, University of Sussex, Sussex House, Falmer, Brighton BN1 9RH, United Kingdom; b Centro de Química Medicinal (CQMED), Centro de Biologia Molecular e Engenharia Genética (CBMEG), Universidade Estadual de Campinas (UNICAMP), Campinas, SP 13083-875, Brazil; c Structural Genomics Consortium, Departamento de Genética e Evolução, Instituto de Biologia, UNICAMP, Campinas, SP 13083-886, Brazil; d Structural Genomics Consortium, Nuffield Department of Medicine, University of Oxford, Oxford OX3 7DQ, United Kingdom; e Medicines Discovery Institute, Cardiff University, Main Building, Park Place, Cardiff CF10 3AT, United Kingdom

**Keywords:** PKN, protein kinase N, Abl, Abelson murine leukemia viral oncogene, IC_50_, half maximal inhibitory concentration, AGC, protein kinase A/G/C families, PKC, protein kinase C, PRK, protein kinase C-related kinase, PAK2, p21 activated kinase 2, PRO2, glutamate 5-kinase Pro2, STK, serine/threonine kinase, PDB, protein databank, PARP, poly(ADP-ribose) polymerase, ChEMBL, European Molecular Biology Laboratory Chemical database, CLK, CDC2-like kinase, SAR, structure activity relationship, CDI, 1,1′-carbonyldiimidazole, TR-FRET, time resolved fluorescence resonance energy transfer, THF, tetrahydrofuran, EtOH, ethanol, HATU, hexafluorophosphate azabenzotriazole tetramethyl uronium, DIPEA, *N*,*N*-diisopropylethylamine, DCM, dichloromethane, AcOH, acetic acid, DMF, *N*,*N*-dimethyl-formamide, *K_D_*, dissociation constant, *K*_i_, inhibitor constant, NMR, nuclear magnetic resonance, DMSO, dimethyl sulfoxide, MeOH, methanol, GST, glutathione S-transferase, DNA, deoxyribonucleic acid, SFM, scanning force microscopy, HEPES, 4-(2-hydroxyethyl)-1-piperazineethanesulfonic acid, TCEP, *tris*(2-carboxyethyl)phosphine, EDTA, ethylenediaminetetraacetic acid, SDS, sodium dodecyl sulfate, PAGE, polyacrylamide gel electrophoresis, ATP, adenosine triphosphate, EGTA, egtazic acid, CV, column volumes, Kinases, Cancer, Heart failure, Inflammation, AGC kinase, PKN2, PRK2, Protein kinase N2, Benzimidazole, Chemical probe, Chemical tool

## Abstract

Kinases are signalling proteins which have proven to be successful targets for the treatment of a variety of diseases, predominantly in cancers. However, only a small proportion of kinases (<20%) have been investigated for their therapeutic viability, likely due to the lack of available chemical tools across the kinome. In this work we describe initial efforts in the development of a selective chemical tool for protein kinase N2 (PKN2), a relatively unexplored kinase of interest in several types of cancer. The most successful compound, **5**, has a measured IC_50_ of 0.064 μM against PKN2, with *ca.* 17-fold selectivity over close homologue, PKN1.

## Graphical abstract

**Figure:**
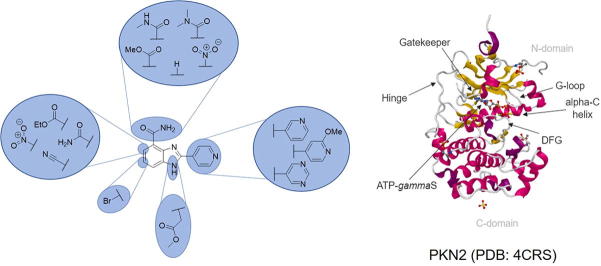

Chemical tools/probes are drug-like compounds used to answer biological questions. They need not possess all the properties of a drug candidate, which can be dialled in at a later point in the drug development process. These compounds only need to be sufficiently stable, potent and selective towards their particular target.^1^, ^2^

Historically, the approval of imatinib^3^ as an effective Abl kinase inhibitor for treating chronic myeloid leukaemia stimulated efforts to better understand the 518 human protein kinases and their role in disease. Trends in research^4^ suggest that less than 20% of the human kinome has been well-studied,^5^ and selective inhibitors are only available for an even smaller fraction of those kinases.

Protein kinase N2 (PKN2) (Fig. 1) is one of these understudied kinases. It is an AGC-type serine/threonine protein kinase. There are more than 60 AGC protein kinases in the human genome with 14 further classifications. PKN2 falls into the PKN sub-family, closely related to the PKC sub-family, and is one of three homologues (PKN1/2/3). It has a number of pseudonyms which include protein kinase C-related kinase 2 (PRK2), PKNγ, PAK2, PRO2, and STK7.^6^

**Fig. 1:**
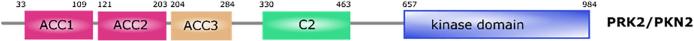
PKN2 and its domain organisation. The structure organisation contains three repeats of ACC domain (anti-parallel coiled-coil) in the *N*-termini region (pink/orange), a C2 calcium binding-like domain and in the C-terminal, the Ser/Thr kinase domain.

PKNs have a fairly conserved primary sequence and they share the same architecture. The catalytic domain of PKN2 has 87% percent identity with PKN1; 70% with PKN3; and 50% with PKC kinases, while the *N*-termini regions are less conserved, sharing only 48% and 40% between PKN1/2 and PKN2/3, respectively.^7^, ^8^

PKNs have been linked to various cellular roles, including cytoskeleton regulation,^9^ transport,^10^ cell adhesion,^11^ nutrient signalling,^12^ and cell cycle,^13^ as well as being a target of interest in colon,^14^ breast,^15^ renal,^16^ head,^17^ neck,^17^ and prostate cancers.^18^ They are also reportedly involved in inflammation^19^, ^20^ and heart failure.^21^ So far, there is one X-ray crystal structure of PKN2 publicly available in the Protein Data Bank (PDB ID: 4CRS) (Fig. 2).

**Fig. 2:**
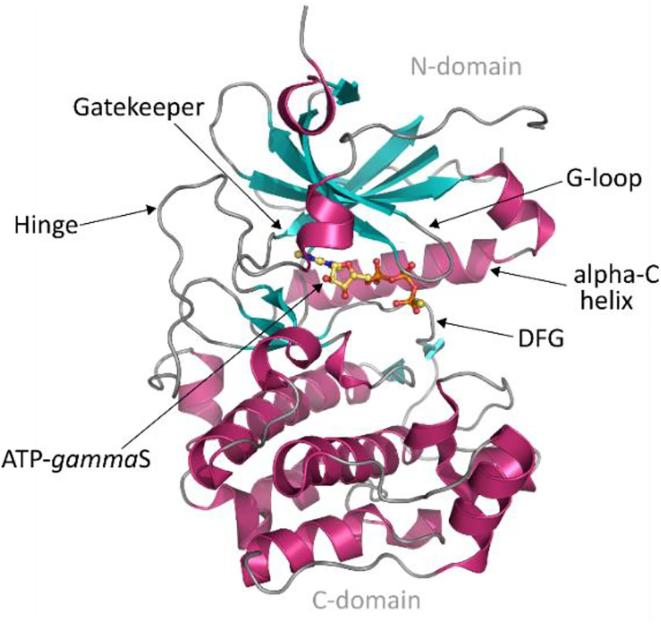
Crystallographic structure of PKN2 bound to ATP-γS (PDB ID: 4CRS).

These previous studies have elucidated functions for PKN2 using molecular and cell biology techniques, and the conclusions would be greatly supported by validation through the use of small molecule inhibitors, especially to evaluate PKN2′s potential as a cancer drug target. Potent inhibitors are known for several AGC kinase family members, including ROCK^22^, ^23^, ^24^, ^25^ and PKC,^26^ but currently there are no sufficiently selective inhibitors for PKN2.^12^

This work describes an initial effort to develop such compounds based around a benzimidazole core. Compound **5** was previously developed as a PARP inhibitor^27^, ^28^, ^29^ but exhibited higher potency towards PKN2 than its desired target. Benzimidazoles are *N*-containing heterocycles that are prevalent in medicinal chemistry.^30^ The compound was found as part of a screen of the Abbott chemical library^31^ *via* the ChEMBL database when searching for PKN2 inhibitors. It had a reported *K*_i_ of 0.040 μM against PKN2 while only inhibiting two out of 137 other kinases (PKN1 and CLK4) with potencies lower than 0.100 μM.^31^ This was deemed a good starting point for repurposing the compound as a PKN2 inhibitor. We report the synthesis of that compound and subsequent SAR studies to determine its viability as a chemical tool for establishing the potential of PKN2 as a therapeutic target.

Compound **5** was successfully synthesised *via* a four step synthesis (Scheme 1). 2-Amino-3-nitro-benzoic acid (**1**) was treated with ammonia and CDI-coupling conditions^32^ to form amide **2**. The 3-nitro group was reduced to aniline **3** with sodium dithionate,^33^ followed by the coupling of isonicotinic acid to the 3-position aniline to form amide **4**,^34^ which was then heated in acetic acid to form benzimidazole **5**.^35^

**Scheme 1:**
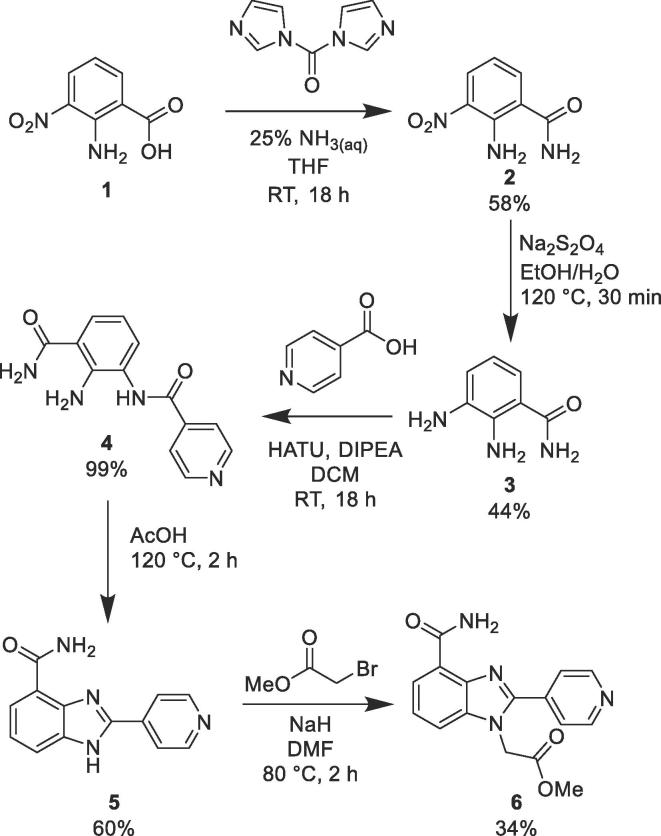
Preparation of Benzimidazoles **5** and **6**^32^, ^33^, ^34^, ^35^, ^36^

The scope of this chemistry enabled the synthesis of 14 analogues using commercially available nitroanilines and di-anilines. Additional alkylation conditions allowed the capping of the benzimidazole N-H^36^ (**6**) and alternative amide coupling conditions were used for preparing compound **11**^37^ and the penultimate amide intermediate used to make compound **19**.^38^

The potencies and selectivities of these compounds were tested using a TR-FRET binding-displacement assay in which the IC_50_ values were measured (Table 1). Calculation of *K*_i_ values using the Cheng-Prusoff equation and the *K*_D_ of the tracer (previously determined) allowed the affinity of the inhibitors for PKN2 and PKN1 to be compared (Table 1).

**Table 1:** Structure activity relationships for benzimidazoles binding to PKN2 and PKN1. Measured IC_50_ values and corresponding calculated *K*_i_ values for PKN2 and PKN1 are shown in μM. The assay Z’ factor was 0.7 < Z′ < 0.9.

**Figure:**
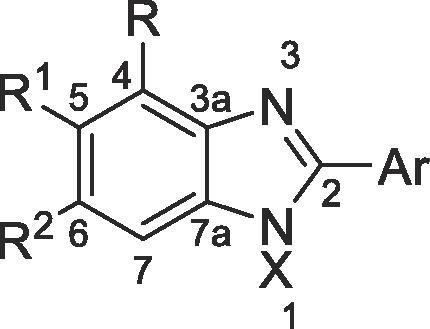

#	R	R^1^	R^2^	X	Ar	PKN2			PKN1		
						IC_50_ (µM)	Standard Deviation (µM)	*K*_i_ (µM)	IC_50_ (µM)	Standard Deviation (µM)	*K*_i_ (µM)
5	CONH_2_	H	H	H		0.064	0.001	0.03	1.10	0.11	0.54
6	CONH_2_	H	H	CH_2_COOMe		5.40	0.48	2.69	52.84	1.74	25.94
7	CONH_2_	H	H	H		16.38	3.86	8.19	66.29	38.04	32.54
8	CONH_2_	H	H	H		47.66	4.06	23.83	137.92	100.95	67.71
9	CONH_2_	H	H	H		13.79	1.18	6.90	61.59	11.95	30.23
10	CONHMe	H	H	H		2.12	0.27	1.06	10.59	1.70	5.20
11	CONMe_2_	H	H	H		38.84	9.57	19.42	56.66	25.96	27.81
12	H	CONH_2_	H	H		16.23	3.79	8.11	99.41	34.63	48.80
13	H	H	H	H		7.71	0.21	3.85	45.54	17.58	22.36
14	NO_2_	H	H	H		2.50	0.06	1.25	58.94	8.33	28.93
15	COOMe	H	H	H		31.29	5.20	15.64	38.81	13.48	19.05
16	H	COOEt	H	H		42.09	23.86	21.05	56.69	1.61	27.83
17	H	C≡N	H	H		5.02	0.79	2.51	38.54	2.26	18.92
18	H	NO_2_	H	H		25.85	0.58	12.92	109.30	33.23	53.66
19	CONH_2_	H	Br	H		0.17	0.01	0.08	4.45	0.39	2.19

Compound **5** was validated as a PKN2 inhibitor (*K*_i_ = 0.032 μM) with 17-fold selectivity over PKN1 (*K*_i_ = 0.500 μM) which was not previously included in the Abbott library screen used in the Metz et al*.* study.^31^

The benzimidazole N—H was capped using chemistry described by Tsukamoto et al*.*^36^ While the alkylation conditions given were said to be applicable to methylation of the benzimidazole using the corresponding methyl halide, this proved unsuccessful; a dimethylated product formed instead, thought to be due to the susceptibility for the 4′-pyridyl to also alkylate after the benzimidazole N—H. Repeating the specific reaction conditions used by the authors incorporated a methyl acetate ester at the 1-position (**6**) which led to loss of binding to PKN2.

Moving the 4′-pyridyl nitrogen in **7** and **8** resulted in loss of activity, as did introducing an electron-donating methoxy group at the 3′-position (**9**). This suggests the 4′-pyridyl ring acts as the hinge binder. Attempts to make the 2′-pyridyl and 4′-pyrimidine analogues were unsuccessful (Scheme 2).

**Scheme 2:**
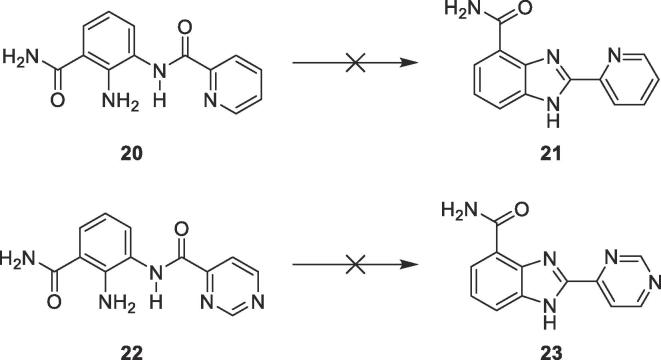
Compounds **21** and **23** could not be synthesised from intermediates **20** and **22**

Capping the amide with one (**10**) or two (**11**) methyl groups led to increasing loss of activity respectively. Potency was lost when the amide was moved to the 5-position of the benzimidazole ring (**12**), Removing the amide completely (**13**) or exchanging the 4- or 5-position for another functional group (**14**–**18**) also led to loss of activity.

Introduction of a bromine at the 6-position (**19**) was hoped to provide a useful handle for incorporating various alkyl/aryl groups at that position using Suzuki coupling chemistry.^39^, ^40^ This reaction was attempted at multiple stages of the synthetic route but was unsuccessful. Compound **19** was active against PKN2 but was nearly three times less potent than compound **5**. Despite this reduction in potency, compound **19** is 26-fold selective over PKN1.

The SAR exploration around **5** confirms that the primary amide at the 4-position, 4'-pyridyl and free N—H at the 1-position are necessary for the compound’s activity against PKN2. Subsequent analogues prepared for this series did not improve potency for the target within the PKN family but did result in a slight improvement in selectivity over PKN1 in compound **19**.

Chemical tools are needed to facilitate the exploration of lesser understood kinases such as PKN2 for its roles in healthy and cancerous cells. Benzimidazole **5** was validated as an inhibitor of PKN2 with IC_50_ 0.064 μM and with *ca.* 17-fold selectivity over PKN1 with reported high selectivity across the wider kinome^31^. Our efforts to develop a new compound to inhibit PKN2 resulted in compound **19** which was 26-fold selective for PKN2 over PKN1 despite having a near three-fold reduction in potency compared to compound **5**.
