# Supplementary material for: Development of 2-(4-pyridyl)-benzimidazoles as PKN2 chemical tools to probe cancer
Source: Bioorg Med Chem Lett. 2020 Apr 15;30(8):127040. doi: 10.1016/j.bmcl.2020.127040 (PMC7078758; doi:10.1016/j.bmcl.2020.127040)
Supplement: Supplementary data 1 [file mmc1.docx]

**Supplementary Information – Experimental Methods**

**Chemistry**

All commercial reagents were purchased from Sigma-Aldrich, Alfa Aesar, Apollo Scientific, Fisher Scientific, Fluorochem, Tokyo Chemical Industry or Manchester Organics and were of the highest available purity. Unless otherwise stated, chemicals were used as supplied without further purification. Anhydrous solvents were purchased from Acros (AcroSeal™) or Sigma-Aldrich (SureSeal™) and were stored under nitrogen. Anhydrous solvents and reagents were used as purchased. Petroleum ether refers to the fraction with a boiling point between 40 °C and 60 °C.

Reactions were magnetically stirred and monitored by liquid chromatography mass spectrometry (LCMS) or thin layer chromatography (TLC) using aluminium-supported thin layer chromatography sheets with Merck silica gel 60 F254. For TLC, the eluent was as stated (where this consisted of more than one solvent, the ratio is stated as volume: volume). Visualisation was by absorption of UV light (ν_max_ 254 or 365 nm).

Flash column chromatography was carried out using either: commercial pre-packed silica columns from Biotage (SNAP and Zip), Isco (RediSep), Grace (Reveleris) or filled with Merck silica gel 60 (40-63 μm); C18 silica (Biotage SNAP KP-C18-HS, Grace Reveleris C18); amino silica (Biotage SNAP KP-NH or Grace Reveleris Amino) on an ISCO Combiflash Rf or a Biotage Isolera Prime.

**^1^H and ^13^C NMR** spectra were recorded at 500 or 600 MHz on a Varian VNMRS 500/600 MHz spectrometer (at 30 °C), using residual isotopic solvent as an internal reference. The chemical shift data for each signal are given as δ_H_ in units of parts per million (ppm). Each spectrum is corrected to the appropriate solvent reference; δ (CHCl_3_) = 7.27/77 ppm or δ (DMSO) = 2.50/39.52 ppm for ^1^H/^13^C NMR respectively. The multiplicity of each δ_H_ signal is indicated by: s (singlet); br s (broad singlet); d (doublet); t (triplet); q (quartet); or m (multiplet). The number of protons (n) for a given resonance signal is indicated by nH. Coupling constants (*J*) are quoted in Hz and are recorded to the nearest 0.1 Hz. Identical proton coupling constants (*J*) are averaged in each spectrum and reported to the nearest 0.1 Hz. The coupling constants are determined by analysis using MestReNova version 10 software.

**LCMS (LCQ)** data was recorded on a Waters 2695 HPLC using a Waters 2487 UV detector and a Thermo LCQ ESI-MS. Samples were eluted through a Phenomenex Luna 3 μ C18 50 mm × 4.6 mm column, using water and acetonitrile acidified by 0.1% formic acid at 1.5 mL/min and detected at 254 nm.

**LCMS (MDAP)** data was recorded on a Shimadzu Prominence Series coupled to a LCMS-2020 ESI and EI mass spectrometer. Samples were eluted through a Phenomenex Gemini 5 μ C18 110 Å 250 mm × 4.6 mm column, using water and acetonitrile acidified by 0.1% formic acid at 1 mL/min and detected at 254 nm.

**High resolution mass spectrometry:** HRMS data (ESI)^+^ was recorded on Bruker Daltonics, Apex III, ESI source: Apollo ESI with methanol as spray solvent. Only molecular ions, fractions from molecular ions and other major peaks are reported as mass/charge (*m/z*) ratios.

**General Method A (CDI amide coupling)**^1^

1,1'-Carbonyldiimidazole (CDI) (1 eq.) was added portion-wise to the corresponding benzoic acid (1 eq.) in THF (20 mL/g) at room temperature. The reaction mixture was heated to reflux for 30 min to facilitate the coupling of the CDI to the acid, allowed to cool and then the corresponding amine (1.1 eq.) was added in one portion. The reaction was stirred at room temperature overnight resulting in the formation of an orange suspension. The product was isolated as per specification.

**General Method B (nitro reduction)**^2^

The corresponding nitroaniline (1 eq.) was added to 50% aqueous ethanol (5 mL/g) at room temperature and heated to reflux. Sodium dithionite (10 eq.) was added portion-wise. The reaction was heated under reflux for 30 min. TLC confirmed consumption of starting material (100% ethyl acetate, UV). The cooled reaction mixture was extracted four times with ethyl acetate (4 × 10 mL/g) and the combined organic phases were dried over MgSO_4_, filtered and concentrated under reduced pressure. The product was isolated as per specification.

**General Method C (HATU amide coupling)**^3^

The corresponding dianiline (1 eq.), the corresponding aromatic acid (1.1 eq.), HATU (1.1 eq.) and *N*,*N*-diisopropyl-ethylamine (2 eq.) in dichloromethane (2 mL/g) was stirred overnight at room temperature. The product was isolated as per specification.

**General Method D (acid-catalysed cyclisation)**^4^

The corresponding amide (1 eq.) was dissolved in acetic acid (excess *ca.* 140 eq.) and heated to 120 °C. The reaction was monitored by TLC and after 1.5 h, all of the starting material had been consumed. The product was isolated as per specification.

**2-Amino-3-nitro-benzamide (2)**^5–7^

Compound **2** was prepared *via* General Method A using 2-amino-3-nitro-benzoic acid (10 g) and 13.4 M aqueous ammonia solution (1.1 eq.). The reaction mixture was filtered giving 2-amino-3-nitro-benzamide (**2**) (2.24 g, 21%) as a fine orange solid. The filtrate was partially concentrated under reduced pressure, left overnight and a further crop of 2-amino-3-nitro-benzamide (**2**) (3.91 g, 37% yield) was isolated by filtration as a fine orange solid.

1H NMR (500 MHz, DMSO-*d*_6_) δ 8.47 (s, 2H, H-9), 8.25–8.07 (m, 2H, H-4 and H-10), 8.01–7.86 (m, 1H, H-6), 7.62 (s, 1H, H-10), 6.67 (t, *J* = 7.4Hz, 1H, H-5); LCMS (MDAP) Rt = 24.9 min (Ana 9–95); HRMS *m/z* (ESI)^+^ 204.0382 (M+Na)^+^, calculated MW for C_7_H_7_N_3_O_3_Na 204.1390.

**2,3-Diaminobenzamide (3)**^8–10^

Compound **3** was prepared *via* General Method B using 2-amino-3-nitro-benzamide (**2**) (6 g). The crude was purified *via* flash column chromatography (100 g silica, ethyl acetate:methanol, 100:0 to 90:10) to give 2,3-diaminobenzamide (**3**) (2.21 g, 44%) as a light brown solid.

^1^H NMR (500 MHz, DMSO-*d*_6_) δ 7.63 (s, 1H, H-10), 7.00 (s, 1H, H-10), 6.90 (d, *J* = 8.0 Hz, 1H, H-6), 6.62 (d, *J* = 7.5 Hz, 1H, H-4), 6.35 (t, *J* = 7.7 Hz, 1H, H-5), 6.08 (s, 2H, H-9), 4.63 (s, 2H, H-11); LCMS (MDAP) Rt = 19.3 min (Ana 9–95); HRMS *m/z* (ESI)^+^ 174.0638 (M+Na)^+^, calculated MW for C_7_H_9_N_3_ONa 174.1560.

***N*-(2-Amino-3-carbamoyl-phenyl)pyridine-4-carboxamide** (**4**)

Compound **4** was prepared *via* General Method C using 2,3-diaminobenzamide (**3**) (150 mg, 1 eq.) and isonicotinic acid (1.1 eq.). The reaction mixture was filtered giving *N*-(2-amino-3-carbamoyl-phenyl)-pyridine-4-carboxamide (**4**) (245 mg, 96%) as an off-white solid.

^1^H NMR (500 MHz, DMSO-*d*_6_) δ 9.91 (s, 1H, H-11), 8.77 (d, *J* = 5.3 Hz, 2H, H-2'and H-6'), 7.91 (d, *J* = 5.5 Hz, 2H, H-3'and H-5'), 7.87 (s, 1H, H-10), 7.53 (d, *J* = 7.9 Hz, 1H, H-6), 7.26 (d, *J* = 7.6 Hz, 1H, H-4), 7.23 (s, 1H, H-10), 6.57 (t, *J* = 7.8 Hz, 1H, H-5), 6.52 (s, 2H, H-9); LCMS (LCQ) Rt = 0.8 min (4 min method); HRMS *m/z* (ESI)^+^ 256.9010 (M+H)^+^, calculated MW for C_13_H_12_N_4_O_2_ 256.2595.

**2-(4-Pyridyl)-1*H*-benzimidazole-4-carboxamide (5)**^11–15^

Compound **5** was prepared *via* General Method D using *N*-(2-amino-3-carbamoyl-phenyl)pyridine-4-carboxamide (**4**) (330 mg, 1 eq.). The reaction mixture was concentrated under reduced pressure to give a pale yellow crude solid (37 mg). The crude was purified *via* flash column chromatography (11 g amino silica, ethyl acetate:methanol, 100:0 to 85:15) to give 2-(4-pyridyl)-1*H*-benzimidazole-4-carboxamide (**5**) (193 mg, 60%) as a yellow solid.

^1^H NMR (500 MHz, DMSO-*d*_6_) δ 9.19 (s, 2H, H-10), 8.80 (d, *J* = 4.3 Hz, 2H, H-2'and H-6'), 8.19 (d, *J* = 4.3 Hz, 2H, H-3' and H-5'), 7.91 (d, *J* = 7.3 Hz, 1H, H-7), 7.84 (s, 1H, H-1), 7.81 (d, *J* = 7.9 Hz, 1H, H-5), 7.41 (t, *J* = 7.7 Hz, 1H, H-6); ^13^C NMR (126 MHz, DMSO-*d*_6_) δ 170.76 (C-7), 166.49 (C-3a), 151.04 (C-2' and C-6'), 149.99 (C-4'), 136.70 (C-2), 123.90 (C-7), 123.58 (C-5), 123.15 (C-6), 121.17 (C3' and C-5'), 116.42 (C-7a); LCMS (LCQ) Rt = 0.6 min (4 min method); HRMS *m/z* (ESI)^+^ 239.0920 (M+H)^+^, calculated MW for C_13_H_10_N_4_O 238.2443.

**Methyl 2-[4-carbamoyl-2-(4-pyridyl)benzimidazol-1-yl]acetate (6)**^16^

To a solution of 2-(4-pyridyl)-1*H*-benzimidazole-4-carboxamide (**5**) (50 mg, 1 eq.) in *N*,*N*-dimethylformamide (1 mL) was added methyl bromoacetate (0.03 mL, 1.5 eq.) and sodium hydride (10 mg, 2 eq.). The reaction mixture was stirred at 80 °C for 2 h. The reaction mixture was concentrated under reduced pressure and chloroform (5 mL) was added. The mixture was extracted five times with water (5 × 2 mL). The organic phase was dried over MgSO_4_, filtered and concentrated under reduced pressure before being recrystallised from cyclohexane to give methyl 2-[4-carbamoyl-2-(4-pyridyl)-benzimidazol-1-yl]acetate (**6**) (22 mg, 34%) as an orange solid.

^1^H NMR (600 MHz, DMSO-*d*_6_) δ 9.03 (s, 1H, H-16), 8.81 (d, *J* = 5.5 Hz, 2H, H-2' and H-6'), 7.98 (d, *J* = 7.5 Hz, 1H, H-5), 7.92 (d, *J* = 8.1 Hz, 1H, H-7), 7.85 (s, 1H, H-16), 7.80 (d, *J* = 5.6 Hz, 2H, H-3' and H-5'), 7.48 (t, *J* = 7.8 Hz, 1H, H-6), 5.45 (s, 2H, H-8), 3.67 (s, 3H, H-12); LCMS (MDAP) Rt = 0.7 min (Ana 5–95 in 5 minutes); HRMS *m/z* (ESI)^+^ 311.1011 (M+H)^+^, calculated MW for C_16_H_14_N_4_O_3_ 310.3068.

***N*-(2-Amino-3-carbamoyl-phenyl)pyridine-3-carboxamide**

The intermediate was prepared *via* General Method C using 2,3-diaminobenzamide (**3**) (150 mg, 1 eq.) and nicotinic acid (1.1 eq.). *N*-(2-Amino-3-carbamoyl-phenyl) pyridine-3-carboxamide (229 mg, 85%) was isolated by filtration as a light brown solid.

^1^H NMR (500 MHz, DMSO-*d*_6_) δ 9.84 (s, 1H, H-11), 9.16 (s, 1H, H-6’), 8.78 – 8.70 (m, 1H, H-2’), 8.33 (d, *J* = 7.8 Hz, 1H, H-4’), 7.88 (s, 1H, H-10), 7.60 – 7.47 (m, 2H, H-6 and H-3’), 7.27 (d, *J* = 7.4 Hz, 1H, H-4), 7.23 (s, 1H, H-10), 6.56 (m, 3H, H-5 and 2 × H-9); LCMS (LCQ) Rt = 0.6 min (4 min method); HRMS *m/z* (ESI)^+^ 257.1032 (M+H)^+^, calculated MW for C_13_H_12_N_4_O_2_ 256.2595.

**2-(3-Pyridyl)-1*H*-benzimidazole-4-carboxamide (7)**^14,15,17^

Compound **7** was prepared *via* General Method D using *N*-(2-Amino-3-carbamoyl-phenyl)pyridine-3-carboxamide (179 mg, 1 eq.). The reaction mixture was concentrated under reduced pressure to give a pale-yellow crude solid (37 mg). The crude was purified *via* flash column chromatography (11 g amino silica, ethyl acetate:methanol, 100:0 to 90:10) to give 2-(3-pyridyl)-1*H*-benzimidazole-4-carboxamide (**7**) (45 mg, 26%) as an off-white solid.

^1^H NMR (500 MHz, DMSO-*d*_6_) δ 13.62 (s, 1H, H-1), 9.42 (s, 1H, H-6’), 9.29 (s, 1H, H-10), 8.72 (d, *J* = 7.1 Hz, 1H, H-7), 8.59 (d, *J* = 7.1 Hz, 1H, H-5), 7.94 – 7.87 (d, *J* = 7.90 Hz, 1H, H-2’), 7.83 (s, 1H, H-10), 7.78 (d, *J* = 7.5 Hz, 1H, H-4’), 7.62 (t, *J* = 7.5 Hz, 1H, H-6), 7.38 (t, *J* = 7.0 Hz, 1H, H-5’); LCMS (LCQ) Rt = 0.6 min (4 min method); HRMS *m/z* (ESI)^+^ 261.0741 (M+Na)^+^, calculated MW for C_13_H_10_N_4_ONa 261.2351.

***N*-(2-Amino-3-carbamoyl-phenyl)-pyrimidine-5-carboxamide**

The intermediate was prepared *via* General Method C using 2,3-diaminobenzamide (150 mg, 1 eq.) and pyrimidine-5-carboxylic acid (1.1 eq.). *N*-(2-amino-3-carbamoyl-phenyl)-pyrimi-dine-5-carboxamide (240 mg, 94%) was isolated by filtration as a pale brown solid. The crude was taken on without further purification.

^1^H NMR (500 MHz, DMSO-*d*_6_) δ 10.09 (s, 1H, H-11), 9.34 (s, 1H, H-2'), 9.32 (s, 2H, H-4' and H-6'), 7.89 (s, 1H, H-10), 7.54 (d, *J* = 7.4 Hz, 1H, H-6), 7.28 (d, *J* = 7.1 Hz, 1H, H-4), 7.23 (s, 1H, H-10), 6.64 (s, 2H, H-9), 6.57 (t, *J* = 7.4 Hz, 1H, H-5); LCMS (LCQ) Rt = 0.7 min (4 min method); HRMS *m/z* (ESI)^+^ 280.0813 (M+Na)^+^, calculated MW for C_12_H_11_N_5_O_2_Na 280.2385.

**2-Pyrimidin-5-yl-1*H*-benzimidazole-4-carboxamide (8)**

Compound **8** was prepared *via* General Method D using *N*-(2-amino-3-carbamoyl-phenyl)-pyri-midine-5-carboxamide (140 mg, 1 eq.). The reaction mixture was concentrated under reduced pressure and was purified *via* flash column chromatography (11 g amino silica, ethyl acetate:methanol 100:0 to 85:15) to give 2-pyrimidin-5-yl-1*H*-benzimidazole-4-carboxamide (**7**) (90 mg, 66%) as a yellow solid.

^1^H NMR (500 MHz, DMSO-*d*_6_) δ 9.57 (s, 2H, H-4' and H-6'), 9.30 (s, 1H, H-2'), 9.09 (s, 2H, H-10), 7.90 (d, *J* = 7.3 Hz, 1H, H-5), 7.84 – 7.72 (m, 2H, H-1 amd H-7), 7.37 (t, *J* = 7.7 Hz, 1H, H-6); LCMS (LCQ) Rt = 0.6 min (4 min method); HRMS *m/z* (ESI)^+^ 262.0705 (M+Na)^+^, calculated MW for C_12_H_9_N_5_ONa 262.2232.

***N*-(2-Amino-3-carbamoyl-phenyl)-2-methoxy-pyridine-4-carboxamide**

The intermediate was prepared *via* General Method C using 2,3-diaminobenzamide (150 mg, 1 eq.) and 2-methoxyisonicotinic acid (1.1 eq.). *N*-(2-Amino-3-carbamoyl-phenyl)-2-methoxy-pyridine-4-carboxamide (242 mg, 81%) was isolated by filtration as a pale brown solid.

^1^H NMR (500 MHz, DMSO-*d*_6_) δ 9.84 (s, 1H, H-11), 8.33 (d, *J* = 5.3 Hz, 1H, H-6'), 7.87 (s, 1H, H-10), 7.53 (d, *J* = 8.0 Hz, 1H, H-4), 7.48 (d, *J* = 5.3 Hz, 1H, H-5), 7.37 (s, 1H, H-3'), 7.27 – 7.18 (m, 2H, H-10 and H-6), 6.57 (t, *J* = 7.8 Hz, 1H, H-5), 6.51 (s, 2H, H-9), 3.91 (s, 3H, H-8'); LCMS (LCQ) Rt = 0.6 min (4 min method); HRMS *m/z* (ESI)^+^ 309.0963 (M+Na)^+^, calculated MW for C_14_H_14_N_4_O_3_Na 309.2764.

**2-(2-Methoxy-4-pyridyl)-1*H*-benzimidazole-4-carboxamide (9)**

Compound **9** was prepared *via* General Method D using *N*-(2-amino-3-carbamoyl-phenyl)-2-methoxy-pyridine-4-carboxamide (190 mg, 1 eq.). The reaction mixture was concentrated under reduced pressure. The crude was purified *via* flash column chromatography (11 g amino silica, ethyl acetate:methanol, 100:0 to 90:10) to give 2-(2-methoxy-4-pyridyl)-1*H*-benzimidazole-4-carboxamide (**9**) (152 mg, 81%) as a pale yellow solid.

^1^H NMR (500 MHz, DMSO-*d*_6_) δ 13.64 (s, 1H, H-1), 9.22 (s, 1H, H-10), 8.35 (d, *J* = 4.7 Hz, 1H, H-6'), 7.91 (d, *J* = 6.7 Hz, 1H, H-5), 7.83 (s, 1H, H-1), 7.82 – 7.76 (m, 2H, H-7 and H-5'), 7.61 (s, 1H, H-3'), 7.40 (t, *J* = 7.3 Hz, 1H, H-6), 3.92 (s, 3H, H-8'); LCMS (LCQ) Rt = 0.6 min (4 min method); HRMS *m/z* (ESI)^+^ 291.0857 (M+Na)^+^, calculated MW for C_14_H_12_N_4_O_2_Na 291.2611.

**2-Amino-*N*-methyl-3-nitro-benzamide**

The intermediate was prepared *via* General Method A using 2-amino-3-nitro-benzoic acid (2 g, 1 eq.) and 13.4 M aqueous methylamine (1.1 eq.). The reaction mixture was concentrated under reduced pressure. The crude was purified *via* flash column chromatography (30 g silica, petroleum ether:ethyl acetate, 100:0 to 0:100) to give a 1:2 mixture of 2-amino-*N*-methyl-3-nitro-benzamide (2.00 g, 32%) and imidazole. The product was carried forward without further purification.

^1^H NMR (500 MHz, CDCl_3_) δ 8.25 (d, *J* = 8.5 Hz, 1H, H-4), 8.21 (s, 2H, H-11), 7.67 (d, 1H, H-6), 6.60 (t, *J* = 8.0 Hz, 1H, H-5), 2.99 (d, *J* = 4.7 Hz, 3H, H-10); LCMS (LCQ) Rt = 0.8 min (4 min method); HRMS *m/z* (ESI)^+^ 195.9200 (M+H)^+^, calculated MW for C_8_H_9_N_3_O_3_ 195.1750.

**2,3-Diamino-*N*-methyl-benzamide**

The intermediate was prepared *via* General Method B using 2-amino-*N*-methyl-3-nitro-benzamide (1 g, 1 eq.). The crude was purified *via* flash column chromatography (10 g silica, ethyl acetate:methanol, 100:0 to 90:10) to give 2,3-diamino-*N*-methyl-benzamide (357 mg, 34%) as a yellow oil.

^1^H NMR (500 MHz, CDCl_3_) δ 7.58 (s, 1H, H-11), 7.03 (s, 1H, H-9), 6.88 (d, *J* = 7.9 Hz, 1H, H-6), 6.77 – 6.68 (m, 1H, H-4), 6.64 (s, 1H, H-9), 6.55 (t, *J* = 7.8 Hz, 1H, H-5), 5.15 (s, 2H, H-12), 2.89 (d, *J* = 4.8 Hz, 3H, H-10); LCMS (LCQ) Rt = 0.5 min (4 min method); HRMS *m/z* (ESI)^+^ 165.9611 (M+H)^+^ calculated MW for C_8_H_11_N_3_O 165.1920.

***N*-[2-Amino-3-(methylcarbamoyl)phenyl]pyridine-4-carboxamide**

The intermediate was prepared *via* General Method C using 2,3-diamino-*N*-methyl-benzamide (140 mg, 1 eq.) and isonicotinic acid (1.1 eq.). *N*-[2-Amino-3-(methylcarbamoyl)-phenyl]pyridine-4-carboxamide (123 mg, 46%) was isolated by filtration as an off-white solid.

^1^H NMR (500 MHz, DMSO-*d*_6_) δ 9.93 (s, 1H, H-12), 8.77 (d, *J* = 5.2 Hz, 2H, H-2' and H-6'), 8.33 (s, 1H, H-9), 7.91 (d, *J* = 5.3 Hz, 2H, H-3' and H-5'), 7.44 (d, *J* = 7.5 Hz, 1H, H-6), 7.26 (d, *J* = 7.3 Hz, 1H, H-4), 6.60 (t, *J* = 7.6 Hz, 1H, H-5), 6.35 (s, 2H, H-11), 2.75 (d, *J* = 4.3 Hz, 3H, H-10); LCMS (LCQ) Rt = 0.6 min (4 min method); HRMS *m/z* (ESI)^+^ 293.1015 (M+Na)^+^, calculated MW for C_14_H_14_N_4_O_2_Na 293.2770.

***N*-Methyl-2-(4-pyridyl)-1*H*-benzimidazole-4-carboxamide (10)**

Compound **10** was prepared *via* General Method D using 2,3-diamino-*N*-methyl-benzamide (100 mg, 1 eq.). The reaction mixture was concentrated under reduced pressure. The crude was purified *via* flash column chromatography (11 g amino silica, ethyl acetate:methanol, 100:0 to 90:10) to give *N*-methyl-2-(4-pyridyl)-1*H*-benzimidazole-4-carboxamide (**10**) (11 mg, 6%) as a pale yellow solid.

^1^H NMR (500 MHz, DMSO-*d*_6_) δ 13.76 (s, 1H, H-1), 9.67 (s, 1H, H-10), 8.95 – 8.70 (m, 2H, H-2' and H-6'), 8.30 – 8.17 (m, 2H, H-3' and H-5'), 7.92 (s, 1H, H-7), 7.80 (d, *J* = 7.8 Hz, 1H, H-5), 7.41 (t, *J* = 7.5 Hz, 1H, H-6), 3.02 (s, 3H, H-11); LCMS (LCQ) Rt = 0.7 min (4 min method); HRMS *m/z* (ESI)^+^ 275.0912 (M+Na)^+^, calculated MW for C_14_H_12_N_4_ONa 275.2617.

***N*,*N*-Dimethyl-2-(4-pyridyl)-1*H*-benzimidazole-4-carboxamide (11)**^18^

2 M aqueous sodium hydroxide solution (9 mL, 2 eq.) was added to methyl 2-(4-pyridyl)-1*H*-benzimidazole-4-carboxylate (**15**) (2.39 g, 1 eq.) in methanol (1 mL). The resulting mixture was refluxed at 100 °C overnight. The mixture was concentrated under reduced pressure and sodium 2-(4-pyridyl)-1*H*-benzimidazole-4-carboxylate (3.38 g) was taken forward without further purification. Triethylamine (1 ml, 20 eq.), dimethylamine (0.19 ml, 1 eq.) 2 M in THF, sodium 2-(4-pyridyl)-1*H*-benzimidazole-4-carboxylate (100 mg, 1 eq.) and 50% propylphosphonic anhydride in ethyl acetate (0.34 ml, 3 eq.) were stirred together in *N*,*N*-dimethylformamide (5 ml) at RT overnight. The reaction mixture was concentrated under reduced pressure and the resulting crude yellow mixture (734 mg) was purified *via* flash column chromatography (30 g silica, ethyl acetate:methanol, 100:0 to 90:10) to give *N*,*N*-dimethyl-2-(4-pyridyl)-1*H*-benzimidazole-4-carboxamide (**11**) (34 mg, 32%) as an off-white solid.

^1^H NMR (600 MHz, DMSO-*d*_6_) δ 13.34 (s, 1H, H-1), 8.92 – 8.59 (m, 2H, H-2' and H-6'), 8.14 (s, 2H, H-3' and H-5'), 7.70 (s, 1H, H-7), 7.32 (t, *J* = 7.5 Hz, 1H, H-6), 7.22 (d, *J* = 7.2 Hz, 1H, H-5), 3.11 (s, 3H, H-11), 2.87 (s, 3H, H-11); LCMS (LCQ) Rt = 0.6 min (4 min method); HRMS *m/z* (ESI)^+^ 267.1241 (M+H)^+^, calculated MW for C_15_H_14_N_4_O 266.2973.

**3,4-Diaminobenzamide**

The intermediate was prepared *via* General Method B using 4-amino-3-nitrobenzamide (500 mg). 3,4-Diaminobenzamide (249 mg, 57%) was isolated as an off-white solid.

^1^H NMR (600 MHz, DMSO-*d*_6_) δ 7.36 (s, 1H, H-11), 7.05 (s, 1H, H-3), 6.97 (d, *J* = 8.0 Hz, 1H, H-5), 6.69 (s, 1H, H-11), 6.45 (d, *J* = 8.0 Hz, 1H, H-6), 4.92 (s, 2H, H-7), 4.49 (s, 1H, H-8); LCMS (LCQ) Rt = 0.5 min (4 min method); HRMS *m/z* (ESI)^+^ 152.0900 (M+H)^+^, calculated MW for C_7_H_9_N_3_O 151.1665.

***N*-(2-Amino-5-carbamoyl-phenyl)pyridine-4-carboxamide**

The intermediate was prepared *via* General Method C using 3,4-diaminobenzamide (196 mg, 1 eq.) and isonicotinic acid (1.1 eq.). The reaction mixture was filtered giving *N*-(2-amino-3-carbamoyl-phenyl)-pyridine-4-carboxamide (299 mg, 96%) as an off-white solid.

^1^H NMR (500 MHz, DMSO-*d*_6_) δ 9.91 (s, 1H, H-11), 8.77 (d, *J* = 5.3 Hz, 2H, H-2'and H-6'), 7.91 (d, *J* = 5.5 Hz, 2H, H-3'and H-5'), 7.87 (s, 1H, H-10), 7.53 (d, *J* = 7.9 Hz, 1H, H-6), 7.26 (d, *J* = 7.6 Hz, 1H, H-4), 7.23 (s, 1H, H-10), 6.57 (t, *J* = 7.8 Hz, 1H, H-5), 6.52 (s, 2H, H-9); LCMS (LCQ) Rt = 0.8 min (4 min method); HRMS *m/z* (ESI)^+^ 279.0848 (M+Na)^+^, calculated MW for C_13_H_12_N_4_O_2_Na 279.2504.

**2-(4-Pyridyl)-1*H*-benzimidazole-4-carboxamide (12)**

Compound **12** was prepared *via* General Method D using *N*-(2-aminophenyl)-pyridine-4-carboxamide (100 mg, 1 eq.). The reaction mixture was concentrated under reduced pressure to give a pale yellow crude solid (37 mg). The crude was purified *via* flash column chromatography (11 g amino silica, ethyl acetate:methanol, 100:0 to 85:15) to give 2-(4-pyridyl)-1*H*-benzimidazole-4-carboxamide (**12**) (50 mg, 25%) as a yellow solid.

^1^H NMR (500 MHz, DMSO-*d*_6_) δ 9.19 (s, 2H, H-10), 8.80 (d, *J* = 4.3 Hz, 2H, H-2'and H-6'), 8.19 (d, *J* = 4.3 Hz, 2H, H-3'and H-5'), 7.91 (d, *J* = 7.3 Hz, 1H, H-7), 7.84 (s, 1H, H-1), 7.81 (d, *J* = 7.9 Hz, 1H, H-5), 7.41 (t, *J* = 7.7 Hz, 1H, H-6); LCMS (LCQ) Rt = 0.6 min (4 min method); HRMS *m/z* (ESI)^+^ 261.0741 (M+Na)^+^, calculated MW for C_13_H_10_N_4_ONa 261.2351.

***N*-(2-Aminophenyl)pyridine-4-carboxamide**

The intermediate was prepared *via* General Method C using *O*-phenylenediamine (100 mg, 1 eq.) and isonicotinic acid (1.1 eq.). The reaction mixture was filtered giving *N*-(2-aminophenyl)pyridine-4-carboxamide (156 mg, 75%) as an off-white solid.

^1^H NMR (500 MHz, DMSO-*d*_6_) δ 9.90 (s, 1H, H-8), 8.76 (d, *J* = 4.6 Hz, 2H, H-2' and H-6'), 7.89 (d, *J* = 4.6 Hz, 2H, H-3' and H-5'), 7.16 (d, *J* = 7.6 Hz, 1H, H-3), 6.99 (t, *J* = 7.5 Hz, 1H, H-5), 6.78 (d, *J* = 7.9 Hz, 1H, H-6), 6.59 (t, *J* = 7.4 Hz, 1H, H-4), 5.01 (s, 2H, H-7); LCMS (LCQ) Rt = 0.7 min (4 min method); HRMS *m/z* (ESI)^+^ 214.1100 (M+H)^+^, calculated MW for C_12_H_11_N_3_O 213.2348.

**2-(4-Pyridyl)-1*H*-benzimidazole (13)**^19–21^

Compound **13** was prepared *via* General Method D using *N*-(2-amino-5-carbamoyl-phenyl)pyridine-4-carboxamide (30 mg). The reaction mixture was concentrated under reduced pressure to give an off-white solid (181 mg). The crude was purified *via* flash column chromatography (5 g amino silica, ethyl acetate:methanol, 100:0 to 90:10) to give 2-(4-pyridyl)-1*H*-benzimidazole (**13**) (64 mg, 66%) as a white solid.

^1^H NMR (500 MHz, DMSO-*d*_6_) δ 13.28 (s, 1H, H-1), 8.76 (d, *J* = 5.7 Hz, 2H, H-2' and H-6'), 8.10 (d, *J* = 5.8 Hz, 2H, H-3' and H-5'), 7.73 (s, 1H, H-4/7), 7.61 (s, 1H, H-7/4), 7.27 (s, 2H, H-5 and H-6); LCMS (LCQ) Rt = 0.6 min (4 min method); HRMS *m/z* (ESI)^+^ 196.0871 (M+H)^+^, calculated MW for C_12_H_9_N_3_ 195.2196.

***N*-(2-Amino-3-nitro-phenyl)pyridine-4-carboxamide**

The intermediate was prepared *via* General Method C using 3-nitrobenzene-1,2-diamine (500 mg). The reaction mixture was filtered and *N*-(2-amino-3-nitro-phen-yl)pyridine-4-carboxamide (360 mg, 28%) was isolated as a brown solid. The product was taken forward without further purification.

^1^H NMR (500 MHz, DMSO-*d*_6_) δ 10.13 (s, 1H, H-7), 8.79 (s, 2H, H-2' and H-6'), 8.01 (d, *J* = 7.8 Hz, 1H, H-4), 7.94 (s, 2H, H-3' and H-5'), 7.54 – 7.43 (m, 1H, H-6), 7.28 (s, 2H, H-10), 6.75 – 6.63 (m, 1H, H-5); LCMS (LCQ) Rt = 0.6 min (4 min method); HRMS *m/z* (ESI)^+^ 259.0815 (M+H)^+^, calculated MW for C_12_H_10_N_4_O_3_ 258.2324.

**4-Nitro-2-(4-pyridyl)-1*H*-benzimidazole (14)**

Compound **14** was prepared *via* General Method D using *N*-(2-amino-3-nitro-phenyl)pyridine-4-carboxamide (323 mg). The reaction mixture was concentrated under reduced pressure to give a pale yellow solid (114 mg). The crude was purified *via* flash column chromatography (4.7 g amino silica, ethyl acetate:methanol, 100:0 to 90:10) to give 4-nitro-2-(4-pyridyl)-1*H*-benzimidazole (**14**) (294 mg, 85%) as an orange solid.

^1^H NMR (500 MHz, DMSO-*d*_6_) δ 8.79 (d, *J* = 4.1 Hz, 2H, H-2' and H-6'), 8.29 (d, *J* = 4.1 Hz, 2H, H-3' and H-5'), 8.18 (m, 2H, H-5 and H-7), 7.47 (t, *J* = 7.0 Hz, 1H, H-6); LCMS (LCQ) Rt = 0.8 min (4 min method), *m/z* (ESI)^+^ 262.0541 (M+Na)^+^, calculated MW for C_12_H_7_N_4_O_2_Na 262.2000.

**Methyl 3-amino-2-(pyridine-4-carbonyl-amino)benzoate**

The intermediate was prepared *via* General Method C using 2,3-diaminobenzoic acid methyl ester (2 g, 1 eq.) and isonicotinic acid (1.1 eq.). The reaction mixture was concentrated under reduced pressure and water (100 mL) was added to the residue. The aqueous solution was extracted three times with ethyl acetate (3 × 100 mL). The combined organic phases were dried over MgSO_4_, filtered and concentrated under reduced pressure to give an orange oil (692 mg). The crude was purified *via* flash column chromatography (24 g silica, ethyl acetate:methanol, 100:0 to 90:10) to give methyl 3-amino-2-(pyridine-4-carbonylamino)benzoate (3.42 g, 94%) as an orange oil which crystallised upon standing to form a pale orange solid. The product was taken forward without further purification.

^1^H NMR (500 MHz, DMSO-*d*_6_) δ 9.99 (s, 1H, H-7), 8.78 (d, *J* = 4.4 Hz, 2H, H-2' and H-6'), 7.92 (d, *J* = 4.4 Hz, 2H, H-3' and H-5'), 7.74 (d, *J* = 8.0 Hz, 1H, H-4), 7.37 (d, *J* = 7.4 Hz, 1H, H-6), 6.63 (d, *J* = 8.6 Hz, 3H, H-10), 3.82 (s, 3H, H-14); LCMS (LCQ) Rt = 0.8 min (7 min method); HRMS *m/z* (ESI)^+^ 272.0200 (M+H)^+^, calculated MW for C_14_H_13_N_3_O_3_ 271.2708.

**Methyl 2-(4-pyridyl)-1*H*-benzimidazole-4-carboxylate (15)**

Compound **15** was prepared *via* General Method D using methyl 2-amino-3-(pyridine-4-carbonylamino) benzoate (139 mg). The reaction mixture was concentrated under reduced pressure. The crude was purified *via* flash column chromatography (11 g amino silica, ethyl acetate:methanol, 100:0 to 90:10) to give methyl 2-(4-pyridyl)-1*H*-benzimida-zole-4-carboxylate (**15**) (118 mg, 86%) as a pale yellow solid.

^1^H NMR (500 MHz, DMSO-*d*_6_) δ 8.76 (d, *J* = 4.2 Hz, 2H, H-2' and H-6'), 8.26 (d, *J* = 4.1 Hz, 2H, H-3' and H-5'), 8.01 (d, *J* = 7.8 Hz, 1H, H-7), 7.88 (d, *J* = 7.3 Hz, 1H, H-5), 7.37 (t, *J* = 7.5 Hz, 1H, H-6), 4.02 – 3.92 (m, 3H, H-11); LCMS (LCQ) Rt = 0.7 min (7 min method); HRMS *m/z* (ESI)^+^ 254.0920 (M+H)^+^, calculated MW for C_14_H_11_N_3_O_2_ 253.2556.

**Ethyl 4-amino-3-(pyridine-4-carbonyl-amino)benzoate**

The intermediate was prepared *via* General Method C using ethyl 3,4-diaminobenzoate (100 mg) and isonicotinic acid (1.1 eq.). The reaction mixture was concentrated under reduced pressure and water (5 mL) was added to the residue. The aqueous solution was extracted three times with ethyl acetate (3 × 5 mL). The combined organic phases were dried over MgSO_4_, filtered and concentrated under reduced pressure to give an orange oil (191 mg). The crude was purified *via* flash column chromatography (12 g silica, ethyl acetate:methanol, 100:0 to 90:10) to give ethyl 4-amino-3-(pyridine-4-carbonyl-amino)benzoate (151 mg, 91%) as a pale orange solid.

^1^H NMR (500 MHz, DMSO-*d*_6_) δ 9.92 (s, 1H, H-12), 8.77 (d, *J* = 4.2 Hz, 2H, H-2' and H-6'), 7.91 (d, *J* = 4.4 Hz, 2H, H-3' and H-5'), 7.75 (s, 1H, H-2), 7.61 (d, *J* = 8.5 Hz, 1H, H-6), 6.77 (d, *J* = 8.5 Hz, 2H, H-5), 5.93 (s, 2H, H-15), 4.22 (q, *J* = 7.1 Hz, 2H, H-10), 1.42 – 0.97 (m, 3H, H-11); LCMS (LCQ) Rt = 0.6 min (4 min method); HRMS *m/z* (ESI)^+^ 308.1007 (M+Na)^+^, calculated MW for C_15_H_15_N_3_O_3_Na 308.2884.

**Ethyl 2-(4-pyridyl)-1*H*-benzimidazole-5-carboxylate (16)**^22,23^

Compound **16** was prepared *via* General Method D using ethyl 4-amino-3-(pyridine-4-carbonyl-amino) benzoate (130 mg, 1 eq.). The reaction mixture was concentrated under reduced pressure to give an off-white solid (170 mg). The crude was purified *via* flash column chromatography (5 g amino silica, ethyl acetate:methanol, 100:0 to 90:10) to give ethyl 2-(4-pyridyl)-1*H*-benzimidazole-5-carboxylate (**16**) (50 mg, 58%) as a white solid.

^1^H NMR (500 MHz, DMSO-*d*_6_) δ 8.72 (d, *J* = 4.7 Hz, 2H (H-2' and H-6'), 8.24 (s, 1H, H-4), 8.16 (d, *J* = 4.9 Hz, 2H, H-3' and H-5'), 7.80 (d, *J* = 8.4 Hz, 1H, H-6), 7.69 (d, *J* = 8.4 Hz, 1H, H-7), 4.32 (q, *J* = 7.0 Hz, 2H, H-11), 1.34 (t, *J* = 7.1 Hz, 3H, H-12); LCMS (LCQ) Rt = 0.6 min (4 min method); HRMS *m/z* (ESI)^+^ 290.0904 (M+Na)^+^, calculated MW for C_15_H_13_N_3_O_2_Na 290.2731.

***N-*(2-Amino-5-cyano-phenyl)pyridine-4-carboxamide**

The intermediate was prepared *via* General Method C using 3,4-diaminobenzonitrile (100 mg, 1 eq.). The reaction mixture was filtered and *N*-(2-amino-5-cyano-phenyl)pyridine-4-carboxamide (114 mg, 57%) was isolated as a brown solid.

^1^H NMR (500 MHz, DMSO-*d*_6_) δ 9.96 (s, 1H, H-7), 8.77 (d, *J* = 4.2 Hz, 2H, H-2' and H-6'), 7.90 (d, *J* = 4.4 Hz, 2H, H-3' and H-5'), 7.55 (s, 1H, H-3), 7.38 (d, *J* = 8.1 Hz, 1H, H-6), 6.81 (d, *J* = 8.4 Hz, 1H, H-5), 6.15 (s, 2H, H-10); LCMS (LCQ) Rt = 0.5 min (4 min method); HRMS *m/z* (ESI)^+^ 499.1607 (dimer + Na)^+^, calculated MW for (C_13_H_10_N_4_O)_2_Na 499.4805.

**2-(4-Pyridyl)-1*H*-benzimidazole-5-carbonitrile (17)**

Compound **17** was prepared *via* General Method D using *N*-(2-amino-5-cyano-phenyl)pyridine-4-carboxamide (90 mg, 1 eq.). The reaction mixture was concentrated under reduced pressure to give an off-white solid (103 mg). The crude was purified *via* flash column chromatography (4.7 g amino silica, ethyl acetate:methanol, 100:0 to 90:10) to give 2-(4-pyridyl)-1*H*-benzimidazole-5-carbonitrile (**17**) (28 mg, 34%) as a pale pink solid.

^1^H NMR (500 MHz, DMSO-*d*_6_) δ 8.79 (d, *J* = 4.1 Hz, 2H, H-2' and H-6'), 8.23 (s, 1H, H-4), 8.11 (d, *J* = 4.1 Hz, 2H, H-3' and H-5'), 7.81 (d, *J* = 8.1 Hz, 1H, H-7), 7.65 (d, *J* = 8.1 Hz, 1H, H-6); LCMS (LCQ) Rt = 0.6 min (4 min method); HRMS *m/z* (ESI)^+^ 221.0817 (M+H)^+^, calculated MW for C_13_H_8_N_4_ 220.2291.

***N*-(2-Amino-5-nitro-phenyl)pyridine-4-carboxamide**

The intermediate was prepared *via* General Method C using 4-nitro-*O*-phenylene-diamine (150 mg, 1 eq.). The reaction mixture was filtered and *N*-(2-amino-5-nitro-phenyl)-pyridine-4-carboxamide (146 mg, 52%) was isolated as a brown solid. The product was taken forward without further purification.

^1^H NMR (500 MHz, DMSO-*d*_6_) δ 10.03 (s, 1H, H-7), 8.88 – 8.70 (m, 2H, H-2' and H-6'), 8.12 (s, 1H, H-6), 8.00 – 7.86 (m, 3H, H-4, H-3' and H-5'), 6.80 (d, *J* = 8.9 Hz, 1H, H-3), 6.70 (s, 2H, H-10); LCMS (LCQ) Rt = 0.5 min (4 min method), HRMS *m/z* (ESI)^+^ 257.0670 (M-H)^+^, calculated MW for C_12_H_10_N_4_O_3_ 258.2324.

**5-Nitro-2-(4-pyridyl)-1*H*-benzimidazole (18)**

Compound **18** was prepared *via* General Method D using *N*-(2-amino-5-nitro-phenyl)-pyridine-4-carboxamide (117 mg). The reaction mixture was concentrated under reduced pressure to give an off-white solid (130 mg). The crude was purified *via* flash column chromatography (4.7 g amino silica, ethyl acetate:methanol, 100:0 to 90:10) to give 5-nitro-2-(4-pyridyl)-1*H*-benzimidazole (**18**) (63 mg, 0.24 mmol, 58%) as a pale orange solid.

^1^H NMR (500 MHz, DMSO-*d*_6_) δ 8.81 (d, *J* = 4.9 Hz, 2H, H-2' and H-6'), 8.54 (s, 1H, H-4), 8.17 (d, *J* = 8.9 Hz, 1H, H-6), 8.12 (d, *J* = 4.8 Hz, 2H, H-3' and H-5'), 7.83 (d, *J* = 8.8 Hz, 1H, H-7); LCMS (LCQ) Rt = 0.6 min (4 min method); HRMS *m/z* (ESI)^+^ 241.0720 (M+H)^+^, calculated MW for C_12_H_8_N_4_O_2_ 240.2172.

**2-Amino-5-bromo-3-nitro-benzamide**

The intermediate was prepared *via* General Method A using 2-amino-5-bromo-3-nitrobenzoic acid (900 mg, 1 eq.). The reaction mixture was filtered giving 2-amino-5-bromo-3-nitro-benzamide (330 mg, 35%) as a fine orange solid. The filtrate was partially concentrated under reduced pressure, left overnight and a further batch of the product (150 mg, 16%) was isolated by filtration.

^1^H NMR (500 MHz, DMSO-*d*_6_) δ 8.49 (s, 2H, H-9), 8.29 (d, *J* = 2.3 Hz, 1H, H-4), 8.27 (s, 1H, H-10), 8.08 (d, *J* = 2.3 Hz, 1H, H-6), 7.76 (s, 1H, H-10); LCMS (MDAP) Rt = 1.9 min (Ana 5–95 in 5 min); HRMS *m/z* (ESI)^+^ 257.8000 [Br-79] and 259.8000 [Br-81] (M-H)^+^, calculated MW for C_7_H_6_BrN_3_O_3_ 260.0446.

**2,3-Diamino-5-bromo-benzamide**

The intermediate was prepared *via* General Method B using 2-amino-5-bromo-3-nitro-benzamide (150 mg, 1 eq.). 2,3-Diamino-5-bromo-benzamide (69 mg, 52%) was isolated as a pale-yellow solid.

^1^H NMR (500 MHz, DMSO-*d*_6_) δ 7.74 (s, 1H, H-10), 7.11 (s, 1H, H-10), 7.03 (s, 1H, H-6), 6.73 (s, 1H, H-4), 6.23 (s, 2H, H-9), 4.99 (s, 2H, H-11); LCMS (MDAP) Rt = 25.1 min (Ana 5–95 in 20 min; HRMS *m/z* (ESI)^+^ 251.9743 [Br-79] and 253.9722 [Br-81] (M+Na)^+^, calculated MW for C_7_H_8_BrN_3_ONa 253.0516.

***N*-(2-Amino-5-bromo-3-carbamoyl-phenyl)pyridine-4-carboxamide**^24^

2,3-diamino-5-bromo-benzamide (750 mg, 1 eq.), isonicotinic acid (1 eq.), 1-hydroxybenzotriazole hydrate (1 eq.) and *N*-(3-dimethylaminopropyl)-*N’*-ethylcarbodiimide hydrochloride (1.2 eq.) in DMF (7 mL) were stirred overnight at 100 °C. The reaction was allowed to cool to room temperature and saturated aqueous Na_2_CO_3_ was added until effervescence ceased (*ca.* 10–15 mL). A brown precipitate formed. The mixture was filtered, and the collected solids were dried in a vacuum oven overnight. The resulting brown solid (825 mg) was purified *via* flash column chromatography (40 g silica, ethyl acetate:methanol, 100:0 to 90:10) to give *N*-(2-amino-5-bromo-3-carbamoyl-phenyl)pyridine-4-carboxamide (825 mg, 52%) as a pale yellow solid.

^1^H NMR (600 MHz, DMSO-*d*_6_) δ 9.95 (s, 1H, H-11), 8.78 (d, *J* = 4.6 Hz, 2H, H-2' and H-6'), 8.20 (s, 1H, H-10), 8.00 (s, 1H, H-10), 7.90 (d, *J* = 4.0 Hz, 2H, H-3' and H-5'), 7.71 (s, 1H, H-6), 7.46 (s, 1H, H-4), 6.66 (s, 2H, H-9); LCMS (LCQ) Rt = 0.6 min (4 min method); HRMS *m/z* (ESI)^+^ 317.0038 (product cyclised to **19** on column)^+^, LRMS (LCQ) *m/z* (ESI)^+^ 334.97 [Br-79], 336. 95 [Br-81], 317.16 (product cyclised to **19** on column) (M+H)^+^, calculated MW for C_13_H_11_BrN_4_O_2_ 335.1556.

**6-Bromo-2-(4-pyridyl)-1*H*-benzimidazole-4-carboxamide (19)**

Compound **19** was prepared *via* General Method D using *N*-(2-amino-5-bromo-3-carbamoyl-phenyl)pyridine-4-carboxamide (300 mg). The reaction mixture was neutralised to pH 8 with saturated aqueous sodium carbonate and the resulting suspension was filtered. The collected solids were dried in a vacuum oven overnight to yield 6-bromo-2-(4-pyridyl)-1*H*-benzimidazole-4-carboxamide (**19**) (300 mg, 100%) as a pale brown solid.

^1^H NMR (600 MHz, DMSO-*d*_6_) δ 10.09 (d, *J* = 4.2 Hz, 1H, H-10), 8.59 – 8.44 (m, 2H, H-2' and H-6'), 8.13 (d, *J* = 6.0 Hz, 2H, H-3' and H-5'), 7.69 (s, 1H, H-5), 7.57 (s, 1H, H-7), 7.45 (d, *J* = 3.6 Hz, 1H, H-10); ^13^C NMR (151 MHz, DMSO-*d*_6_) δ 175.1 (C-8), 167.5 (C-6), 159.5 (C-2), 150.1 (C-2' and C-6'), 149.9 (C-4), 145.4 (C-3a), 143.5 (C-4'), 122.3 (C-5), 122.1 (C-7), 121.4 (C-3' and C-5'), 110.5 (C-7a); LCMS (MDAP) Rt = 2.9 min (Ana 5–95 over 5 min); HRMS *m/z* (ESI)^+^ 316.1959 [Br-79], 318.8059 [Br-81] (M+H)^+^, calculated MW for C_13_H_9_BrN_4_O 317.1404.

**Cloning, protein expression and purification**

PKN2- Uniprot ID Q16513 (Ser646-Cys984) and PKN1-Uniprot ID Q16512 (Pro604-Cys942) were cloned fused to an N-terminal GST tag and C-terminal biotin tag attachment site (SSKGGYGLNDIFEAQKIEWHE).

Bacmid DNA containing the construct sequence was prepared from DH10Bac cells and used to transfect *Sf*9 insect cells for the preparation of baculovirus. The prepared baculovirus was used to infect a new batch of *Sf*9 cells cultivated in Sf-900 II SFM media (Thermo Fisher) in the presence of 20 U/mL of penicillin/streptomycin. Both proteins were expressed for 48 hours at 27°C. Additional biotin (Sigma-Aldrich) was added to the culture (200 mM/L) for biotinylation by co-expressed BirA. After incubation the cells were harvested (900 x *g*, 30 min, 4°C) and were re-suspended in lysis buffer (50 mM HEPES pH 7.5, 300 mM NaCl, 5% glycerol, 0.5 mM TCEP, 5 µL per 1 mL protease inhibitor cocktail Set III EDTA-free (Merckmillipore), frozen in liquid nitrogen and stored for later purification. The cells were thawed, re-suspended in approximately 150 mL lysis buffer, sonicated for 2 min in total (5 s ON, 10 s OFF) on ice. After sonication, the lysate was supplemented with 0.7% of Tween20 and benzonase (50 μg/100 mL of lysate) and incubated on ice for 15 min. The cell lysates were centrifuged at 50000 x *g* for 30 min at 4 °C. The supernatant was loaded on a pre-equilibrated (with lysis buffer) gravity column with 5 mL of glutathione-Sepharose resin and then purified by affinity chromatography. After loading the sample, the resin was washed with 40 CV of lysis buffer. The sample was eluted with 10 CV of lysis buffer containing 10 mM reduced glutathione. The elution fraction was concentrated to 2 mL using a 30 kDa molecular weight cut-off centrifugal concentrator (Millipore) at 4 °C and injected onto an S75 16/600 column (pre-equilibrated in 20 mM Hepes pH 7.5, 300 mM NaCl, 5% Glycerol, 1 mM TCEP) at 1.0 mL/min. 1.5 mL fractions were collected and analysed on a 12% SDS-PAGE. One sample of the protein was separated to confirm its identity and the biotinylation by intact mass analysis. The main peak fractions were concentrated, and the protein was flash-frozen in a liquid nitrogen bath and stored at -80 °C until use.

**Binding Displacement Assay**

The time resolved fluorescence resonance energy transfer (TR-FRET) method was used to measure the binding of a fluorescent tracer molecule in the ATP binding site. Displacement of the tracer by varying concentrations of an inhibitor allows the IC_50_ value for the inhibitor to be calculated. Compounds were serially diluted from 4 mM to 22 nM for a 12-point (1:3) in DMSO and then diluted in assay buffer (50 mM HEPES pH 7.5, 10 mM MgCl_2_, 1 mM EGTA, 0.01% Brij-35). The dissociation constants of the tracer were measured for both kinases (PKN2 *K*_D_ = 5 ± 0.2 nM and PKN1 *K*_D_ = 2.8 ± 0.2 nM, Supplementary Figure 1). The final assay contained 5 nM biotinylated PKN2 or 15 nM biotinylated PKN1 ligated to streptavidin-Tb-cryptate (Cisbio), 5 nM or 2.8 nM Kinase Tracer 236 (Thermo Fisher Scientific) respectively for PKN2 and PKN1 and serial dilutions of the compounds. Final assay volume of each data point was 15 µL and final DMSO concentration was 1.25%. Black 384 well plates (Greiner) were used. The assay was incubated at room temperature for 1 hour and then TR-FRET data was measured on a CLARIOstar Plus (BMG Labtech). Data was normalized to 0% and 100% inhibition values and fit to a four parameter dose-response binding curve in GraphPad 7 Software (version 7.04). All the points were measured in triplicate with two biological replicates. 10 µM of Staurosporine was used as a control for 100% inhibition and calculation of the Z’ value.^25^


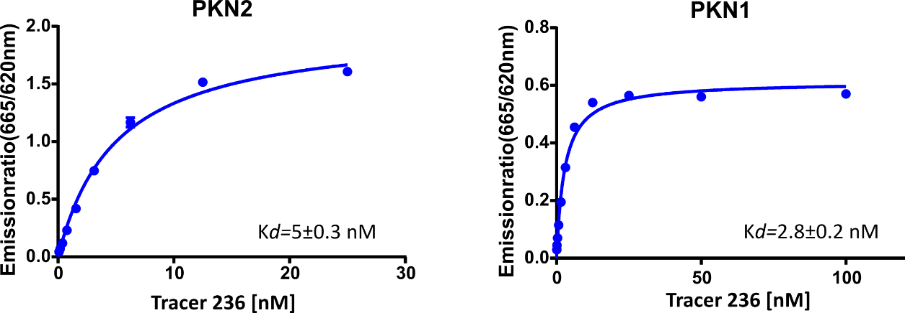


**Figure S1.** Determination of dissociation constant of Tracer 236 (ThermoFisher) when titrated over 5 nM of PKN2 or 15 nM of PKN1.

1. Francis C. Rix, Smita Kacker, Sudhin Datta, Rul Zhao VRE. Olefin polymerization catalyst system and process for use thereof. 2005. http://www.google.ch/patents/US7601666. Accessed May 4, 2017.

2. Schmidt A, Shilabin AG, Nieger M, Mariand M, Levillain P, Sense JM. On benzo[b][1,4]diazepinium-olates, -thiolates and -carboxylates as anti-Hückel mesomeric betaines. *Org Biomol Chem*. 2003;1(23):4342-4350. doi:10.1039/B308412D

3. Van Steijvoort BF, Kaval N, Kulago AA, Maes BUW. Remote Functionalization: Palladium-Catalyzed C5(sp ^3^ )-H Arylation of 1-Boc-3-aminopiperidine through the Use of a Bidentate Directing Group. *ACS Catal*. 2016;6(7):4486-4490. doi:10.1021/acscatal.6b00841

4. White AW, Almassy R, Calvert AH, et al. Resistance-Modifying Agents. 9. 1 Synthesis and Biological Properties of Benzimidazole Inhibitors of the DNA Repair Enzyme Poly(ADP-ribose) Polymerase. *J Med Chem*. 2000;43(22):4084-4097. doi:10.1021/jm000950v

5. Goodman KB, Cui H, Dowdell SE, et al. Development of dihydropyridone indazole amides as selective Rho-kinase inhibitors. *J Med Chem*. 2007;50(1):6-9. doi:10.1021/jm0609014

6. Chen X, Jia H, Li Z, Xu X. Synthesis and nematicidal evaluation of 1,2,3-benzotriazin-4-one derivatives containing piperazine as linker against Meloidogyne incognita. *Chinese Chem Lett*. 2019;30(6):1207-1213. DOI: 10.1016/j.cclet.2019.02.033

7. Zhang H, Liu H, Luo X, et al. Design, synthesis and biological activities of 2,3-dihydroquinazolin-4(1H)-one derivatives as TRPM2 inhibitors. *Eur J Med Chem*. 2018;152:235-252. DOI: 10.1016/j.ejmech.2018.04.045

8. Velagapudi UK, Langelier MF, Delgado-Martin C, et al. Design and Synthesis of Poly(ADP-ribose) Polymerase Inhibitors: Impact of Adenosine Pocket-Binding Motif Appendage to the 3-Oxo-2,3-dihydrobenzofuran-7-carboxamide on Potency and Selectivity. *J Med Chem*. 2019;62(11):5330-5357. DOI: 10.1021/acs.jmedchem.8b01709

9. Zhang LJ, Yang K, Li CY, Sun YQ. A simple and metal-free one-pot synthesis of 2-substituted-1H-4-carboxamide benzimidazole using 3,6-di(pyridin-2-yl)-1,2,4,5-tetrazine(PYTZ) as catalyst. *Chem Pap*. 2019;73(11):2697-2705. DOI: 10.1007/s11696-019-00821-x

10. Isbera M, Bognor B, Gulys-Fekete G, Kish K, Klai T. Syntheses of Pyrazine-, Quinoxaline-, and Imidazole-Fused Pyrroline Nitroxides. *Synthesis (Stuttg)*. 2019;51(23):4463-4472. DOI: 10.1055/s-0039-1690678

11. Frackenpohl J, Heinemann I, et al. Use of Substituted 2-Amidobenzimidazoles, 2-amidobenzoxazoles and 2-amidobenzothiazoles or salts thereof as active substances against abiotic plant stress. 2015;(US2015/216168)..

12. Metz JT, Johnson EF, Soni NB, Merta PJ, Kifle L, Hajduk PJ. Navigating the kinome. *Nat Chem Biol*. 2011;7(4):200-202. doi:10.1038/nchembio.530

13. Lubisch W; Kock M; Hoeger T; Grandel R; Holzenkamp U; Schult S; Mueller R. Heterocyclically substituted benzimidazoles, the production and application thereof. 2004. https://worldwide.espacenet.com/publicationDetails/biblio?CC=US&NR=6696437&KC=&FT=E&locale=en_EP. Accessed January 17, 2020.

14. Kazuhisa T;Yuji K; Naoyuki M; Yoji M; Takenori K; Shinya N; Yoshinori O; Yohei O; Makoto T. Benzimidazole Derivatives . 2001. https://worldwide.espacenet.com/publicationDetails/biblio?CC=WO&NR=0121615&KC=&FT=E&locale=en_EP. Accessed January 17, 2020.

15. Michael K; Wilfried L; Axel J. Use of Parp Inhibitors in Cosmetic Preparations . 2001. https://worldwide.espacenet.com/publicationDetails/biblio?CC=WO&NR=0182877&KC=&FT=E&locale=en_EP. Accessed January 17, 2020.

16. Tsukamoto G, Yoshino K, Kohno T, Ohtaka H, Kagaya H, Ito K. 2-Substituted azole derivatives. 1. Synthesis and antiinflammatory activity of some 2-(substituted-pyridinyl)benzimidazoles. *J Med Chem*. 1980;23(7):734-738. doi:10.1021/jm00181a007

17. Xue F, Luo X, Ye C, Ye W, Wang Y. Inhibitory properties of 2-substituent-1H-benzimidazole-4-carboxamide derivatives against enteroviruses. *Bioorganic Med Chem*. 2011;19(8):2641-2649. doi:10.1016/j.bmc.2011.03.007

18. Hiroaki T; Ayako B; Makoto H;Yuji I; Yohei K; Madoka K. Azole-Substituted Pyridine Compound. 2018. https://worldwide.espacenet.com/publicationDetails/biblio?CC=EP&NR=3418276&KC=&FT=E&locale=en_EP. Accessed January 18, 2020.

19. Salehi N, Mirjalili BBF, Nadri H, et al. Synthesis and biological evaluation of new N-benzylpyridinium-based benzoheterocycles as potential anti-Alzheimer’s agents. *Bioorg Chem*. 2019;83:559-568. DOI: 10.1016/j.bioorg.2018.11.010

20. Raj P, Singh A, Singh A, et al. Pyrophosphate Prompted Aggregation-Induced Emission: Chemosensor Studies, Cell Imaging, Cytotoxicity, and Hydrolysis of the Phosphoester Bond with Alkaline Phosphatase. *Eur J Inorg Chem*. 2019;2019(5):628-638. DOI: 10.1002/ejic.201801173

21. Onyeachu IB, Obot IB, Sorour AA, Abdul-Rashid MI. Green corrosion inhibitor for oilfield application I: Electrochemical assessment of 2-(2-pyridyl) benzimidazole for API X60 steel under sweet environment in NACE brine ID196. *Corros Sci*. 2019;150:183-193. DOI: 10.1016/j.corsci.2019.02.010

22. Moszczyski-Potkowski R, Majer J, Borkowska M, et al. Synthesis and characterization of novel classes of PDE10A inhibitors - 1H-1,3-benzodiazoles and imidazo[1,2-a]pyrimidines. *Eur J Med Chem*. 2018;155:96-116. DOI: 10.1016/j.ejmech.2018.05.043

23. Berard CL, Lyon HCDE, UCBL, et al., Eds. Benzoimidazole DerivativesaAs Anticancer Agents. 2018;(WO2018/54989).

24. Yoshikawa M, Motoshima K, Fujimoto K, Tai A, Kakuta H, Sasaki K. Pyridinium cationic-dimer antimalarials, unlike chloroquine, act selectively between the schizont stage and the ring stage of Plasmodium falciparum. *Bioorg Med Chem*. 2008;16(11):6027-6033. doi:10.1016/J.BMC.2008.04.051

25. Zhang JH, Chung TDY, Oldenburg KR. A simple statistical parameter for use in evaluation and validation of high throughput screening assays. *J Biomol Screen*. 1999;4(2):67-73. doi:10.1177/108705719900400206
