# Supplementary material for: Development of 2-(4-pyridyl)-benzimidazoles as PKN2 chemical tools to probe cancer
Source: Bioorg Med Chem Lett. 2020 Apr 15;30(8):127040. doi: 10.1016/j.bmcl.2020.127040 (PMC7078758; doi:10.1016/j.bmcl.2020.127040)
Supplement: Supplementary data 2 [file mmc2.pdf]

PROTON_01

Compound 2

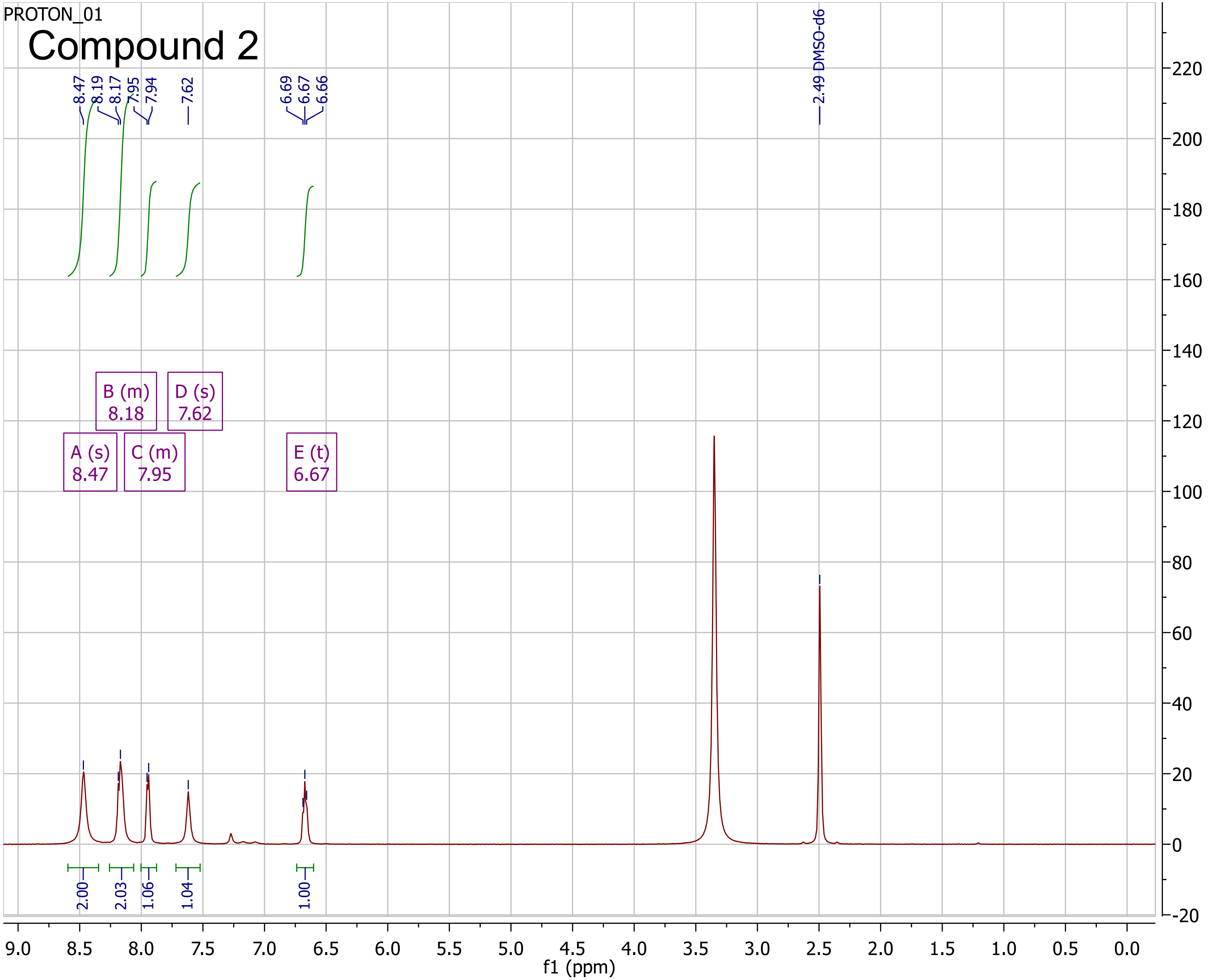

Sussex Drug Discovery Centre

	Parameter	Value
1	Data File Name	Y:/ walkup/ sew/ 20170327/ N2119-37-1_01/ PROTON_01.fid/ fid
2	Title	PROTON_01
3	Comment	
4	Origin	Varian
5	Owner	
6	Site	
7	Spectrometer	vnmrs
8	Author	
9	Solvent	dmso
10	Temperature	30.0
11	Pulse Sequence	s2pul
12	Experiment	1D
13	Probe	P8898_walkup
14	Number of Scans	8
15	Receiver Gain	30
16	Relaxation Delay	1.0000
17	Pulse Width	4.3000
18	Presaturation Frequency	
19	Acquisition Time	2.0447
20	Acquisition Date	2017-03-27T17:24:56
21	Modification Date	2017-03-27T17:25:52
22	Class	
23	Spectrometer Frequency	499.91
24	Spectral Width	8012.8

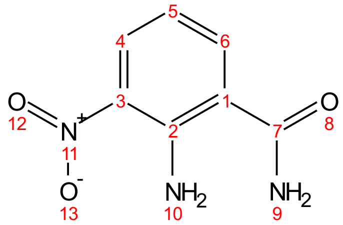

^{1}H NMR (500 MHz, DMSO-d_6) δ 8.47 (s, 2H, H-9), 8.25 – 8.07 (m, 2H, H-4 and H-10), 8.01 – 7.86 (m, 1H, H-6), 7.62 (s, 1H, H-10), 6.67 (t, J = 7.4 Hz, 1H, H-5).

Sussex Drug Discovery Centre

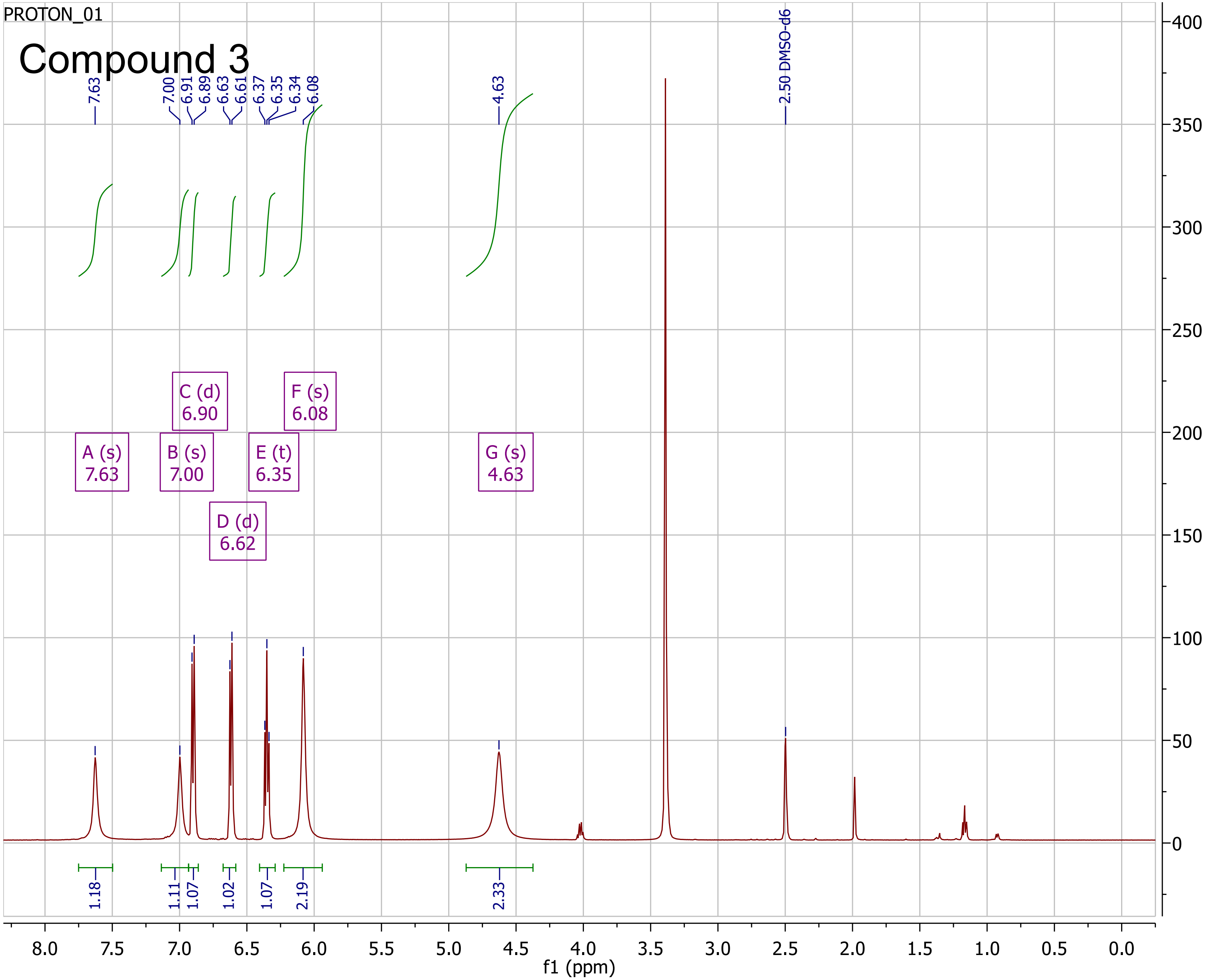

	Parameter	Value
1	Data File Name	Y:/ walkup/ sew/ 20170330/ N2119-40-Fr26-84_01/ PROTON_01.fid/ fid
2	Title	PROTON_01
3	Comment	
4	Origin	Varian
5	Owner	
6	Site	
7	Spectrometer	vnmrs
8	Author	
9	Solvent	dmso
10	Temperature	30.0
11	Pulse Sequence	s2pul
12	Experiment	1D
13	Probe	P8898_walkup
14	Number of Scans	8
15	Receiver Gain	24
16	Relaxation Delay	1.0000
17	Pulse Width	4.3000
18	Presaturation Frequency	
19	Acquisition Time	2.0447
20	Acquisition Date	2017-03-30T12:14:39
21	Modification Date	2017-03-30T12:15:07
22	Class	

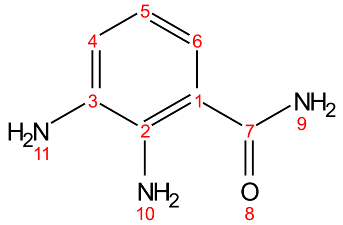

^{1}H NMR (500 MHz, DMSO-d_6) δ 7.63 (s, 1H, H-10), 7.00 (s, 1H, H-10), 6.90 (d, J = 8.0 Hz, 1H, H-6), 6.62 (d, J = 7.5 Hz, 1H, H-4), 6.35 (t, J = 7.7 Hz, 1H, H-5), 6.08 (s, 2H, H-9), 4.63 (s, 2H, H-11).

PROTON_01

Compound 4

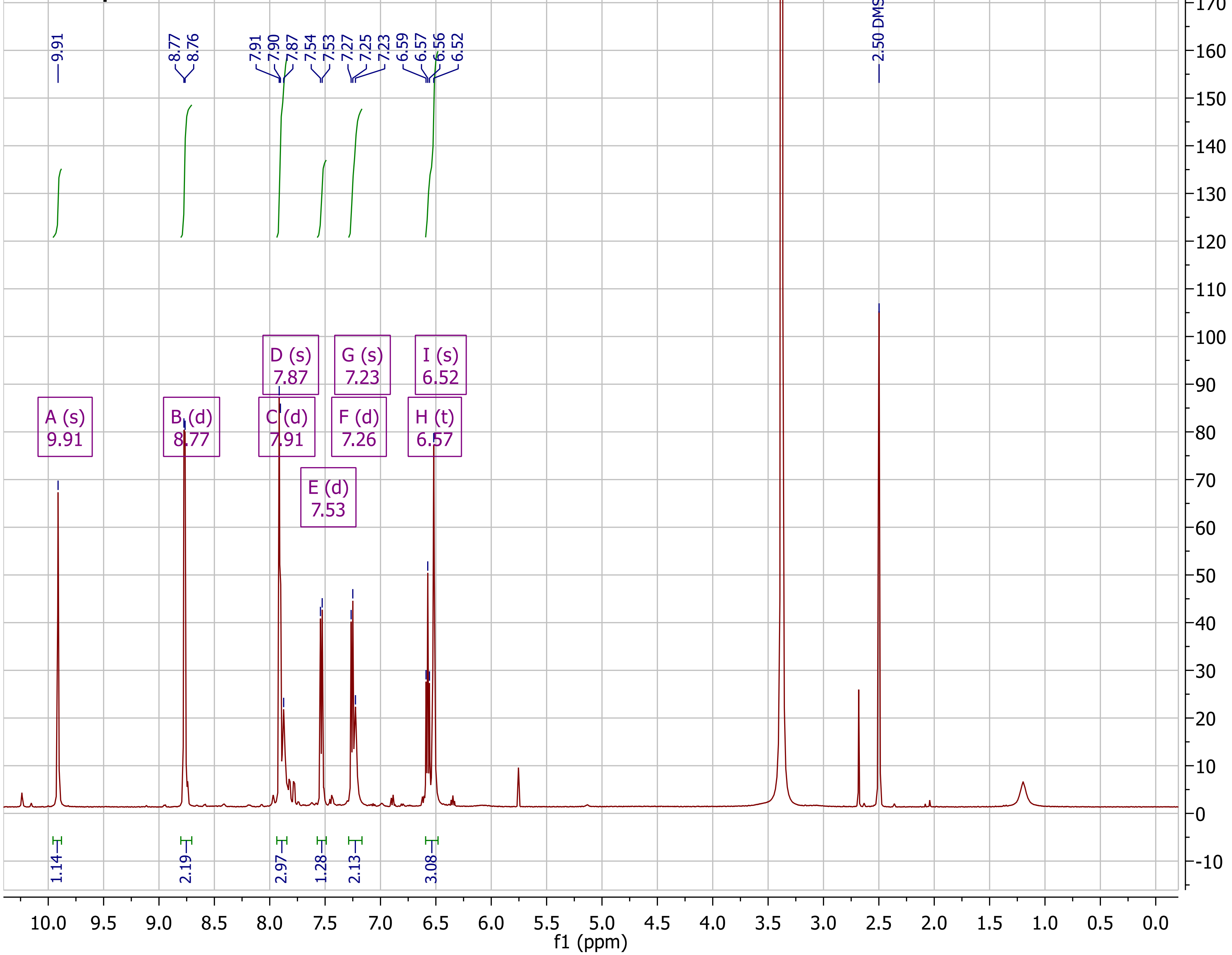

Sussex Drug Discovery Centre

	Parameter	Value
1	Data File Name	Y:/ walkup/ sew/ 20170331/ N2119-41-RMc_01/ PROTON_01.fid/ fid
2	Title	PROTON_01
3	Comment	
4	Origin	Varian
5	Owner	
6	Site	
7	Spectrometer	vnmrs
8	Author	
9	Solvent	dmso
10	Temperature	30.0
11	Pulse Sequence	s2pul
12	Experiment	1D
13	Probe	P8898_walkup
14	Number of Scans	8
15	Receiver Gain	30
16	Relaxation Delay	1.0000
17	Pulse Width	4.3000
18	Presaturation Frequency	
19	Acquisition Time	2.0447
20	Acquisition Date	2017-03-31T11:55:19
21	Modification Date	2017-03-31T11:55:48
22	Class	

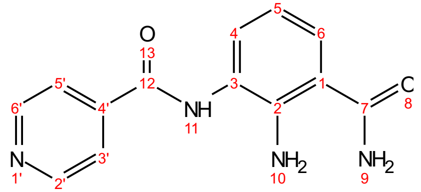

^{1}H NMR (500 MHz, DMSO-d_6) δ 9.91 (s, 1H, H-11), 8.77 (d, J = 5.3 Hz, 2H, H-2' and H-6'), 7.91 (d, J = 5.5 Hz, 2H, H-3' and H-5'), 7.87 (s, 1H, H-10), 7.53 (d, J = 7.9 Hz, 1H, H-6), 7.26 (d, J = 7.6 Hz, 1H, H-4), 7.23 (s, 1H, H-10), 6.57 (t, J = 7.8 Hz, 1H, H-5), 6.52 (s, 2H, H-9).

PROTON_01

Compound 5

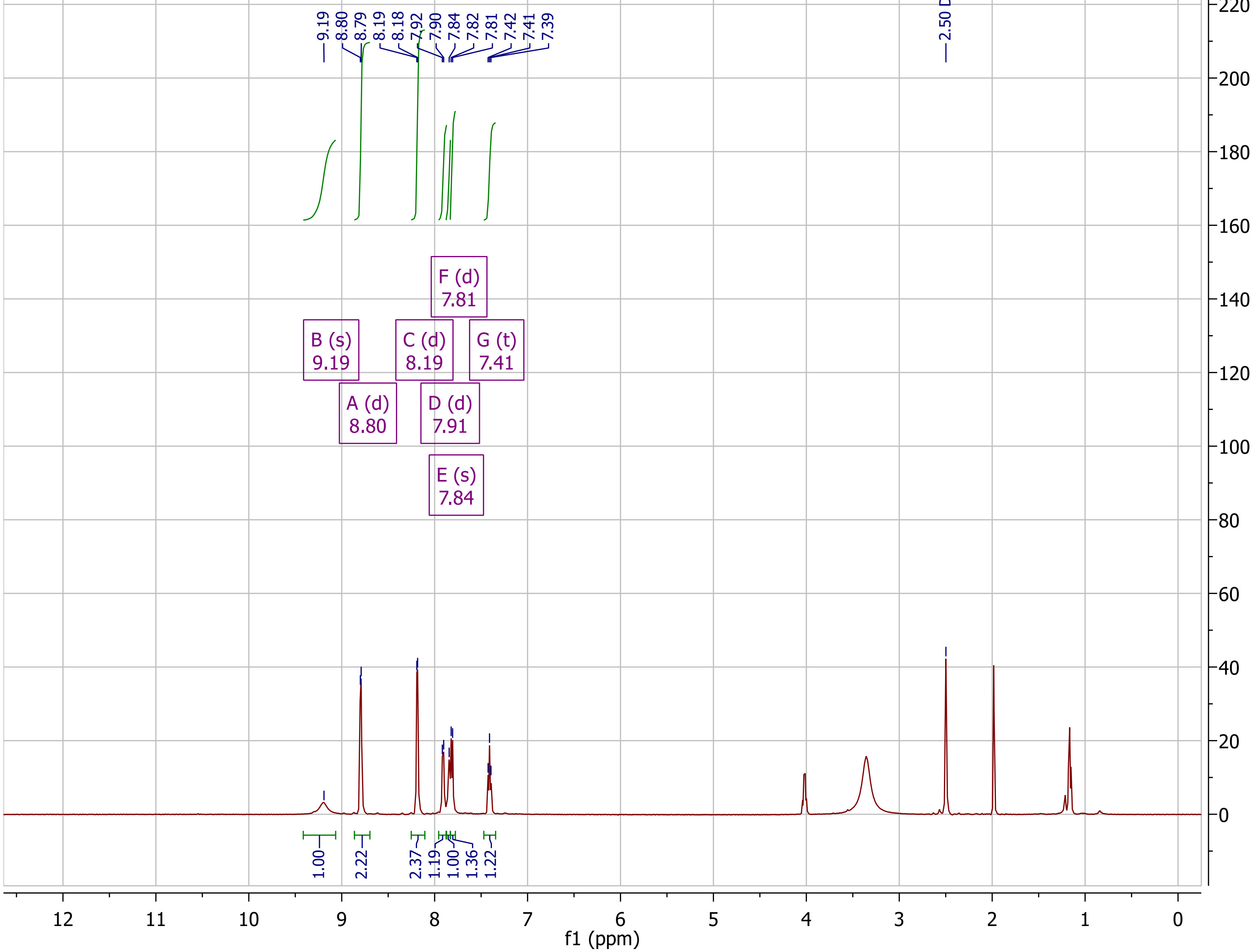

Sussex Drug Discovery Centre

	Parameter	Value
1	Data File Name	Y:/ walkup/ sew/ 20170420/ N2119-43-1_01/ PROTON_01.fid/ fid
2	Title	PROTON_01
3	Comment	
4	Origin	Varian
5	Owner	
6	Site	
7	Spectrometer	vnmrs
8	Author	
9	Solvent	dmso
10	Temperature	30.0
11	Pulse Sequence	s2pul
12	Experiment	1D
13	Probe	P8898_walkup
14	Number of Scans	8
15	Receiver Gain	30
16	Relaxation Delay	1.0000
17	Pulse Width	4.3000
18	Presaturation Frequency	
19	Acquisition Time	2.0447
20	Acquisition Date	2017-04-20T11:00:30
21	Modification Date	2017-04-20T11:01:00
22	Class	
23	Spectrometer Frequency	499.91
24	Spectral Width	8012.8

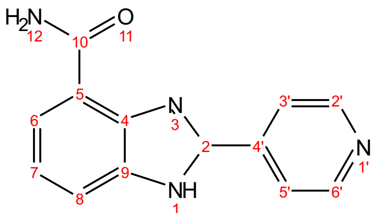

^{1}H NMR (500 MHz, DMSO-d_6) δ 9.19 (s, 2H, 2 x H-12), 8.80 (d, J = 4.3 Hz, 2H, H-2' and H-6'), 8.19 (d, J = 4.3 Hz, 2H, H-3' and H-5'), 7.91 (d, J = 7.3 Hz, 1H, H-8), 7.84 (s, 1H, H-1), 7.81 (d, J = 7.9 Hz, 1H, H-6), 7.41 (t, J = 7.7 Hz, 1H, H-7).

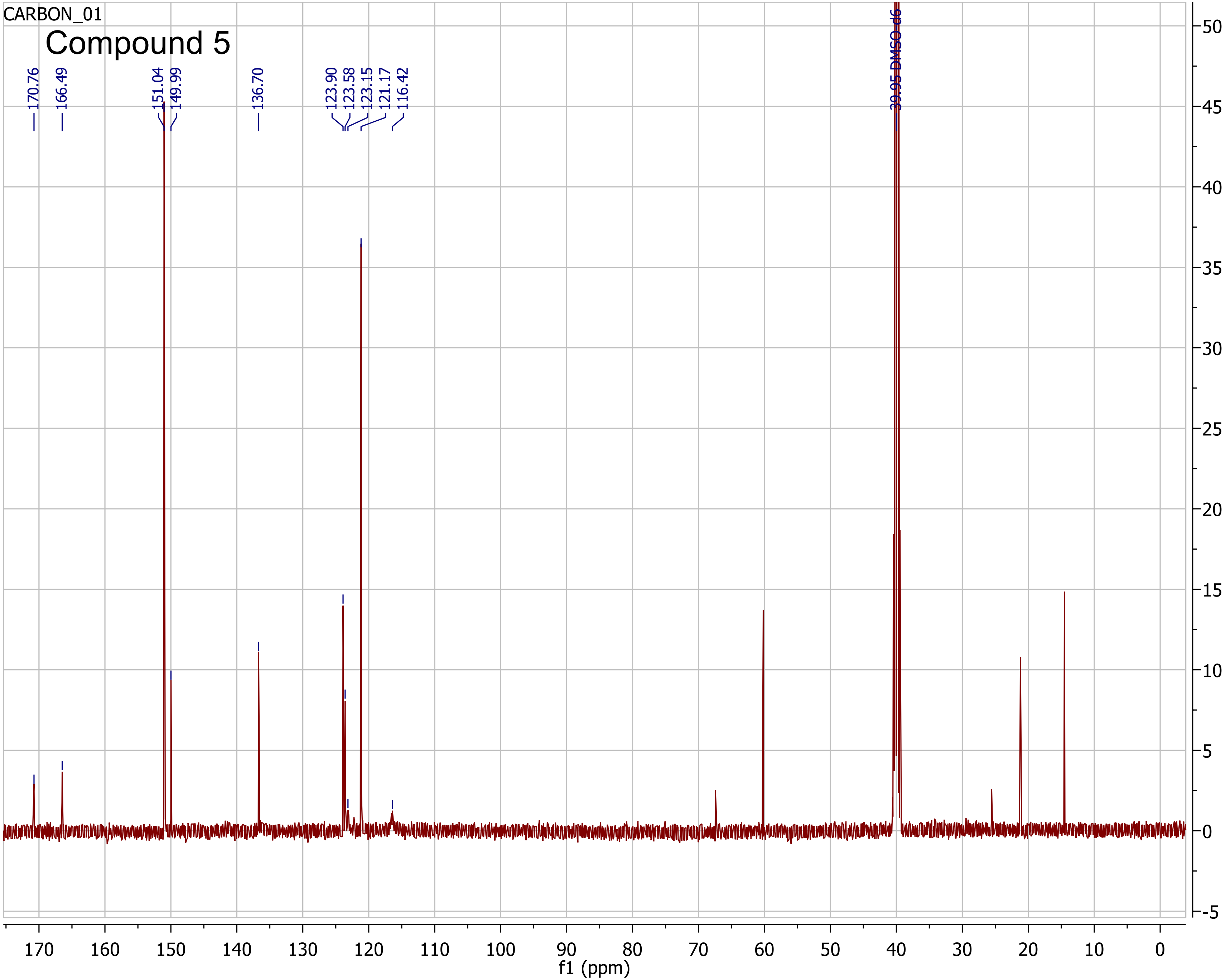

Parameter		Value
1	Data File Name	Y:/ walkup/ sew/ 20170506/ N2119-43-1_01/ CARBON_01.fid/ fid
2	Title	CARBON_01
3	Comment	
4	Origin	Varian
5	Owner	
6	Site	
7	Spectrometer	vnmrs
8	Author	
9	Solvent	dmso
10	Temperature	30.0
11	Pulse Sequence	s2pul
12	Experiment	1D
13	Probe	P8898_walkup
14	Number of Scans	10000
15	Receiver Gain	30
16	Relaxation Delay	1.0000
17	Pulse Width	5.8000
18	Presaturation Frequency	
19	Acquisition Time	1.0486
20	Acquisition Date	2017-05-06T17:39:30
21	Modification Date	2017-05-06T23:21:01
22	Class	
23	Spectrometer Frequency	125.72
24	Spectral Width	31250.0

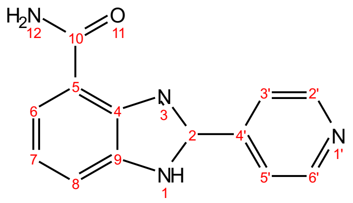

^{13}C NMR (126 MHz, dmso) δ 170.76 (C-10), 166.49 (C-5), 151.04 (C-2' and C-6'), 149.99 (C-4'), 136.70 (C-2), 123.90 (C-8), 123.58 (C-6), 123.15 (C-7), 121.17 (C3' and C-5'), 116.42 (C-9). One peak missing (C-4)

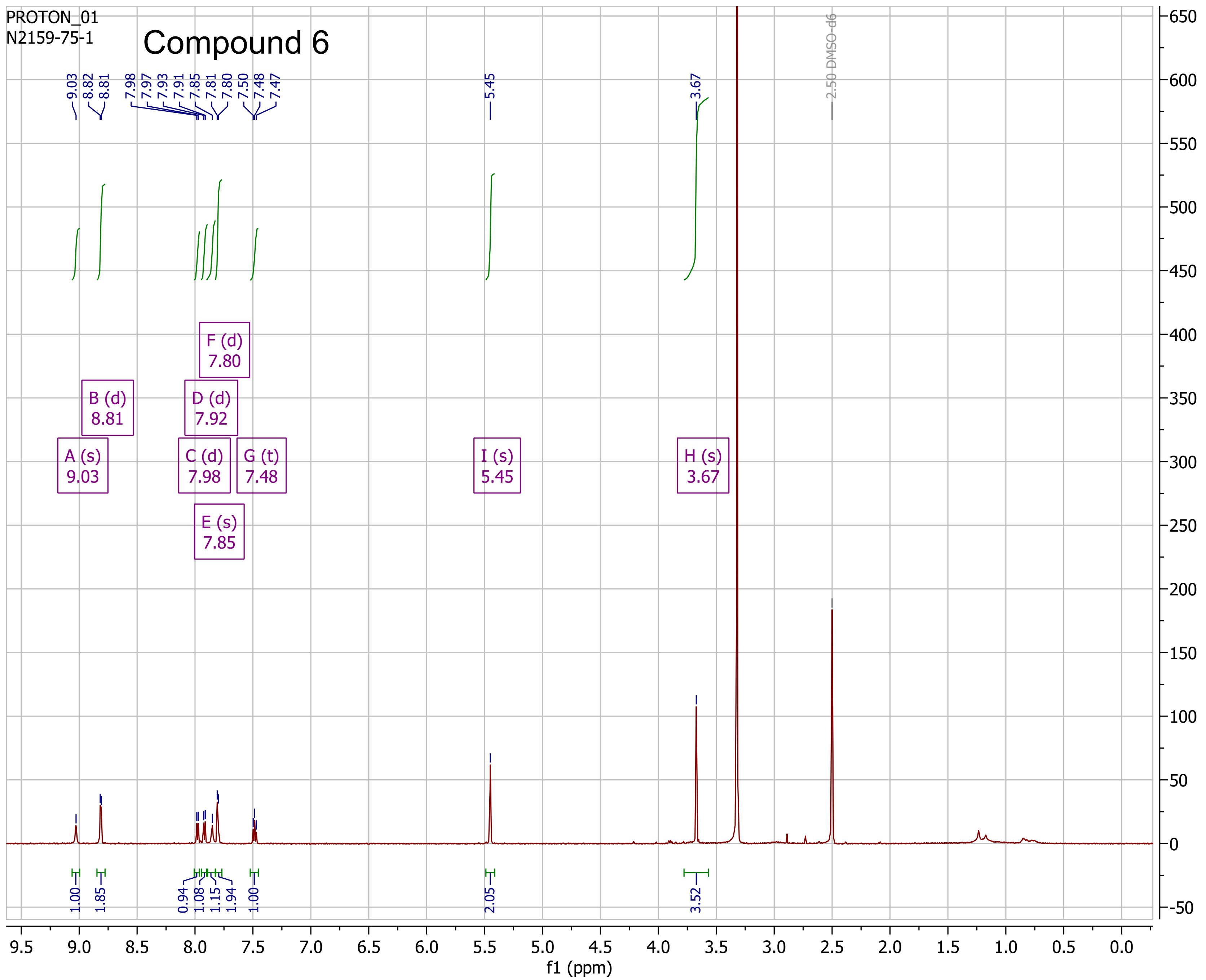

Sussex Drug Discovery Centre

	Parameter	Value
1	Data File Name	X:/ walkup/ sew/ 20180720/ N2159-75-1_01/ PROTON_01.fid/ fid
2	Title	PROTON_01
3	Comment	N2159-75-1
4	Origin	Varian
5	Owner	
6	Site	
7	Instrument	vnmrs
8	Author	
9	Solvent	dmso
10	Temperature	25.0
11	Pulse Sequence	s2pul
12	Experiment	1D
13	Probe	P8891_walkup
14	Number of Scans	8
15	Receiver Gain	50
16	Relaxation Delay	1.0000
17	Pulse Width	6.4688
18	Presaturation Frequency	
19	Acquisition Time	1.7039
20	Acquisition Date	2018-07-20T14:36:39
21	Modification Date	2018-07-20T14:37:14
22	Class	
23	Spectrometer Frequency	599.69
24	Spectral Width	9615.4

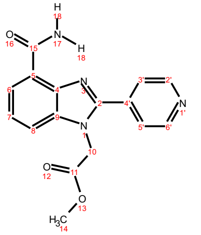

^{1}H NMR (600 MHz, DMSO-d_6) δ 9.03 (s, 1H, H-18), 8.81 (d, *J* = 5.5 Hz, 2H, H-2' and H-6'), 7.98 (d, *J* = 7.5 Hz, 1H, H-6), 7.92 (d, *J* = 8.1 Hz, 1H, H-8), 7.85 (s, 1H, H-18), 7.80 (d, *J* = 5.6 Hz, 2H, H-3' and H-5'), 7.48 (t, *J* = 7.8 Hz, 1H, H-7), 5.45 (s, 2H, H-10), 3.67 (s, 3H, H-14).

Sussex Drug Discovery Centre

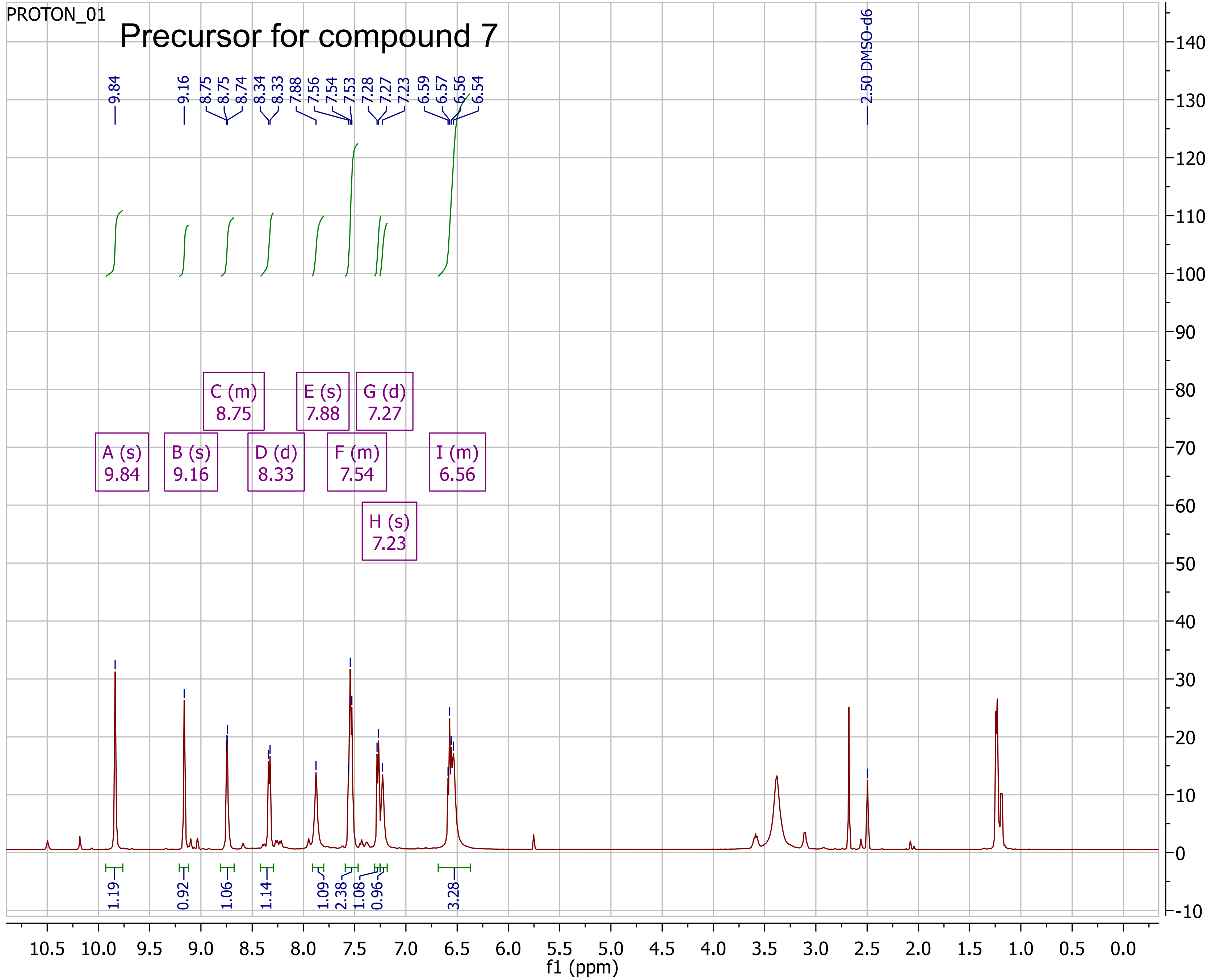

	Parameter	Value
1	Data File Name	Y:/ walkup/ sew/ 20170422/ N2119-33-1_01/ PROTON_01.fid/ fid
2	Title	PROTON_01
3	Comment	
4	Origin	Varian
5	Owner	
6	Site	
7	Spectrometer	vnmrs
8	Author	
9	Solvent	dmso
10	Temperature	30.0
11	Pulse Sequence	s2pul
12	Experiment	1D
13	Probe	P8898_walkup
14	Number of Scans	8
15	Receiver Gain	18
16	Relaxation Delay	1.0000
17	Pulse Width	4.3000
18	Presaturation Frequency	
19	Acquisition Time	2.0447
20	Acquisition Date	2017-04-22T22:02:46
21	Modification Date	2017-04-22T22:03:22
22	Class	

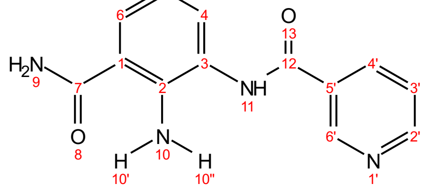

^{1}H NMR (500 MHz, DMSO-d_6) δ 9.84 (s, 1H, H-11), 9.16 (s, 1H, H-6'), 8.78 – 8.70 (m, 1H, H-2'), 8.33 (d, J = 7.8 Hz, 1H, H-4'), 7.88 (s, 1H, H-10''), 7.60 – 7.47 (m, 2H, H-6 and H-3'), 7.27 (d, J = 7.4 Hz, 1H, H-4), 7.23 (s, 1H, H-10'), 6.56 (m, 3H, H-5 and 2 x H-9).

Compound 7

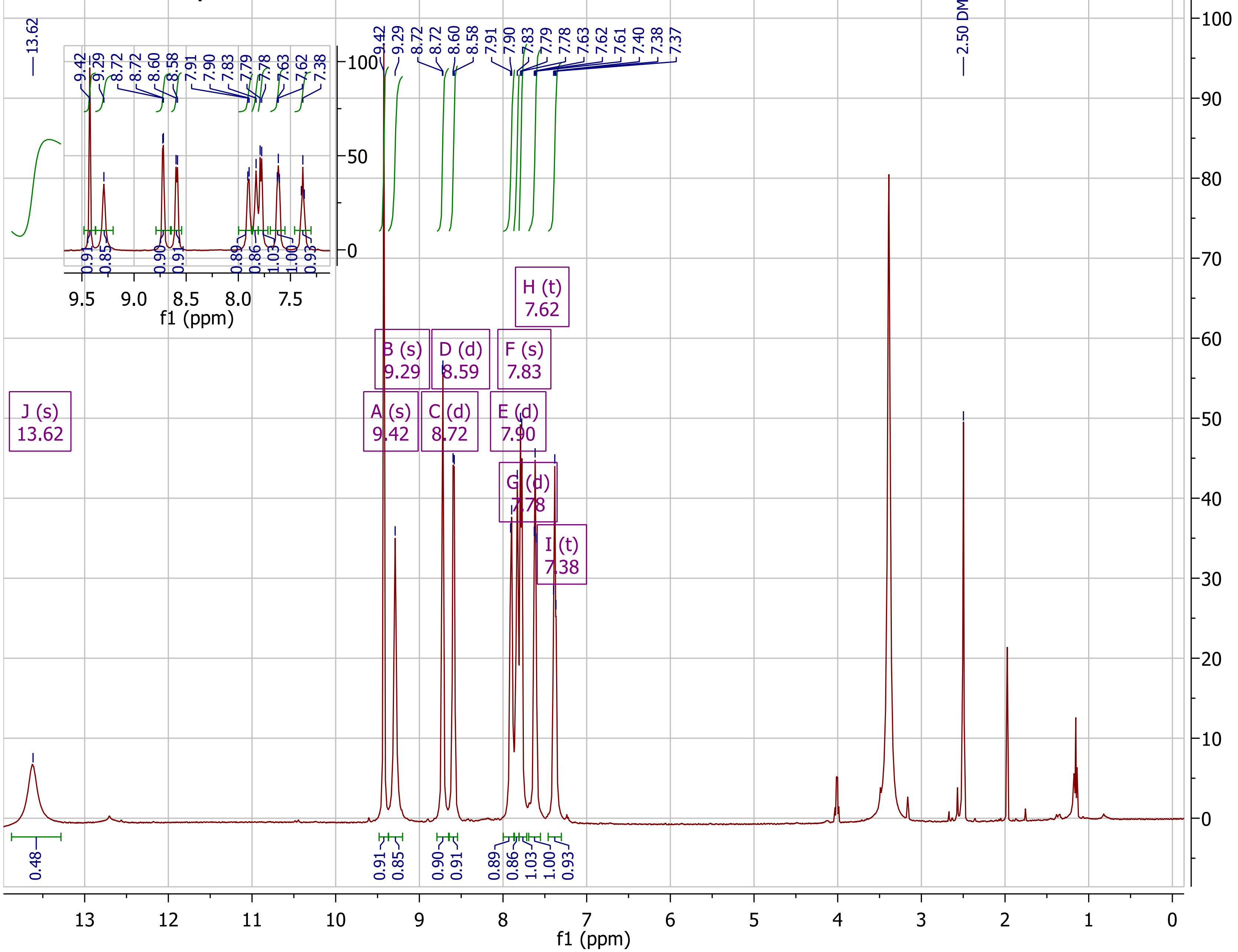

Sussex Drug Discovery Centre

	Parameter	Value
1	Data File Name	Y:/ walkup/ sew/ 20170422/ N2119-44-1_01/ PROTON_01.fid/ fid
2	Title	PROTON_01
3	Comment	
4	Origin	Varian
5	Owner	
6	Site	
7	Spectrometer	vnmrs
8	Author	
9	Solvent	dmso
10	Temperature	30.0
11	Pulse Sequence	s2pul
12	Experiment	1D
13	Probe	P8898_walkup
14	Number of Scans	8
15	Receiver Gain	30
16	Relaxation Delay	1.0000
17	Pulse Width	4.3000
18	Presaturation Frequency	
19	Acquisition Time	2.0447
20	Acquisition Date	2017-04-22T22:15:58
21	Modification Date	2017-04-22T22:16:27
22	Class	
23	Spectrometer Frequency	499.91
24	Spectral Width	8012.8

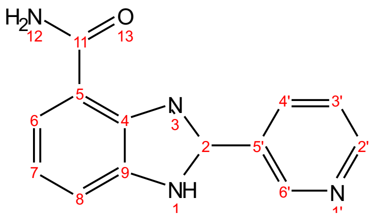

^{1}H NMR (500 MHz, DMSO-d_6) δ 13.62 (s, 1H, H-1), 9.42 (s, 1H, H-6'), 9.29 (s, 1H, H-12), 8.72 (d, 1H, H-8), 8.59 (d, *J* = 7.1 Hz, 1H, H-6), 7.94 – 7.87 (d, J = 7.90 Hz, 1H, H-2'), 7.83 (s, 1H, H-12), 7.78 (d, *J* = 7.5 Hz, 1H, H-4'), 7.62 (t, J = 5 Hz, 1H, H-7), 7.38 (t, *J* = 7.0 Hz, 1H, H-5').

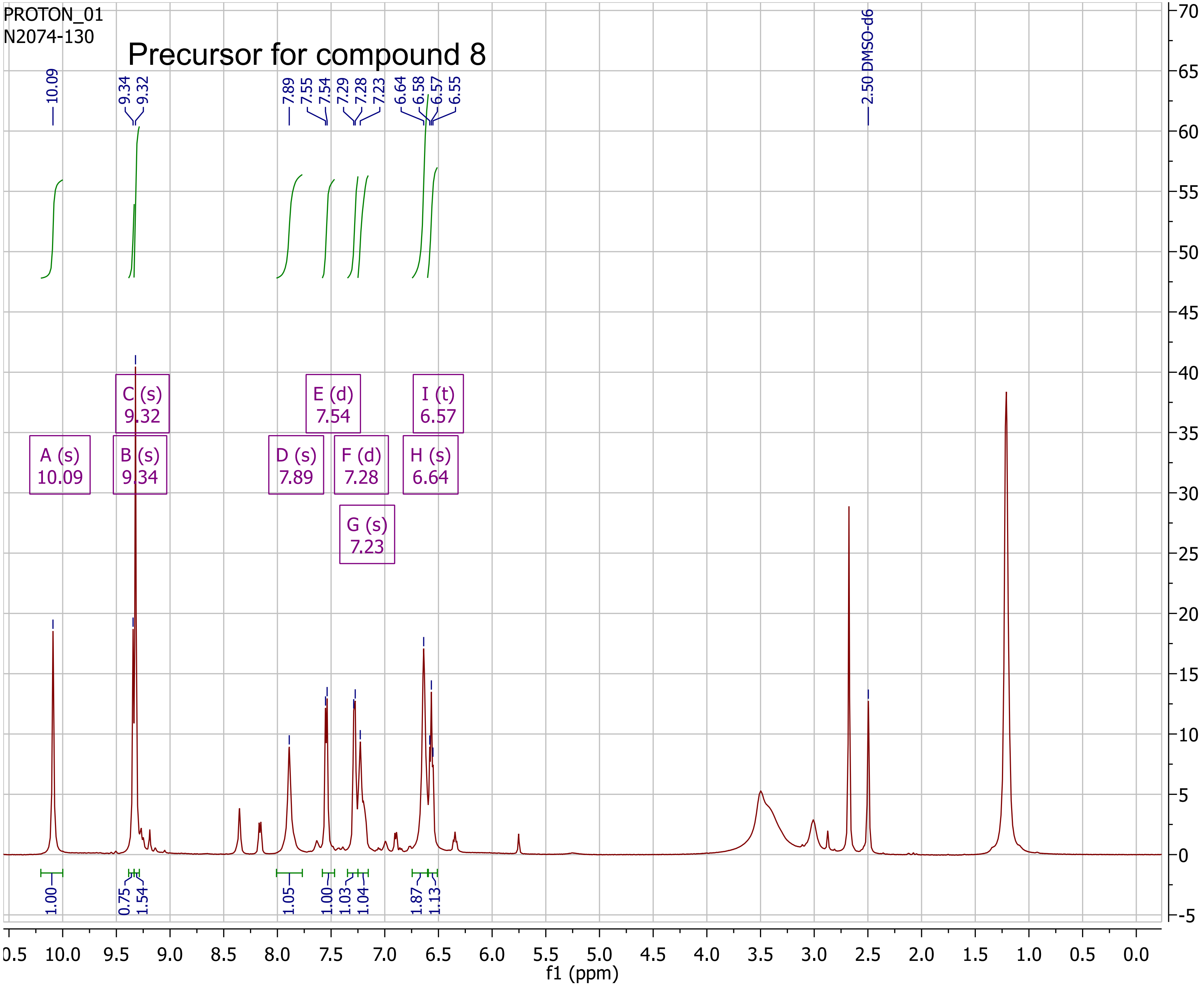

Sussex Drug Discovery Centre

	Parameter	Value
1	Data File Name	Y:/ walkup/ sew/ 20170427/ N2119-53cr_01/ PROTON_01.fid/ fid
2	Title	PROTON_01
3	Comment	N2074-130
4	Origin	Varian
5	Owner	
6	Site	
7	Spectrometer	vnmrs
8	Author	
9	Solvent	dmso
10	Temperature	30.0
11	Pulse Sequence	s2pul
12	Experiment	1D
13	Probe	P8898_walkup
14	Number of Scans	8
15	Receiver Gain	18
16	Relaxation Delay	1.0000
17	Pulse Width	4.3000
18	Presaturation Frequency	
19	Acquisition Time	2.0447
20	Acquisition Date	2017-04-27T14:35:00
21	Modification Date	2017-04-27T14:35:29
22	Class	

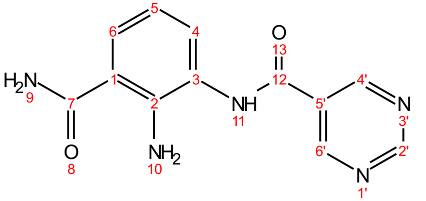

^{1}H NMR (500 MHz, DMSO-d_6) δ 10.09 (s, 1H, H-11), 9.34 (s, 1H, H-2'), 9.32 (s, 2H, H-4' and H-6'), 7.89 (s, 1H, H-10), 7.54 (d, J = 7.4 Hz, 1H, H-6), 7.28 (d, J = 7.1 Hz, 1H, H-4), 7.23 (s, 1H, H-10), 6.64 (s, 2H, 2 x H-9), 6.57 (t, J = 7.4 Hz, 1H, H-5).

PROTON_01
N2119-75-1

Compound 8

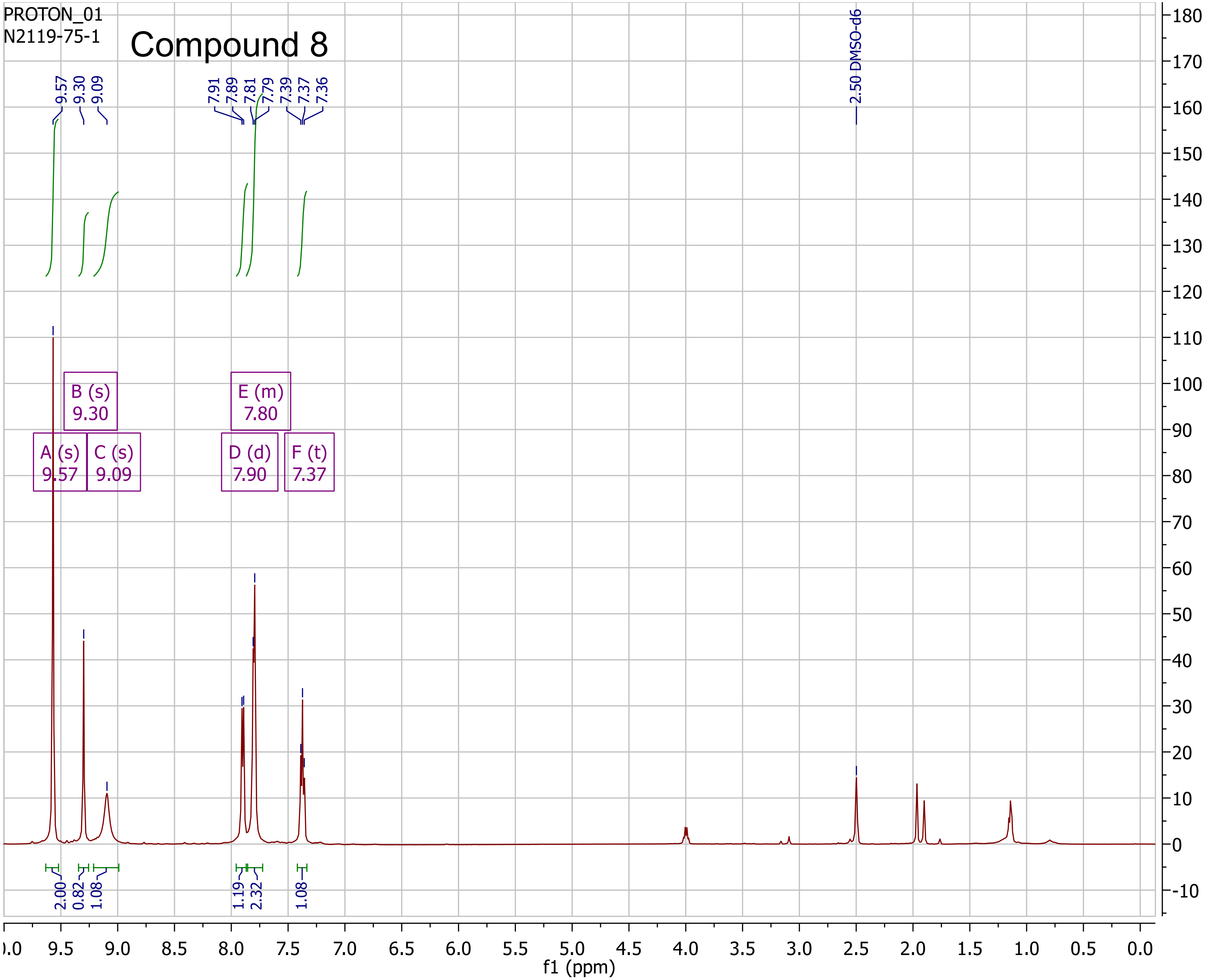

Sussex Drug Discovery Centre

	Parameter	Value
1	Data File Name	Y:/ walkup/ sew/ 20170613/ N2119-75-1_01/ PROTON_01.fid/ fid
2	Title	PROTON_01
3	Comment	N2119-75-1
4	Origin	Varian
5	Owner	
6	Site	
7	Spectrometer	vnmrs
8	Author	
9	Solvent	dmso
10	Temperature	30.0
11	Pulse Sequence	s2pul
12	Experiment	1D
13	Probe	P8898_walkup
14	Number of Scans	8
15	Receiver Gain	18
16	Relaxation Delay	1.0000
17	Pulse Width	4.3000
18	Presaturation Frequency	
19	Acquisition Time	2.0447
20	Acquisition Date	2017-06-13T14:03:50
21	Modification Date	2017-06-13T14:04:26
22	Class	
[illegible]	[illegible]	[illegible]

^{1}H NMR (500 MHz, DMSO-d_6) δ 9.57 (s, 2H, H-4' and H-6'), 9.30 (s, 1H, H-2'), 9.09 (s, 2H, H-12), 7.90 (d, *J* = 7.3 Hz, 1H, H-6), 7.84 – 7.72 (m, 2H, H-1 amd H-8), 7.37 (t, *J* = 7.7 Hz, 1H, H-7).

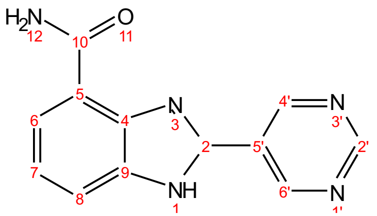

Sussex Drug Discovery Centre

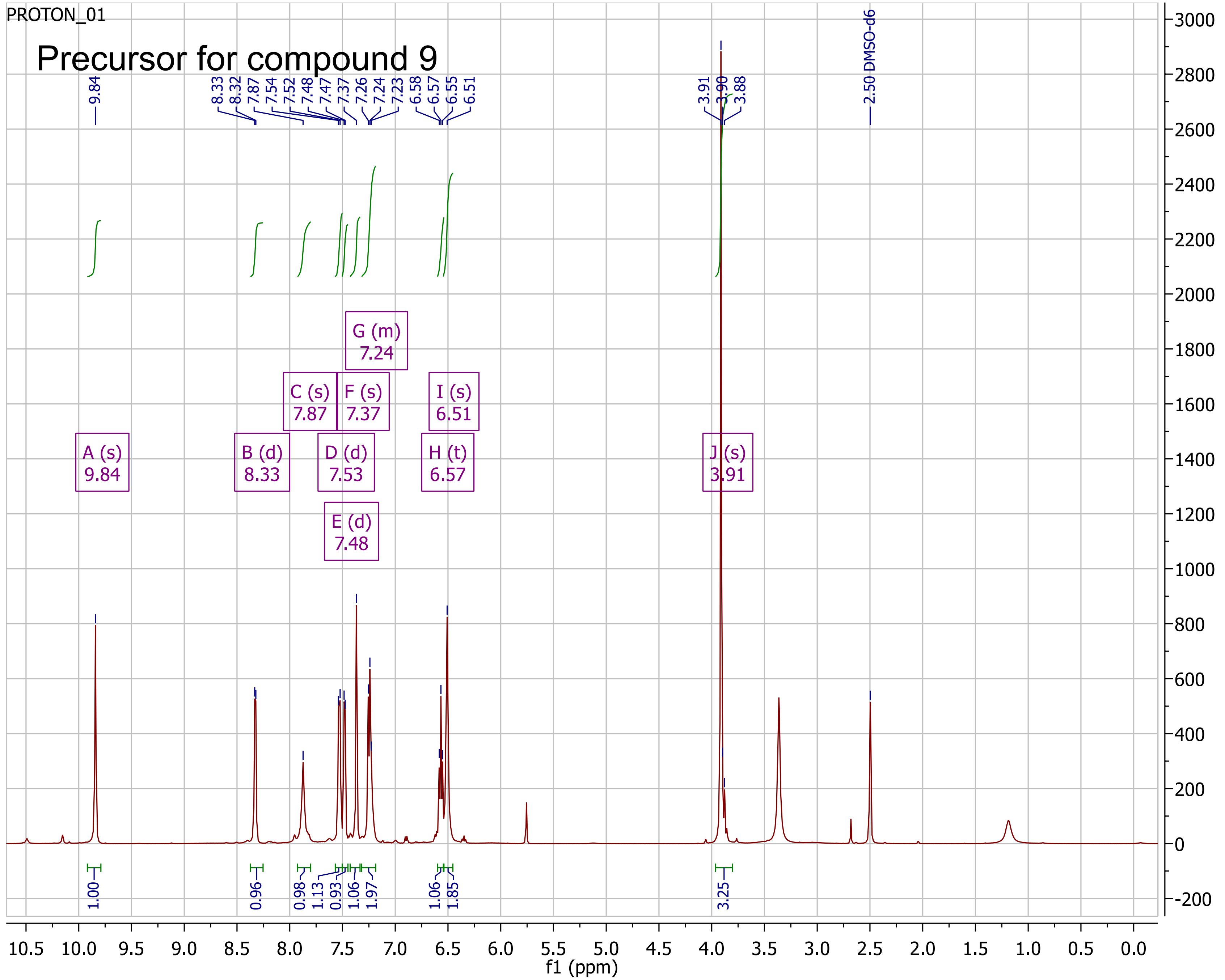

	Parameter	Value
1	Data File Name	Y:/ walkup/ sew/ 20170516/ N2119-61-cr_01/ PROTON_01.fid/ fid
2	Title	PROTON_01
3	Comment	
4	Origin	Varian
5	Owner	
6	Site	
7	Spectrometer	vnmrs
8	Author	
9	Solvent	dmso
10	Temperature	30.0
11	Pulse Sequence	s2pul
12	Experiment	1D
13	Probe	P8898_walkup
14	Number of Scans	64
15	Receiver Gain	30
16	Relaxation Delay	1.0000
17	Pulse Width	4.3000
18	Presaturation Frequency	
19	Acquisition Time	2.0447
20	Acquisition Date	2017-05-16T14:20:51
21	Modification Date	2017-05-16T14:24:12
22	Class	

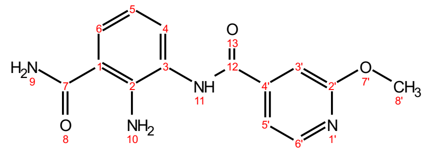

^{1}H NMR (500 MHz, DMSO-d_6) δ 9.84 (s, 1H, H-11), 8.33 (d, J = 5.3 Hz, 1H, H-6'), 7.87 (s, 1H, H-10), 7.53 (d, J = 8.0 Hz, 1H, H-4), 7.48 (d, J = 5.3 Hz, 1H, H-5), 7.37 (s, 1H, H-3'), 7.27 – 7.18 (m, 2H, H-10 ad H-6), 6.57 (t, J = 7.8 Hz, 1H, H-5), 6.51 (s, 2H, H-9), 3.91 (s, 3H, H-8').

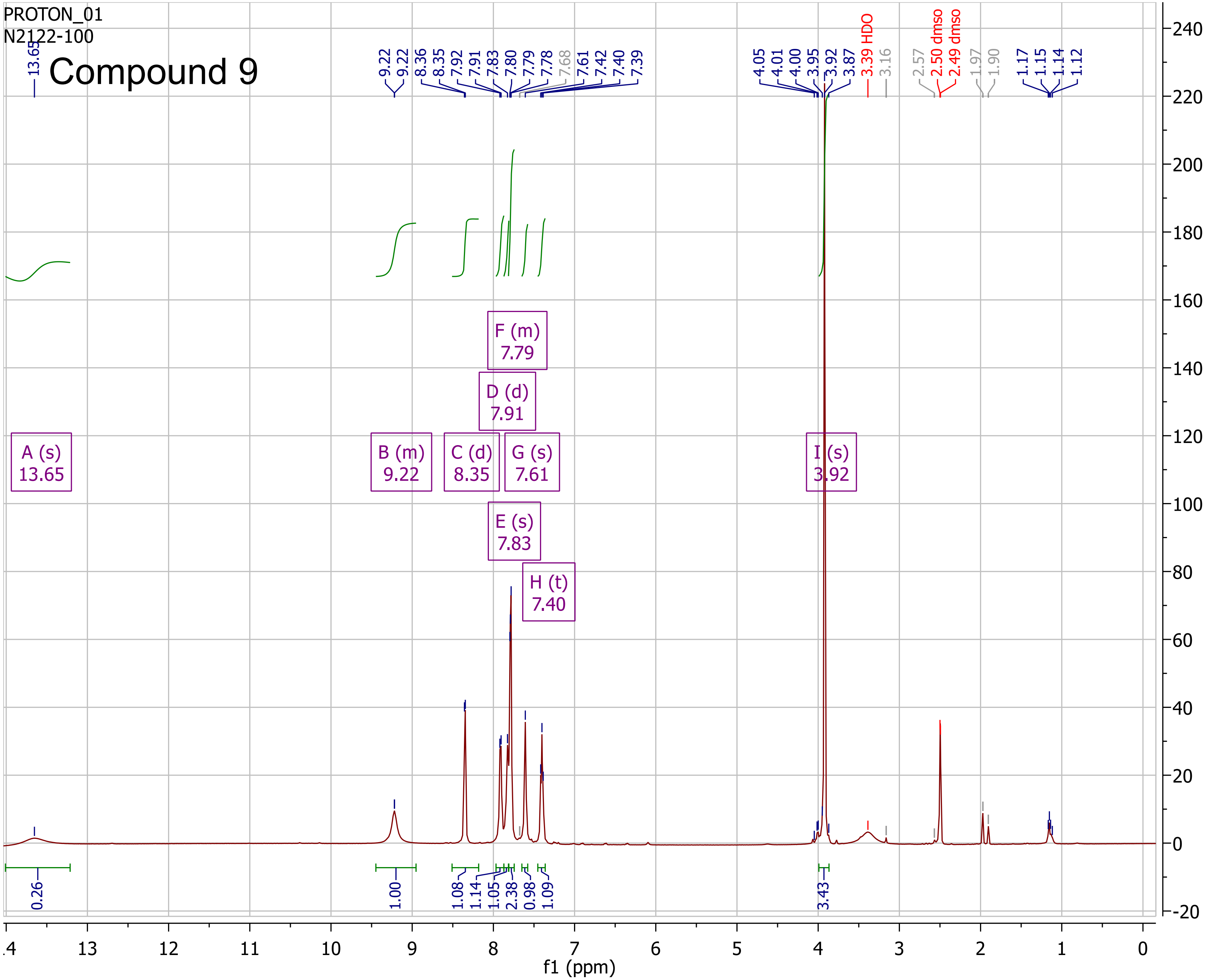

Sussex Drug Discovery Centre

	Parameter	Value
1	Data File Name	Y:/ walkup/ sew/ 20170519/ N2119-65-1_01/ PROTON_01.fid/ fid
2	Title	PROTON_01
3	Comment	N2122-100
4	Origin	Varian
5	Owner	
6	Site	
7	Spectrometer	vnmrs
8	Author	
9	Solvent	dmso
10	Temperature	30.0
11	Pulse Sequence	s2pul
12	Experiment	1D
13	Probe	P8898_walkup
14	Number of Scans	16
15	Receiver Gain	18
16	Relaxation Delay	1.0000
17	Pulse Width	4.3000
18	Presaturation Frequency	
19	Acquisition Time	2.0447
20	Acquisition Date	2017-05-19T12:35:51
21	Modification Date	2017-05-19T12:36:45
22	Class	

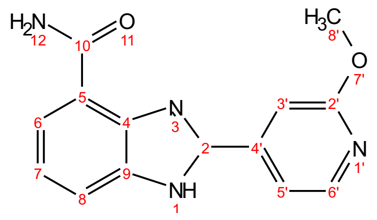

^{1}H NMR (500 MHz, DMSO-d_6) δ 13.64 (s, 1H, H-1), 9.22 (s, 1H, H-12), 8.35 (d, J = 4.7 Hz, 1H, H-6'), 7.91 (d, J = 6.7 Hz, 1H, H-6), 7.83 (s, 1H, H-1), 7.82 – 7.76 (m, 2H, H-8 and H-5'), 7.61 (s, 1H, H-3'), 7.40 (t, J = 7.3 Hz, 1H, H-7), 3.92 (s, 3H, H-8').

Sussex Drug Discovery Centre

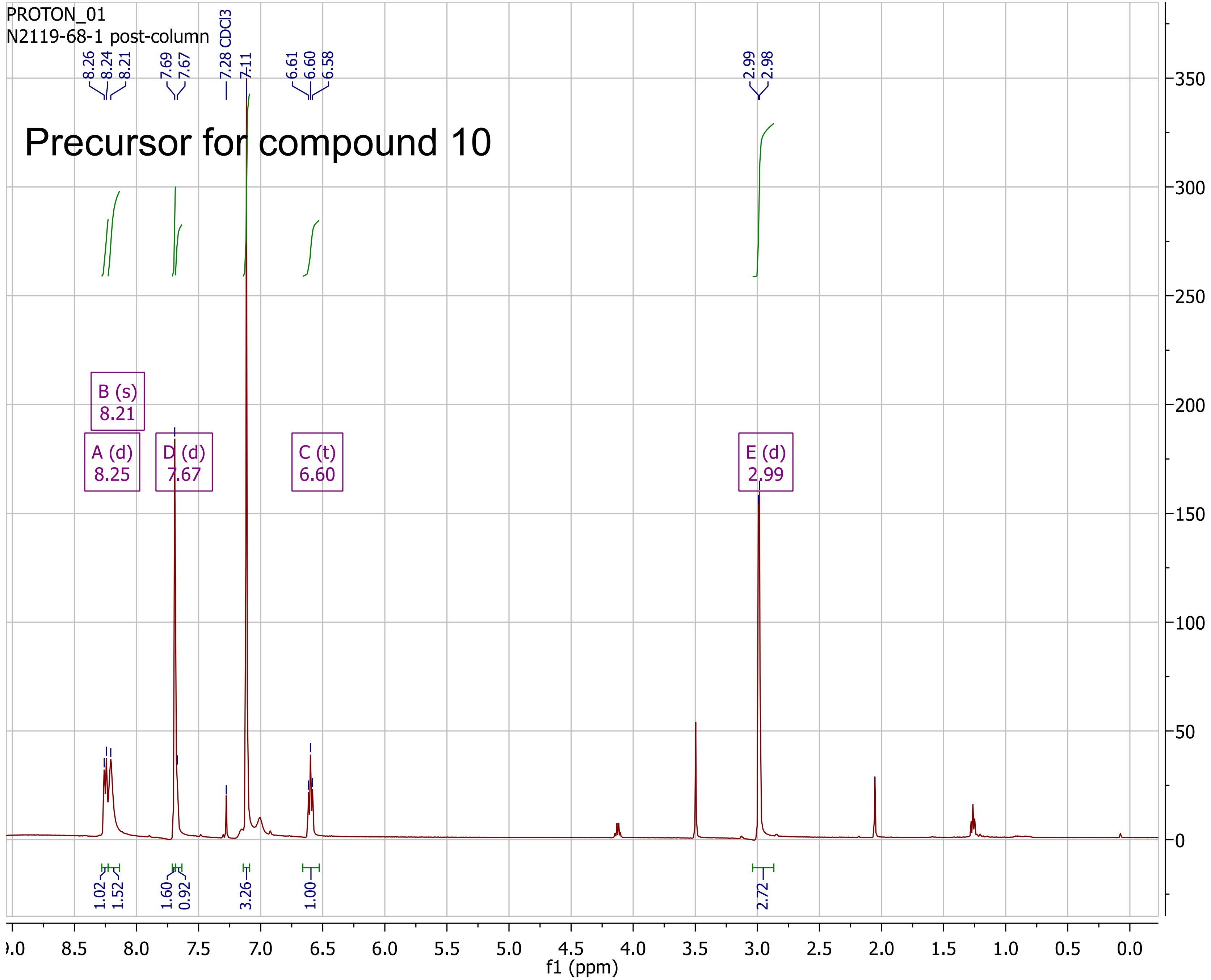

	Parameter	Value
1	Data File Name	Y:/ walkup/ sew/ 20170607/ N2119-68-1_02/ PROTON_01.fid/ fid
2	Title	PROTON_01
3	Comment	N2119-68-1 post-column
4	Origin	Varian
5	Owner	
6	Site	
7	Spectrometer	vnmrs
8	Author	
9	Solvent	cdcl3
10	Temperature	30.0
11	Pulse Sequence	s2pul
12	Experiment	1D
13	Probe	P8898_walkup
14	Number of Scans	8
15	Receiver Gain	30
16	Relaxation Delay	1.0000
17	Pulse Width	4.3000
18	Presaturation Frequency	
19	Acquisition Time	2.0447
20	Acquisition Date	2017-06-07T17:11:39
21	Modification Date	2017-06-07T17:12:08
22	Class	
23	Spectrometer Frequency	499.91
24	Spectral Width	8012.8

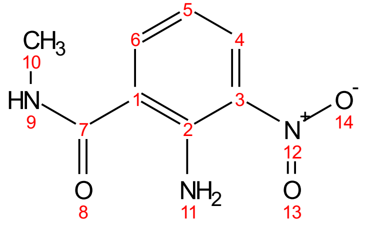

^{1}H NMR (500 MHz, Chloroform-*d*) δ 8.25 (d, *J* = 8.5 Hz, 1H, H-4), 8.21 (s, 2H, H-11), 7.67 (d, 1H, H-6), 6.60 (t, *J* = 8.0 Hz, 1H, H-5), 2.99 (d, *J* = 4.7 Hz, 3H, H-10). Peaks at δ 7.69 and 7.11 correspond to imidazole.

Sussex Drug Discovery Centre

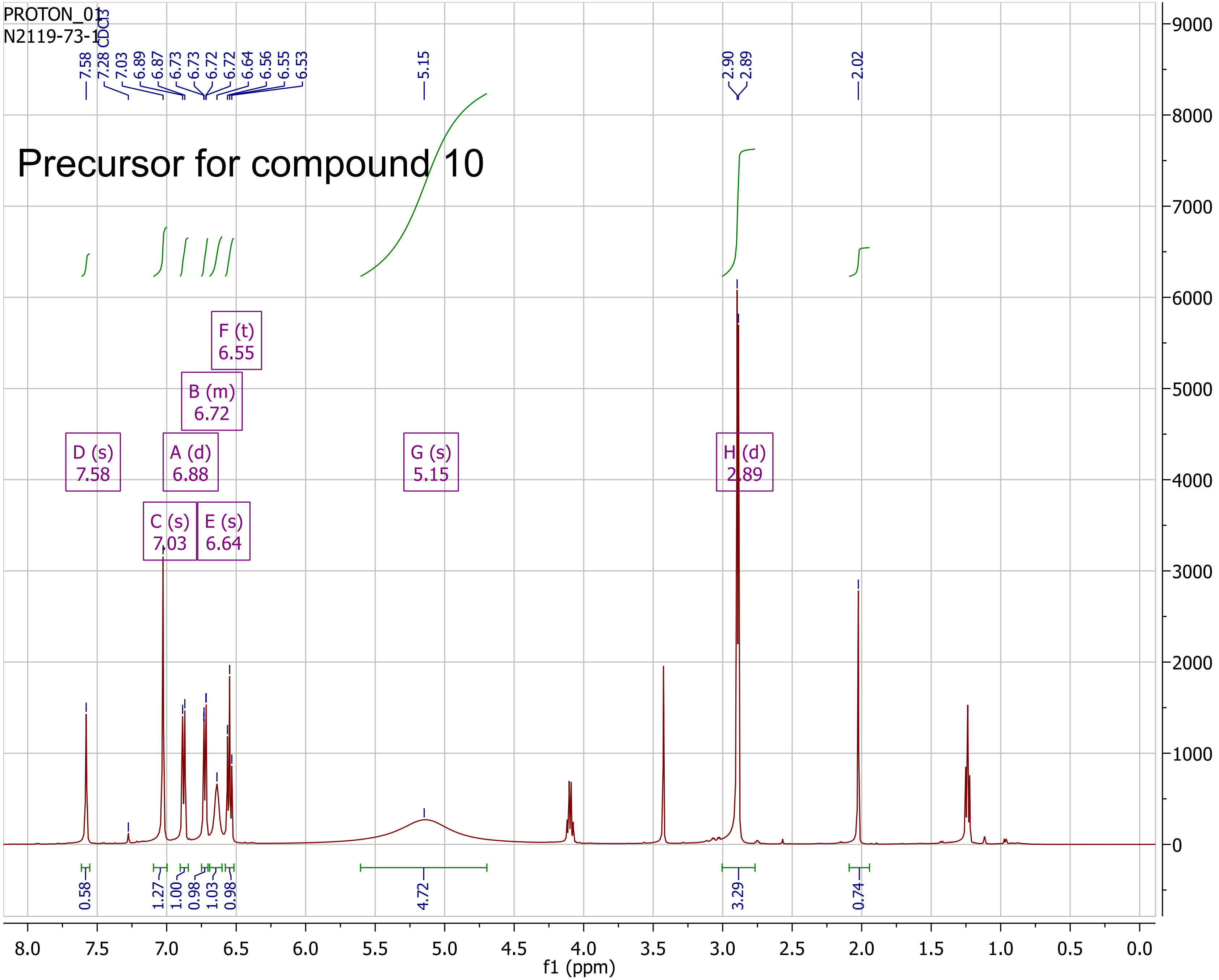

	Parameter	Value
1	Data File Name	X:/ walkup/ sew/ 20170613/ N2119-73-1_01/ PROTON_01.fid/ fid
2	Title	PROTON_01
3	Comment	N2119-73-1
4	Origin	Varian
5	Spectrometer	vnmrs
6	Solvent	cdcl3
7	Temperature	30.0
8	Pulse Sequence	s2pul
9	Experiment	1D
10	Probe	P8898_walkup
11	Number of Scans	256
12	Receiver Gain	18
13	Relaxation Delay	1.0000
14	Pulse Width	4.3000
15	Acquisition Time	2.0447
16	Acquisition Date	2017-06-13T18:19:08
17	Modification Date	2017-06-13T18:32:19
18	Spectrometer Frequency	499.91
19	Spectral Width	8012.8
20	Lowest Frequency	-996.4
21	Nucleus	1H
22	Acquired Size	16384
23	Spectral Size	65536

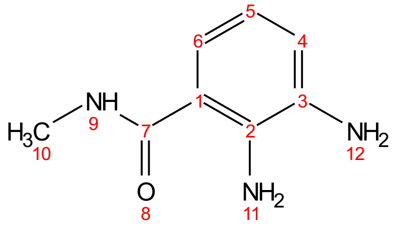

^{1}H NMR (500 MHz, Chloroform-*d*) δ 7.58 (s, 1H, H-11), 7.03 (s, 1H, H-9), 6.88 (d, *J* = 7.9 Hz, 1H, H-6), 6.77 – 6.68 (m, 1H, H-4), 6.64 (s, 1H, H-9), 6.55 (t, *J* = 7.8 Hz, 1H, H-5), 5.15 (s, 2H, H-12), 2.89 (d, *J* = 4.8 Hz, 3H, H-10).

Sussex Drug Discovery Centre

Precursor for compound 10

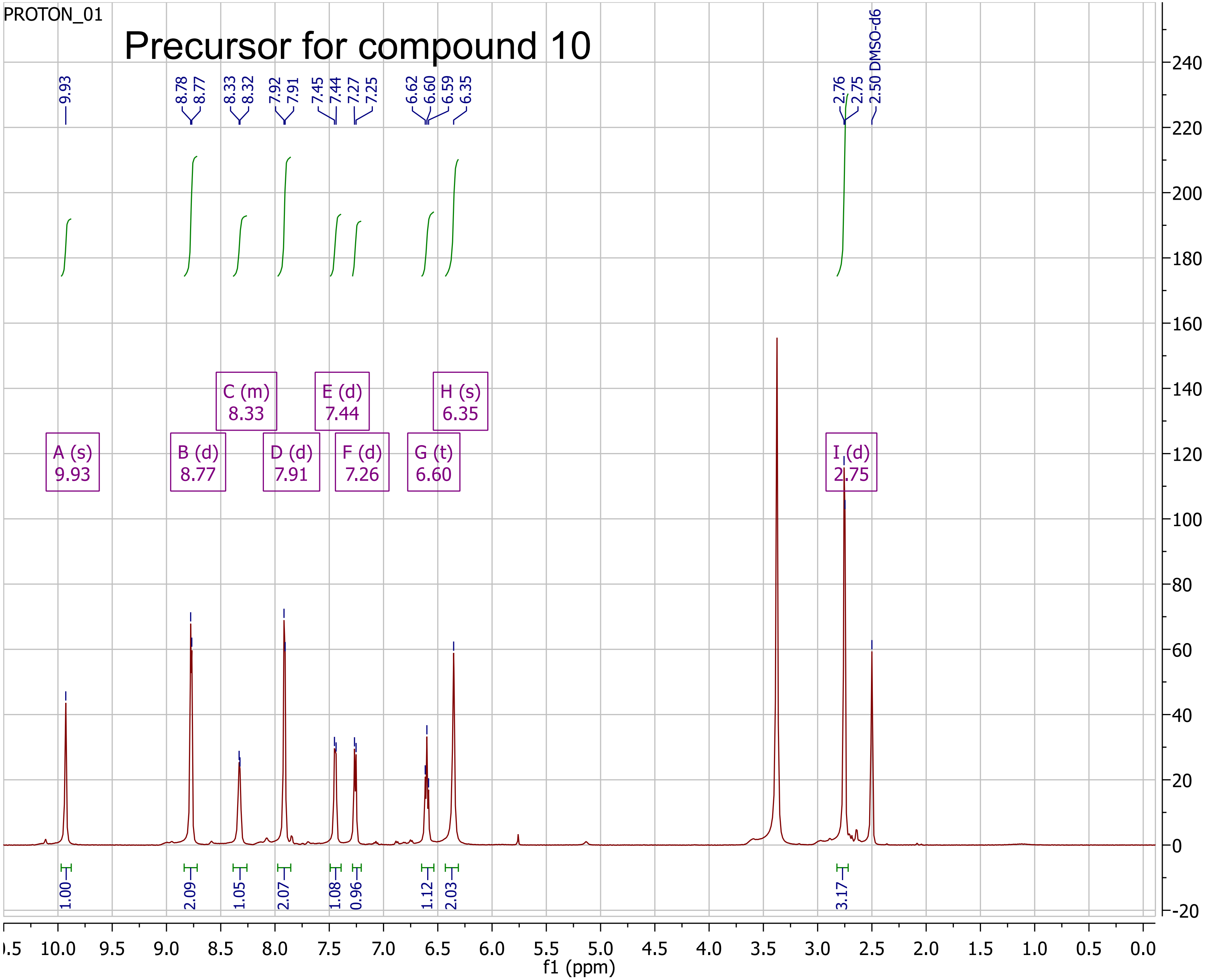

	Parameter	Value
1	Data File Name	Y:/ walkup/ sew/ 20170616/ N2119-76-cr_01/ PROTON_01.fid/ fid
2	Title	PROTON_01
3	Comment	
4	Origin	Varian
5	Owner	
6	Site	
7	Spectrometer	vnmrs
8	Author	
9	Solvent	dmso
10	Temperature	30.0
11	Pulse Sequence	s2pul
12	Experiment	1D
13	Probe	P8898_walkup
14	Number of Scans	8
15	Receiver Gain	30
16	Relaxation Delay	1.0000
17	Pulse Width	4.3000
18	Presaturation Frequency	
19	Acquisition Time	2.0447
20	Acquisition Date	2017-06-16T10:55:42
21	Modification Date	2017-06-16T10:56:19
22	Class	

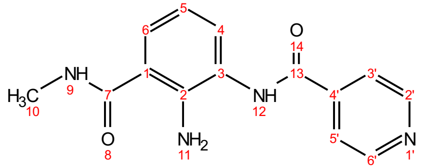

^{1}H NMR (500 MHz, DMSO-d_6) δ 9.93 (s, 1H, H-12), 8.77 (d, J = 5.2 Hz, 2H, H-2' and H-6'), 8.33 (m, 1H, H-9), 7.91 (d, J = 5.3 Hz, 2H, H-3' and H-5'), 7.44 (d, J = 7.5 Hz, 1H, H-6), 7.26 (d, J = 7.3 Hz, 1H, H-4), 6.60 (t, J = 7.6 Hz, 1H, H-5), 6.35 (s, 2H, H-11), 2.75 (d, J = 4.3 Hz, 3H, H-10).

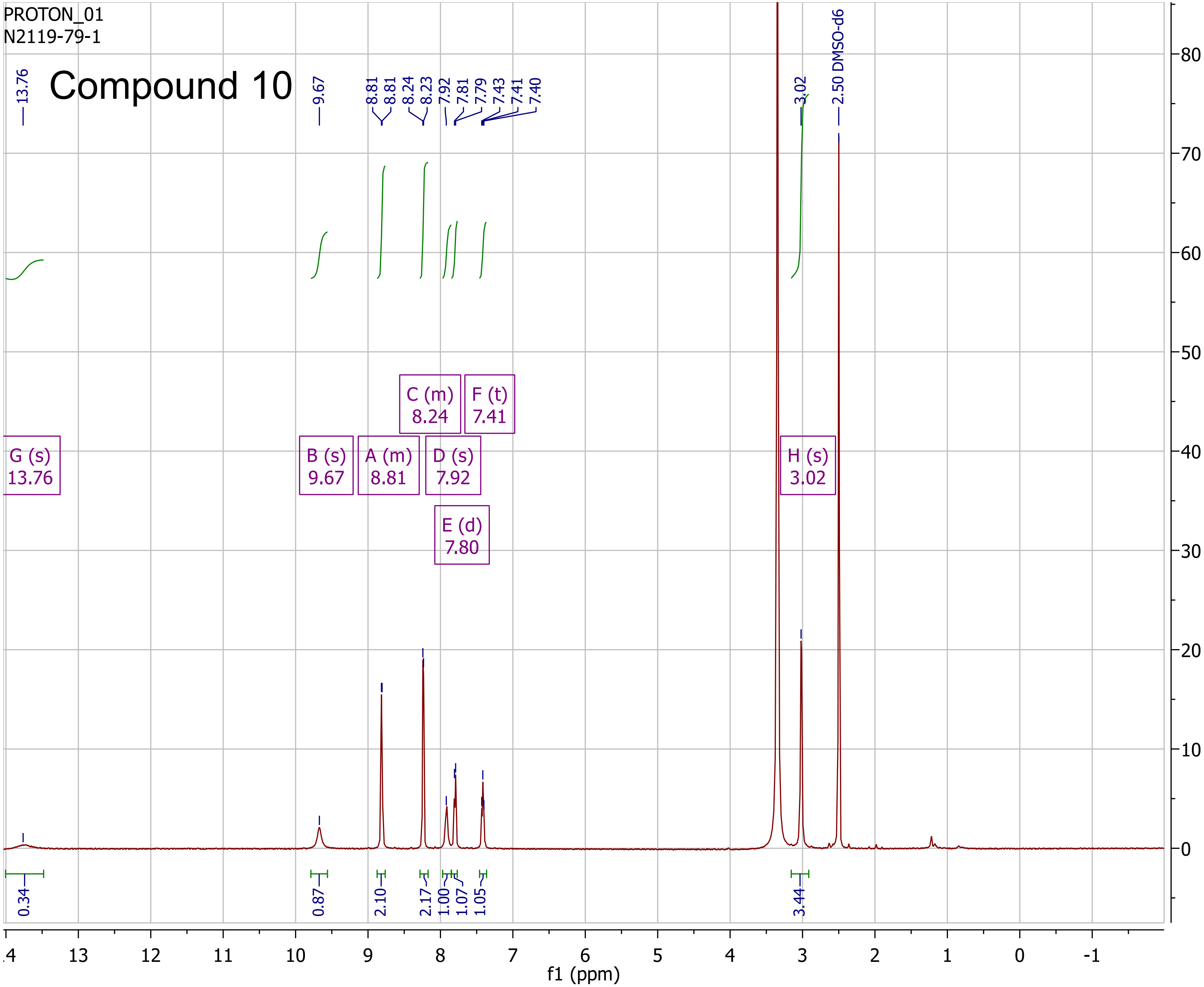

	Parameter	Value
1	Data File Name	Y:/ walkup/ sew/ 20170619/ N2119-79-1_02/ PROTON_01.fid/ fid
2	Title	PROTON_01
3	Comment	N2119-79-1
4	Origin	Varian
5	Owner	
6	Site	
7	Spectrometer	vnmrs
8	Author	
9	Solvent	dmso
10	Temperature	30.0
11	Pulse Sequence	s2pul
12	Experiment	1D
13	Probe	P8898_walkup
14	Number of Scans	8
15	Receiver Gain	30
16	Relaxation Delay	1.0000
17	Pulse Width	4.3000
18	Presaturation Frequency	
19	Acquisition Time	2.0447
20	Acquisition Date	2017-06-19T15:34:04
21	Modification Date	2017-06-19T15:34:41
22	Class	
[illegible]	[illegible]	[illegible]

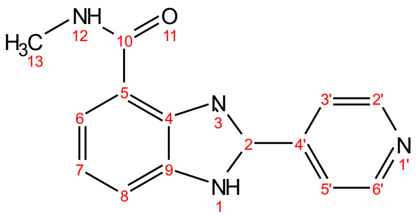

^{1}H NMR (500 MHz, DMSO-d_6) δ 13.76 (s, 1H, H-1), 9.67 (s, 1H, H-12), 8.95 – 8.70 (m, 2H, H-2' and H-6'), 8.30 – 8.17 (m, 2H, H-3' ad H-5'), 7.92 (s, 1H, H-8), 7.80 (d, *J* = 7.8 Hz, 1H, H-6), 7.41 (t, *J* = 7.5 Hz, 1H, H-7), 3.02 (s, 3H).

PROTON_01
N2119-185-1

Compound 11

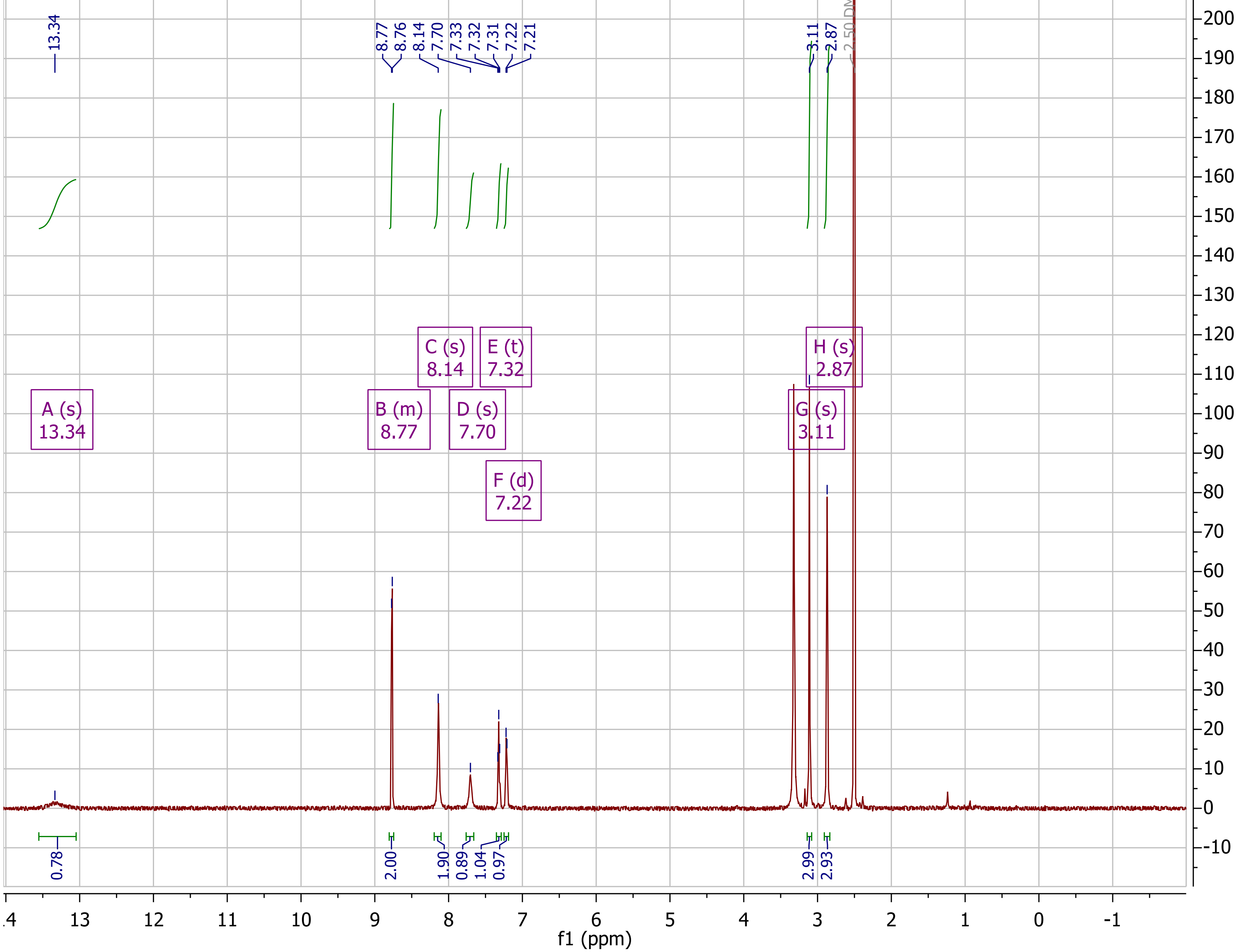

	Parameter	Value
1	Data File Name	X:/ walkup/ sew/ 20180125/ N2119-185-1_01/ PROTON_01.fid/ fid
2	Title	PROTON_01
3	Comment	N2119-185-1
4	Origin	Varian
5	Owner	
6	Site	
7	Instrument	vnmrs
8	Author	
9	Solvent	dmso
10	Temperature	25.0
11	Pulse Sequence	s2pul
12	Experiment	1D
13	Probe	P8891
14	Number of Scans	8
15	Receiver Gain	54
16	Relaxation Delay	1.0000
17	Pulse Width	5.1000
18	Presaturation Frequency	
19	Acquisition Time	1.7039
20	Acquisition Date	2018-01-25T09:20:54
21	Modification Date	2018-01-25T09:21:21
22	Class	
23	Spectrometer Frequency	599.69
24	Spectral Width	9615.4

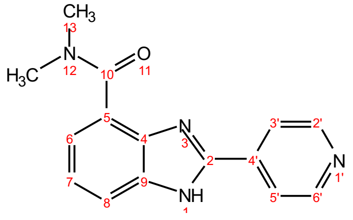

^{1}H NMR (600 MHz, DMSO-d_6) δ 13.34 (s, 1H, H-1), 8.92 – 8.59 (m, 2H, H-2' and H-6'), 8.14 (s, 2H, H-3' and H-5'), 7.70 (s, 1H, H-8), 7.32 (t, J = 7.5 Hz, 1H, H-7), 7.22 (d, J = 7.2 Hz, 1H, H-6), 3.11 (s, 3H, H-13), 2.87 (s, 3H, H-13).

PROTON_01
N2119-195-1

Precursor for compound 12

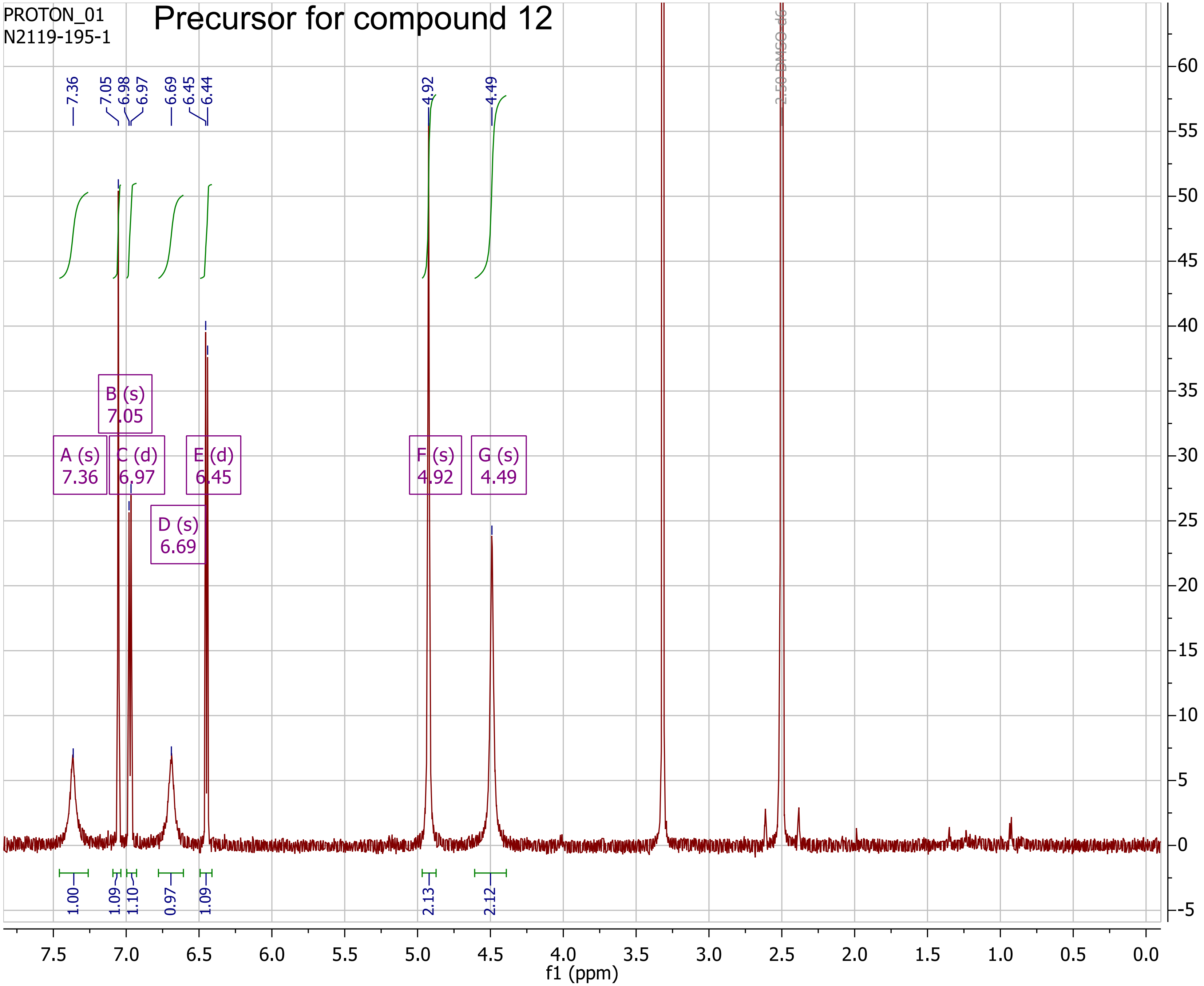

Sussex Drug Discovery Centre

	Parameter	Value
1	Data File Name	X:/ walkup/ sew/ 20180130/ N2119-195-1_01/ PROTON_01.fid/ fid
2	Title	PROTON_01
3	Comment	N2119-195-1
4	Origin	Varian
5	Owner	
6	Site	
7	Instrument	vnmrs
8	Author	
9	Solvent	dmso
10	Temperature	25.0
11	Pulse Sequence	s2pul
12	Experiment	1D
13	Probe	P8891
14	Number of Scans	8
15	Receiver Gain	54
16	Relaxation Delay	1.0000
17	Pulse Width	5.1000
18	Presaturation Frequency	
19	Acquisition Time	1.7039
20	Acquisition Date	2018-01-30T11:49:57
21	Modification Date	2018-01-30T11:50:25
22	Class	
[illegible]	[illegible]	[illegible]

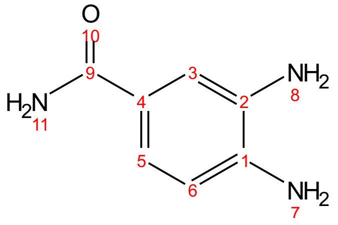

^{1}H NMR (600 MHz, DMSO-d_6) δ 7.36 (s, 1H, H-11), 7.05 (s, 1H, H-3), 6.97 (d, J = 8.0 Hz, 1H, H-5), 6.69 (s, 1H, H-11), 6.45 (d, J = 8.0 Hz, 1H, H-6), 4.92 (s, 2H, H-7), 4.49 (s, 1H, H-8).

PROTON_01
N2119-197-1

Precursor for compound 12

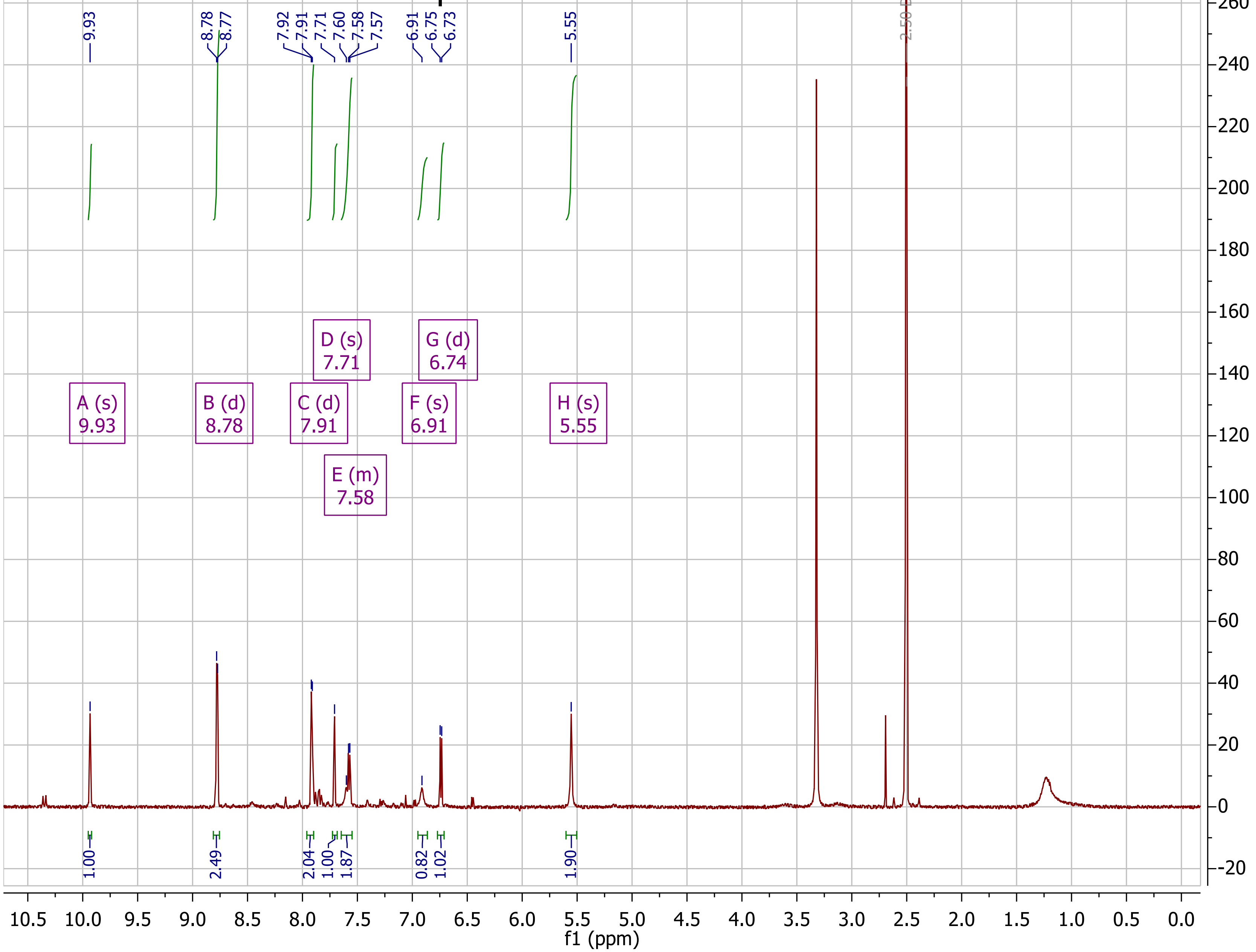

Sussex Drug Discovery Centre

	Parameter	Value
1	Data File Name	Z:/ SDDC General Access/ Personal folders/ Fiona/ Experimentals and analysis/ Experimental analysis/ N2119-197/ N2119-197-1_01/ PROTON_01.fid/ fid
2	Title	PROTON_01
3	Comment	N2119-197-1
4	Origin	Varian
5	Owner	
6	Site	
7	Instrument	vnmrs
8	Author	
9	Solvent	dmso
10	Temperature	25.0
11	Pulse Sequence	s2pul
12	Experiment	1D
13	Probe	P8891
14	Number of Scans	8
15	Receiver Gain	54
16	Relaxation Delay	1.0000
17	Pulse Width	5.1000
18	Presaturation Frequency	
19	Acquisition Time	1.7039
20	Acquisition Date	2018-01-30T15:29:51
21	Modification [illegible]	2018-01-30T15:30:18

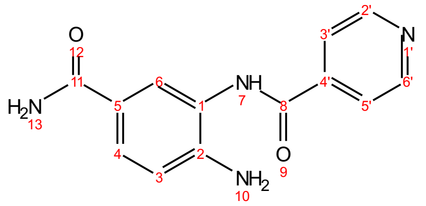

^{1}H NMR (600 MHz, DMSO-d_6) δ 9.93 (s, 1H, H-7), 8.78 (d, *J* = 5.3 Hz, 2H, H-2' and H-6'), 7.91 (d, *J* = 5.5 Hz, 2H, H-3' and H-5'), 7.71 (s, 1H, H-6), 7.66 – 7.53 (m, 2H, H-4 and H-10), 6.91 (s, 1H, H-10), 6.74 (d, *J* = 8.6 Hz, 1H, H-2), 5.55 (s, 2H, H-13).

PROTON_01
N2119-198-1

Compound 12

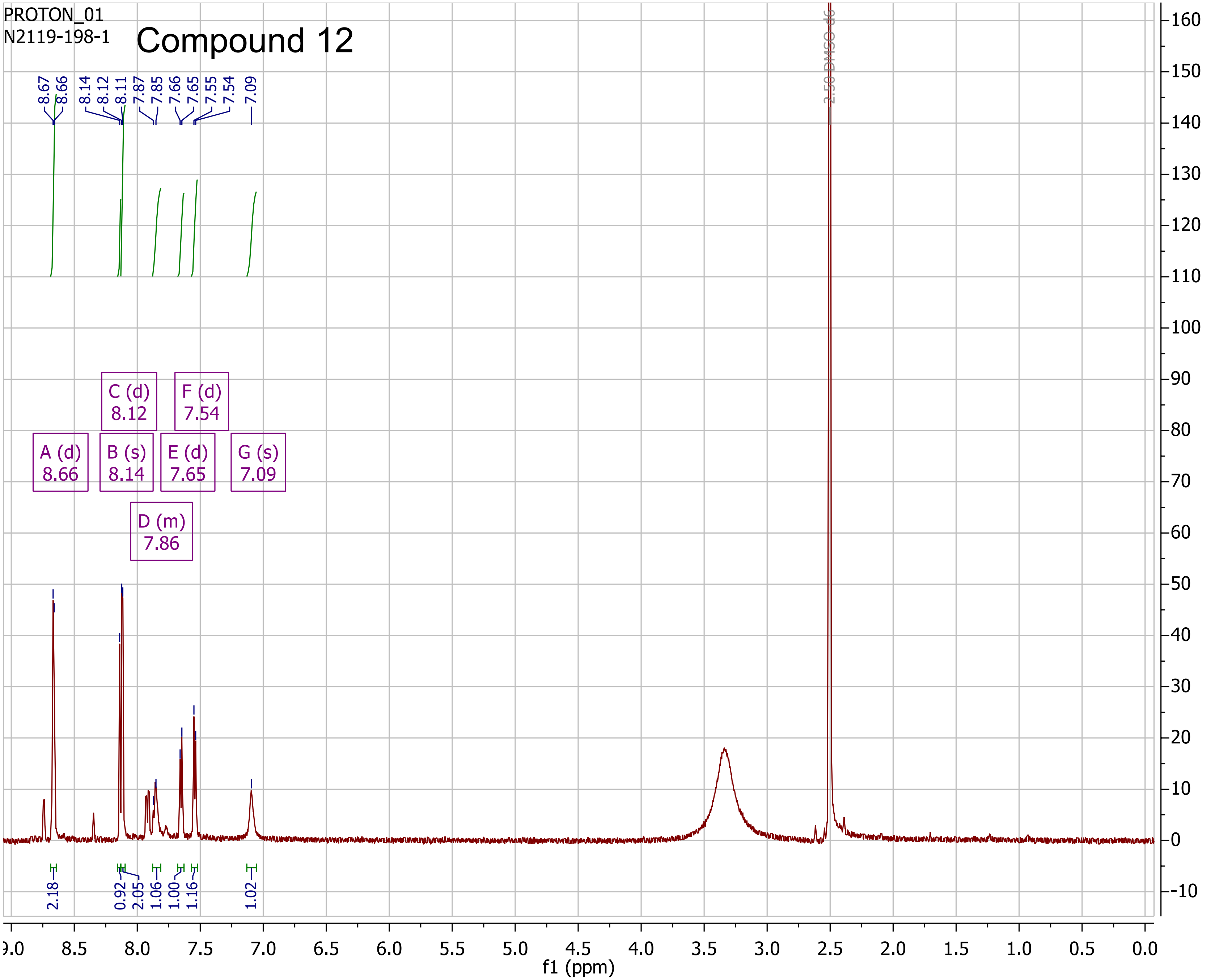

Sussex Drug Discovery Centre

	Parameter	Value
1	Data File Name	X:/ walkup/ sew/ 20180201/ N2119-198-1_01/ PROTON_01.fid/ fid
2	Title	PROTON_01
3	Comment	N2119-198-1
4	Origin	Varian
5	Owner	
6	Site	
7	Instrument	vnmrs
8	Author	
9	Solvent	dmso
10	Temperature	25.0
11	Pulse Sequence	s2pul
12	Experiment	1D
13	Probe	P8891
14	Number of Scans	8
15	Receiver Gain	54
16	Relaxation Delay	1.0000
17	Pulse Width	5.1000
18	Presaturation Frequency	
19	Acquisition Time	1.7039
20	Acquisition Date	2018-02-01T11:52:45
21	Modification Date	2018-02-01T11:53:12
22	Class	

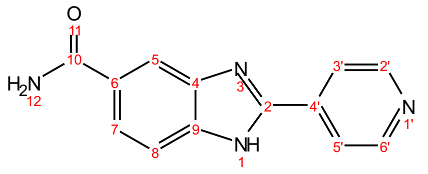

^{1}H NMR (600 MHz, DMSO-d_6) δ 8.66 (d, J = 5.1 Hz, 2H, H-2' and H-6'), 8.14 (s, 1H, H-5), 8.12 (d, J = 5.1 Hz, 2H, H-3' and H-5'), 7.89 – 7.81 (m, 1H, H-12), 7.65 (d, J = 8.4 Hz, 1H, H-7), 7.54 (d, J = 8.3 Hz, 1H, H-6), 7.09 (s, 1H, H-12).

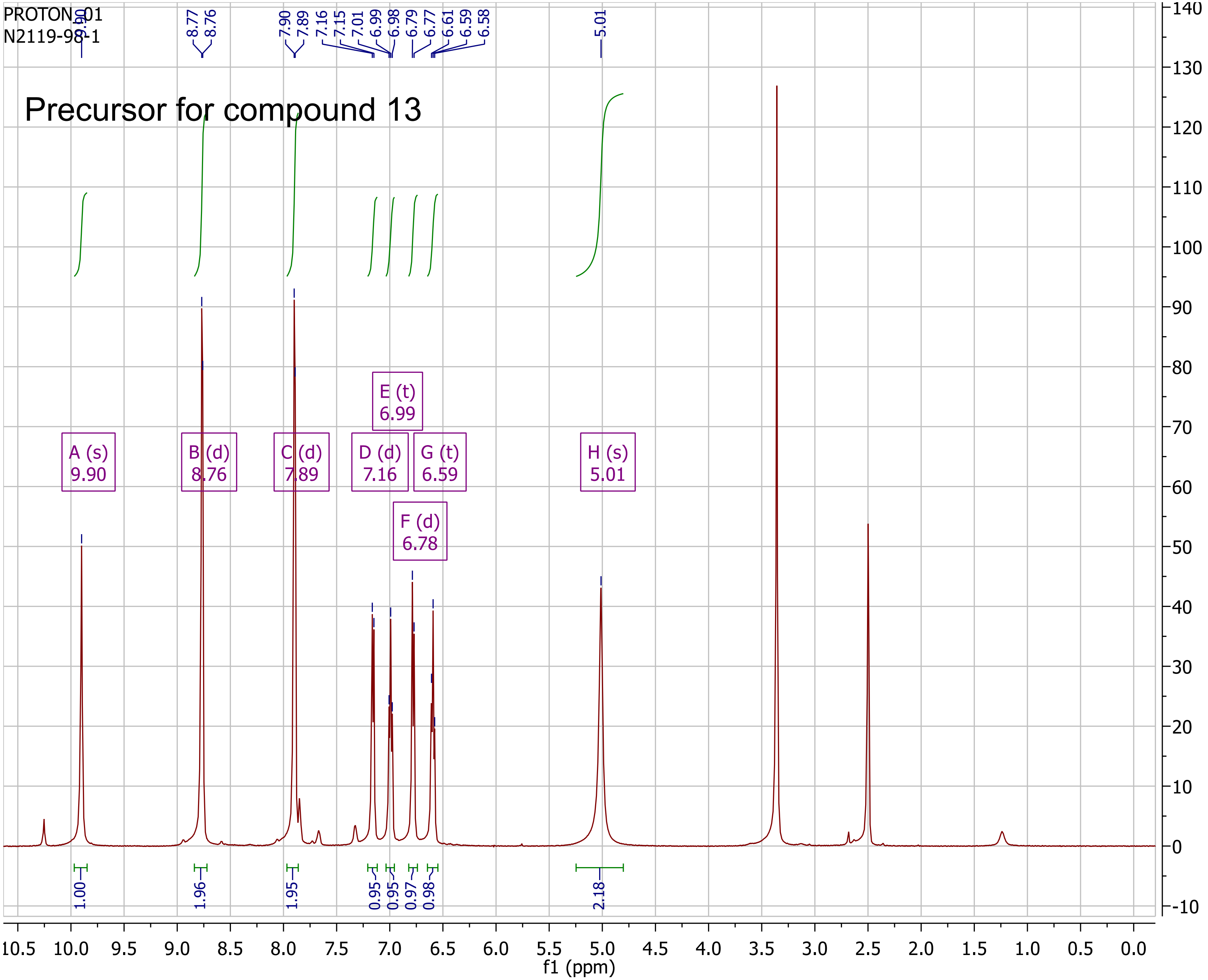

Sussex Drug Discovery Centre

	Parameter	Value
1	Data File Name	X:/ walkup/ sew/ 20170809/ N2119-98-1_01/ PROTON_01.fid/ fid
2	Title	PROTON_01
3	Comment	N2119-98-1
4	Origin	Varian
5	Spectrometer	vnmrs
6	Solvent	dmso
7	Temperature	30.0
8	Pulse Sequence	s2pul
9	Experiment	1D
10	Probe	P8898_walkup
11	Number of Scans	8
12	Receiver Gain	30
13	Relaxation Delay	1.0000
14	Pulse Width	4.3000
15	Acquisition Time	2.0447
16	Acquisition Date	2017-08-09T14:54:20
17	Modification Date	2017-08-09T14:54:56
18	Spectrometer Frequency	499.91
19	Spectral Width	8012.8
20	Lowest Frequency	-996.5
21	Nucleus	1H
22	Acquired Size	16384
23	Spectral Size	65536

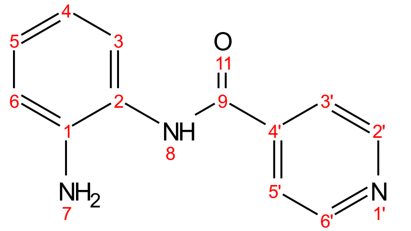

^{1}H NMR (500 MHz, DMSO-d_6) δ 9.90 (s, 1H, H-8), 8.76 (d, J = 4.6 Hz, 2H, H-2' and H-6'), 7.89 (d, J = 4.6 Hz, 2H, H-3' and H-5'), 7.16 (d, J = 7.6 Hz, 1H, H-3), 6.99 (t, J = 7.5 Hz, 1H, H-5), 6.78 (d, J = 7.9 Hz, 1H, H-6), 6.59 (t, J = 7.4 Hz, 1H, H-4), 5.01 (s, 2H, H-7).

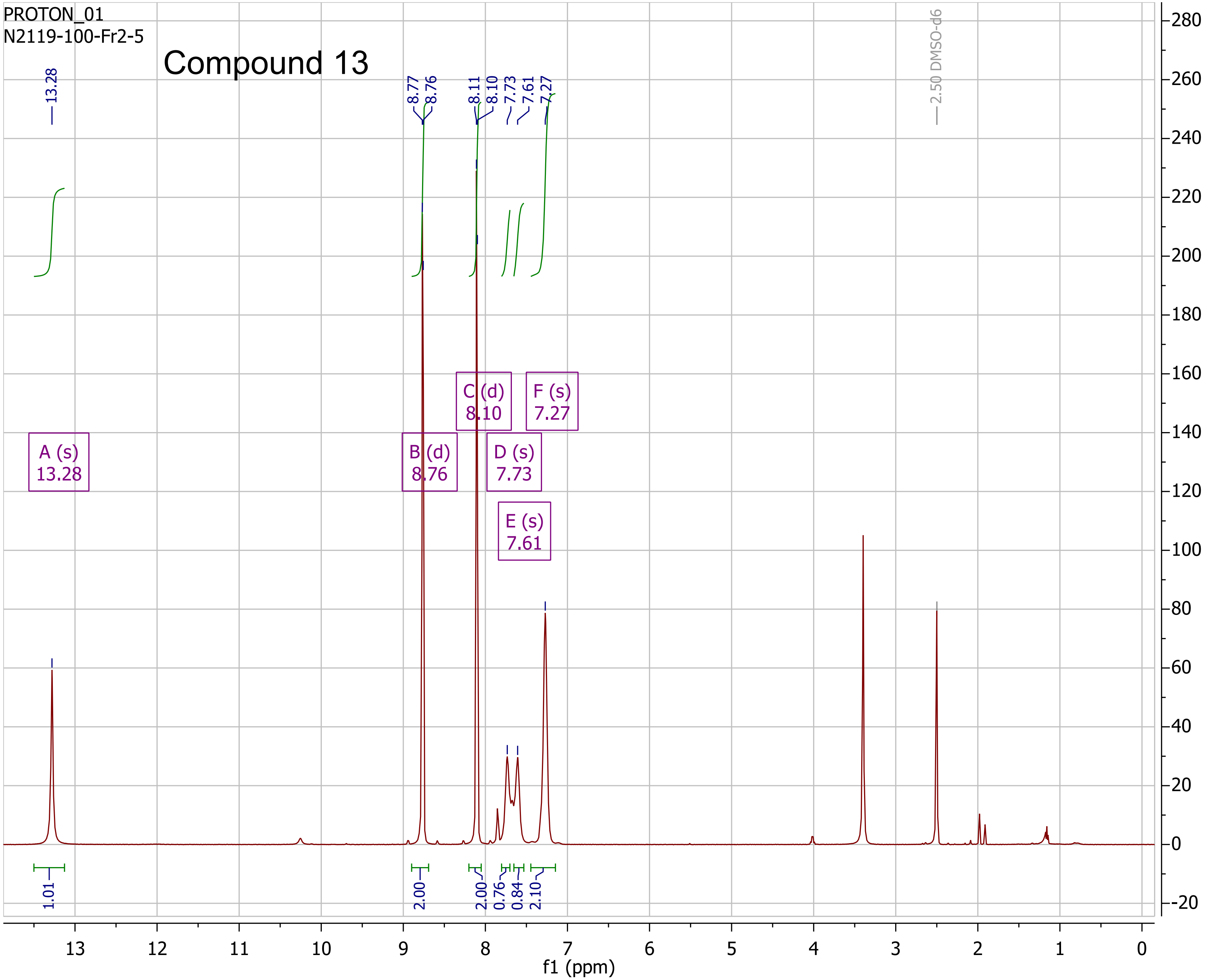

Sussex Drug Discovery Centre

	Parameter	Value
1	Data File Name	Y:/ walkup/ sew/ 20170811/ N2119-100-Fr2-5_01/ PROTON_01.fid/ fid
2	Title	PROTON_01
3	Comment	N2119-100-Fr2-5
4	Origin	Varian
5	Owner	
6	Site	
7	Spectrometer	vnmrs
8	Author	
9	Solvent	dmso
10	Temperature	30.0
11	Pulse Sequence	s2pul
12	Experiment	1D
13	Probe	P8898_walkup
14	Number of Scans	8
15	Receiver Gain	30
16	Relaxation Delay	1.0000
17	Pulse Width	4.3000
18	Presaturation Frequency	
19	Acquisition Time	2.0447
20	Acquisition Date	2017-08-11T12:25:26
21	Modification Date	2017-08-11T12:26:03
22	Class	

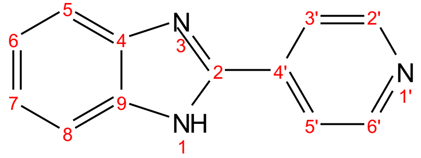

^{1}H NMR (500 MHz, DMSO-d_6) δ 13.28 (s, 1H, H-1), 8.76 (d, J = 5.7 Hz, 2H, H-2' and H-6'), 8.10 (d, J = 5.8 Hz, 2H, H-3' and H-5'), 7.73 (s, 1H, H-5), 7.61 (s, 1H, H-8), 7.27 (s, 2H, H-6 and H-7).

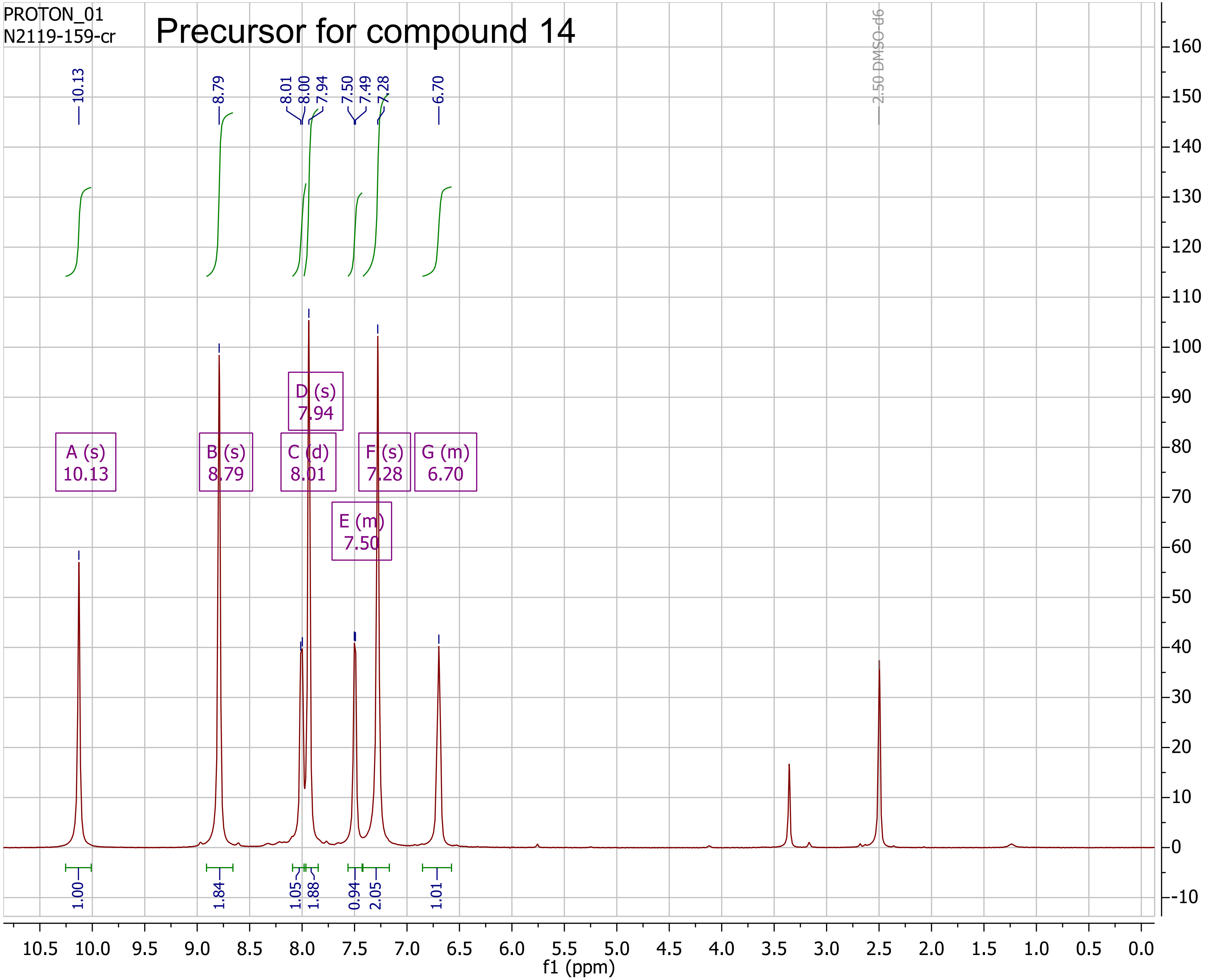

Sussex Drug Discovery Centre

	Parameter	Value
1	Data File Name	Y:/ walkup/ sew/ 20171107/ N2119-159-cr_01/ PROTON_01.fid/ fid
2	Title	PROTON_01
3	Comment	N2119-159-cr
4	Origin	Varian
5	Owner	
6	Site	
7	Instrument	vnmrs
8	Author	
9	Solvent	dmso
10	Temperature	30.0
11	Pulse Sequence	s2pul
12	Experiment	1D
13	Probe	P8898_walkup
14	Number of Scans	8
15	Receiver Gain	30
16	Relaxation Delay	1.0000
17	Pulse Width	4.3000
18	Presaturation Frequency	
19	Acquisition Time	2.0447
20	Acquisition Date	2017-11-07T11:58:21
21	Modification Date	2017-11-07T11:58:58
22	Class	
23	Spectrometer Frequency	499.91
24	Spectral Width	8012.8

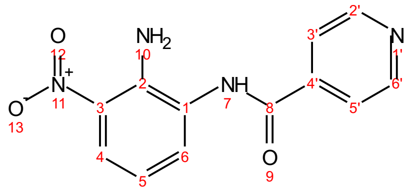

^{1}H NMR (500 MHz, DMSO-d_6) δ 10.13 (s, 1H, H-7), 8.79 (s, 2H, H-2' and H-6'), 8.01 (d, J = 7.8 Hz, 1H, H-4), 7.94 (s, 2H, H-3' and H-5'), 7.54 – 7.43 (m, 1H, H-6), 7.28 (s, 2H, H-10), 6.75 – 6.63 (m, 1H, H-5).

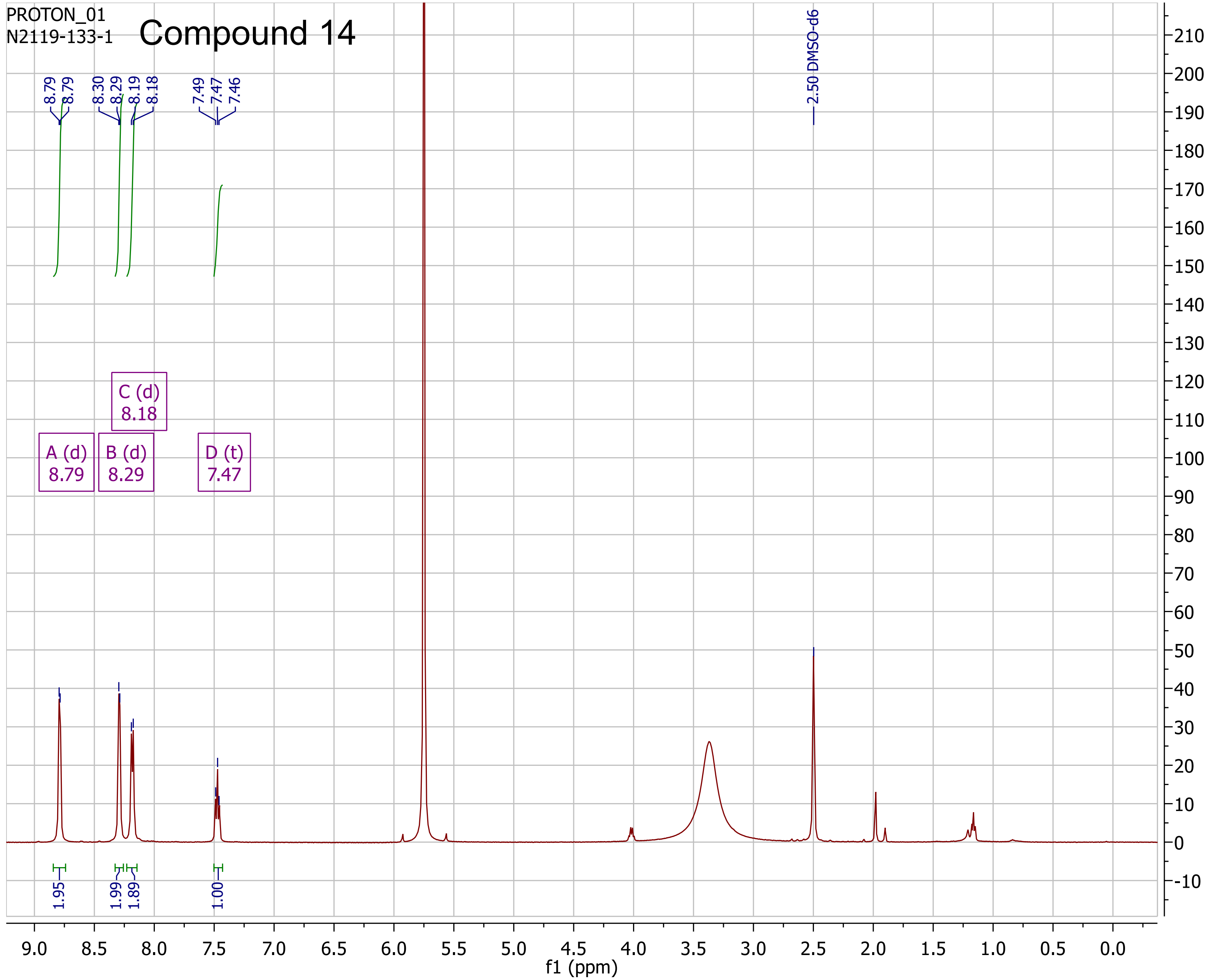

Sussex Drug Discovery Centre

	Parameter	Value
1	Data File Name	Y:/ walkup/ sew/ 20170921/ N2119-133-1_01/ PROTON_01.fid/ fid
2	Title	PROTON_01
3	Comment	N2119-133-1
4	Origin	Varian
5	Owner	
6	Site	
7	Spectrometer	vnmrs
8	Author	
9	Solvent	dmso
10	Temperature	30.0
11	Pulse Sequence	s2pul
12	Experiment	1D
13	Probe	P8898_walkup
14	Number of Scans	8
15	Receiver Gain	30
16	Relaxation Delay	1.0000
17	Pulse Width	4.3000
18	Presaturation Frequency	
19	Acquisition Time	2.0447
20	Acquisition Date	2017-09-21T12:23:43
21	Modification Date	2017-09-21T12:24:20
22	Class	
23	Spectrometer Frequency	499.91
24	Spectral Width	8012.8

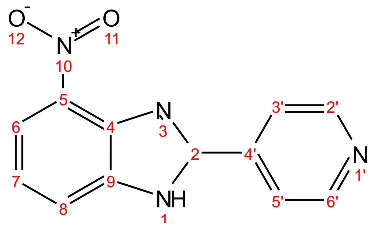

^{1}H NMR (500 MHz, DMSO-d_6) δ 8.79 (d, J = 4.1 Hz, 2H, H-2' and H-6'), 8.29 (d, J = 4.1 Hz, 2H, H-3' and H-5'), 8.18 (d, J = 7.7 Hz, 2H, H-6 and H-8), 7.47 (t, J = 7.0 Hz, 1H, H-7). Benzimidazole NH peak missing.

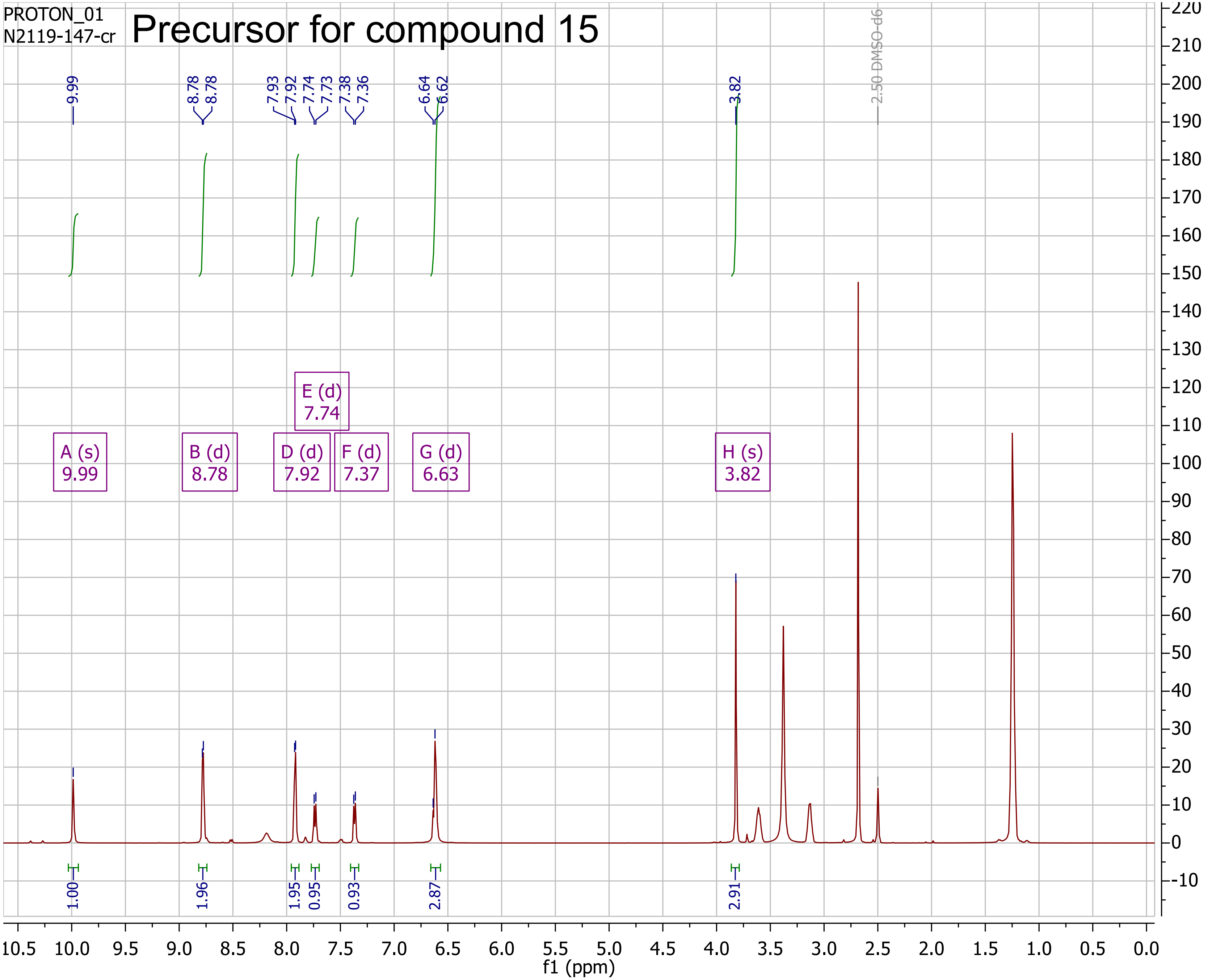

Sussex Drug Discovery Centre

	Parameter	Value
1	Data File Name	Z:/ walkup/ sew/ 20171023/ N2119-147-cr_01/ PROTON_01.fid/ fid
2	Title	PROTON_01
3	Comment	N2119-147-cr
4	Origin	Varian
5	Owner	
6	Site	
7	Spectrometer	vnmrs
8	Author	
9	Solvent	dmso
10	Temperature	30.0
11	Pulse Sequence	s2pul
12	Experiment	1D
13	Probe	P8898_walkup
14	Number of Scans	8
15	Receiver Gain	18
16	Relaxation Delay	1.0000
17	Pulse Width	4.3000
18	Presaturation Frequency	
19	Acquisition Time	2.0447
20	Acquisition Date	2017-10-23T12:13:18
21	Modification Date	2017-10-23T12:13:56
22	Class	
23	Spectrometer Frequency	499.91
24	Spectral Width	8012.8

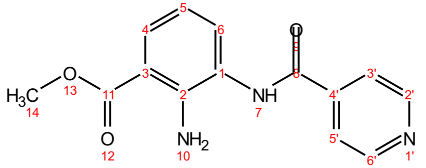

^{1}H NMR (500 MHz, DMSO-d_6) δ 9.99 (s, 1H, H-7), 8.78 (d, J = 4.4 Hz, 2H, H-2' and H-6'), 7.92 (d, J = 4.4 Hz, 2H, H-3' and H-5'), 7.74 (d, J = 8.0 Hz, 1H, H-4), 7.37 (d, J = 7.4 Hz, 1H, H-6), 6.63 (d, J = 8.6 Hz, 3H, H-10), 3.82 (s, 3H, H-14).

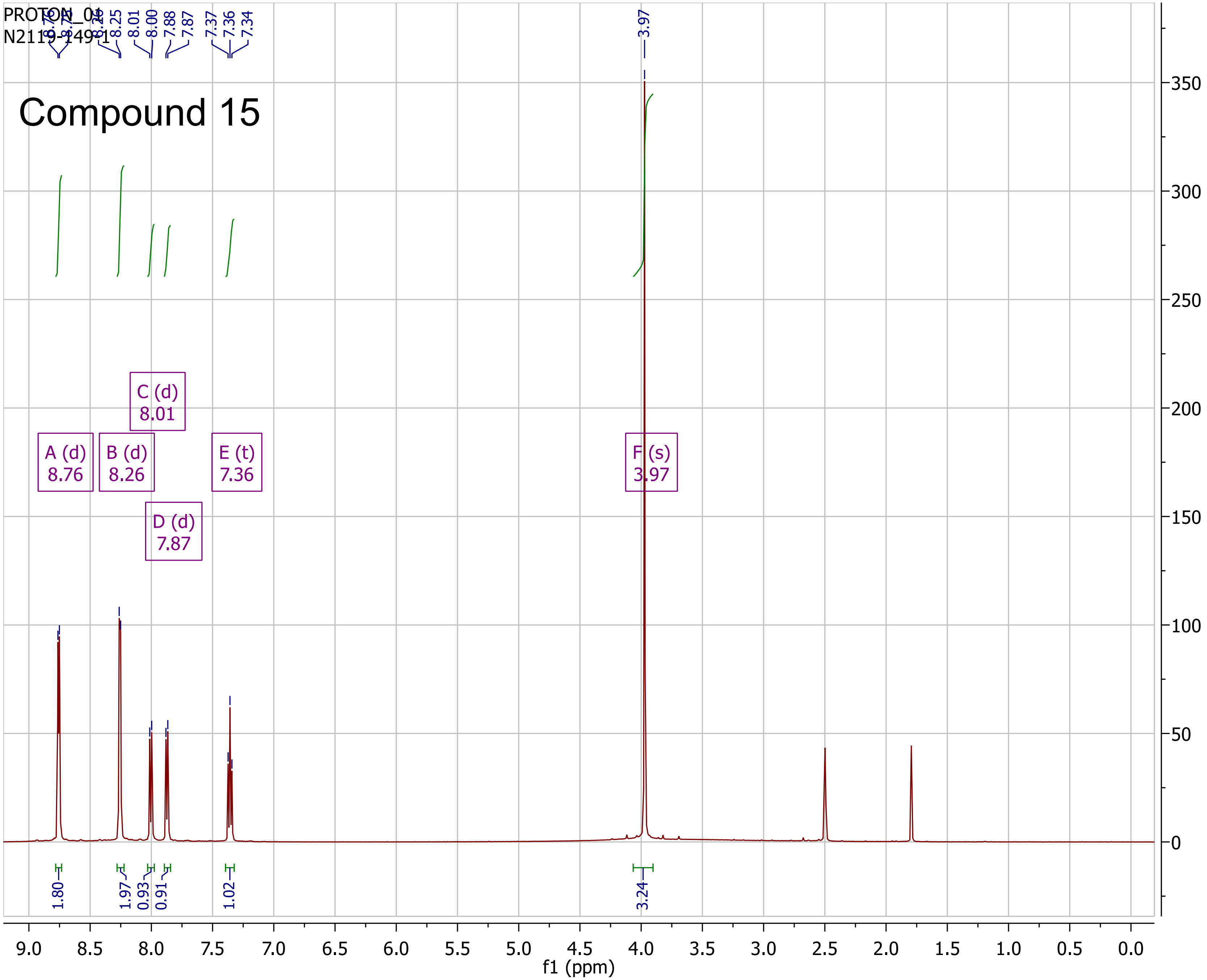

Parameter		Value
1	Data File Name	Z:/ walkup/ sew/ 20171026/ N2119-149-1_01/ PROTON_01.fid/ fid
2	Title	PROTON_01
3	Comment	N2119-149-1
4	Origin	Varian
5	Owner	
6	Site	
7	Spectrometer	vnmrs
8	Author	
9	Solvent	dmso
10	Temperature	30.0
11	Pulse Sequence	s2pul
12	Experiment	1D
13	Probe	P8898_walkup
14	Number of Scans	8
15	Receiver Gain	30
16	Relaxation Delay	1.0000
17	Pulse Width	4.3000
18	Presaturation Frequency	
19	Acquisition Time	2.0447
20	Acquisition Date	2017-10-26T09:45:00
21	Modification Date	2017-10-26T09:45:37
22	Class	
23	Spectrometer Frequency	499.91
24	Spectral Width	8012.8

^{1}H NMR (500 MHz, DMSO-d_6) δ 8.76 (d, J = 6.0 Hz, 2H, H-2' and H-6'), 8.26 (d, J = 6.0 Hz, 2H, H-3' and H-5'), 8.01 (d, J = 8.0 Hz, 1H, H-6), 7.87 (d, J = 7.6 Hz, 1H, H-8), 7.36 (t, J = 7.8 Hz, 1H, H-7), 3.97 (s, 3H, H-13).

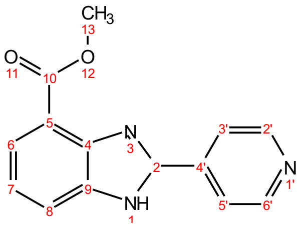

PROTON_01
N2119-104-1

Precursor for compound 16

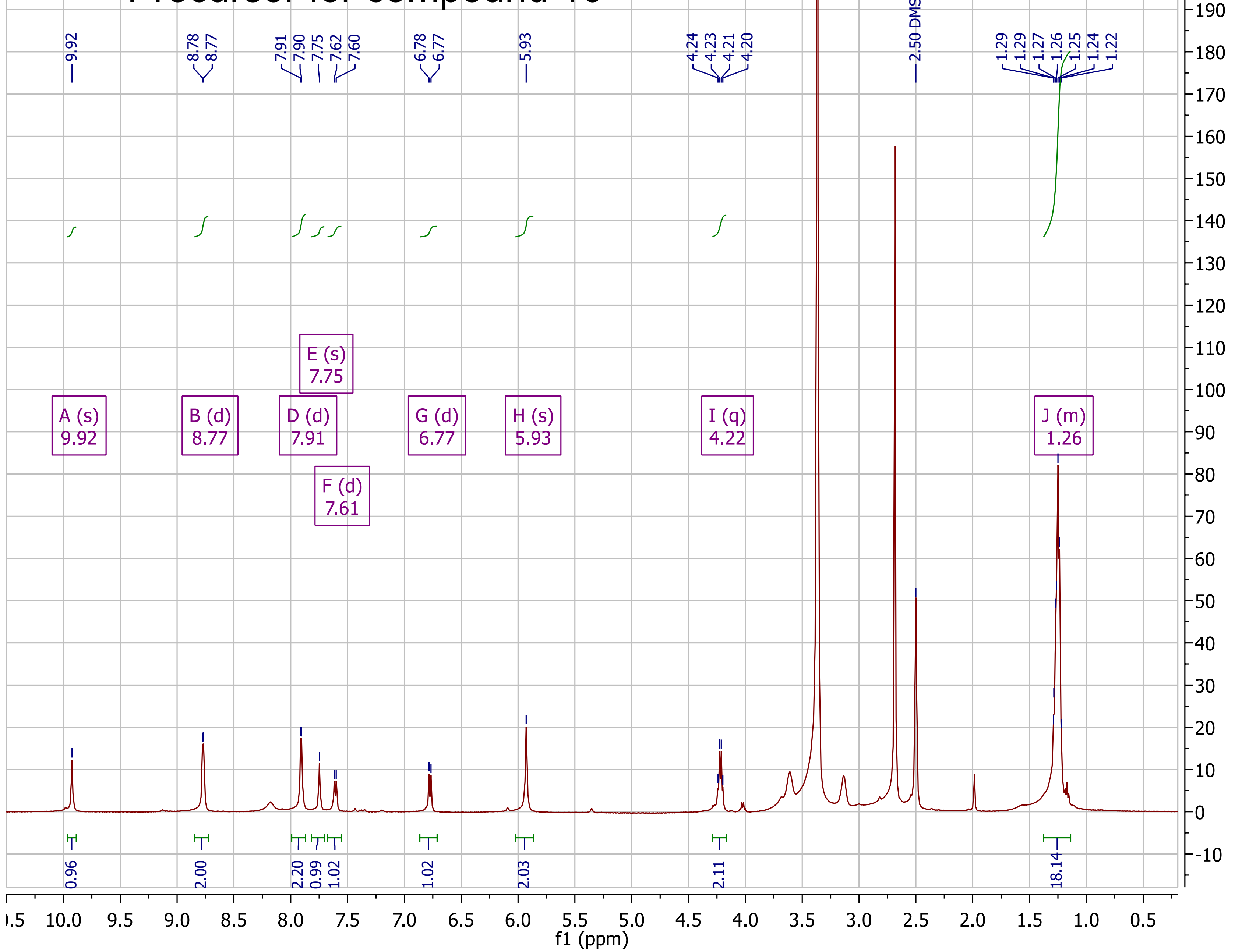

Sussex Drug Discovery Centre

	Parameter	Value
1	Data File Name	Y:/ walkup/ sew/ 20170817/ N2119-104-1_01/ PROTON_01.fid/ fid
2	Title	PROTON_01
3	Comment	N2119-104-1
4	Origin	Varian
5	Owner	
6	Site	
7	Spectrometer	vnmrs
8	Author	
9	Solvent	dmso
10	Temperature	30.0
11	Pulse Sequence	s2pul
12	Experiment	1D
13	Probe	P8898_walkup
14	Number of Scans	8
15	Receiver Gain	30
16	Relaxation Delay	1.0000
17	Pulse Width	4.3000
18	Presaturation Frequency	
19	Acquisition Time	2.0447
20	Acquisition Date	2017-08-17T17:38:36
21	Modification Date	2017-08-17T17:39:31
22	Class	
23	Spectrometer Frequency	499.91
24	Spectral Width	8012.8

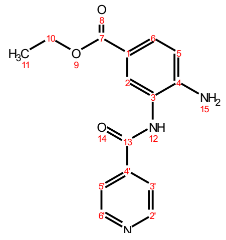

^{1}H NMR (500 MHz, DMSO-d_6) δ 9.92 (s, 1H, H-12), 8.77 (d, J = 4.2 Hz, 2H, H-2' and H-6'), 7.91 (d, J = 4.4 Hz, 2H, H-3' and H-5'), 7.75 (s, 1H, H-2), 7.61 (d, J = 8.5 Hz, 1H, H-6), 6.77 (d, J = 8.5 Hz, 2H, H-5), 5.93 (s, 2H, H-15), 4.22 (q, J = 7.1 Hz, 2H, H-10), 1.42 – 0.97 (m, 3H, H-11).

Sussex Drug Discovery Centre

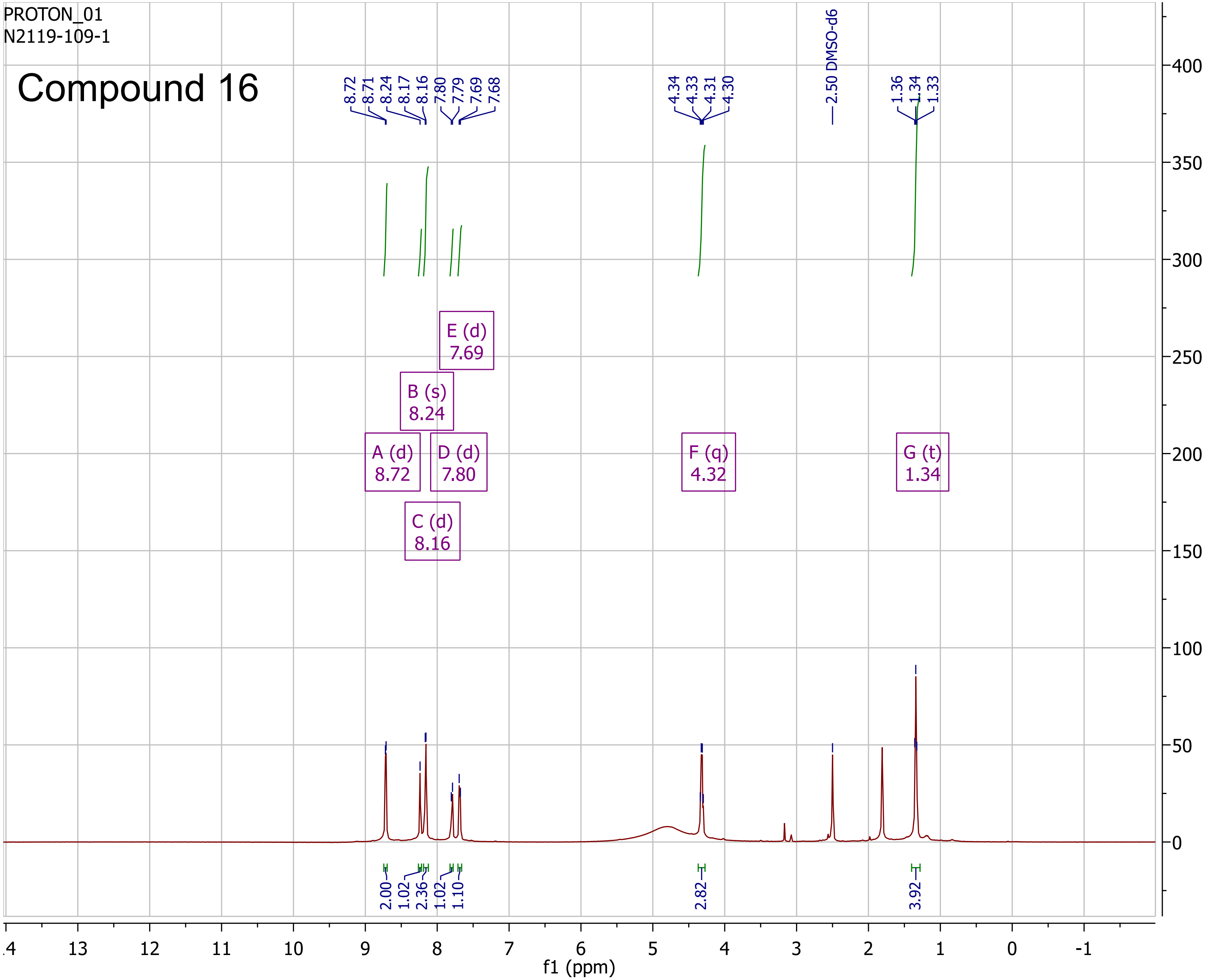

	Parameter	Value
1	Data File Name	Y:/ walkup/ sew/ 20170825/ N2119-109-1_01/ PROTON_01.fid/ fid
2	Title	PROTON_01
3	Comment	N2119-109-1
4	Origin	Varian
5	Owner	
6	Site	
7	Spectrometer	vnmrs
8	Author	
9	Solvent	dmso
10	Temperature	30.0
11	Pulse Sequence	s2pul
12	Experiment	1D
13	Probe	P8898_walkup
14	Number of Scans	8
15	Receiver Gain	30
16	Relaxation Delay	1.0000
17	Pulse Width	4.3000
18	Presaturation Frequency	
19	Acquisition Time	2.0447
20	Acquisition Date	2017-08-25T16:38:43
21	Modification Date	2017-08-25T16:39:37
22	Class	

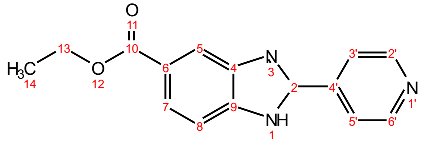

^{1}H NMR (500 MHz, DMSO-d_6) δ 8.72 (d, J = 4.7 Hz, 2H (H-2' and H-6'), 8.24 (s, 1H, H-5), 8.16 (d, J = 4.9 Hz, 2H, H-3' and H-5'), 7.80 (d, J = 8.4 Hz, 1H, H-7), 7.69 (d, J = 8.4 Hz, 1H, H-8), 4.32 (q, J = 7.0 Hz, 2H, H-13), 1.34 (t, J = 7.1 Hz, 3H, H-14).

PROTON_01
N2119-124-cr

Precursor for compound 17

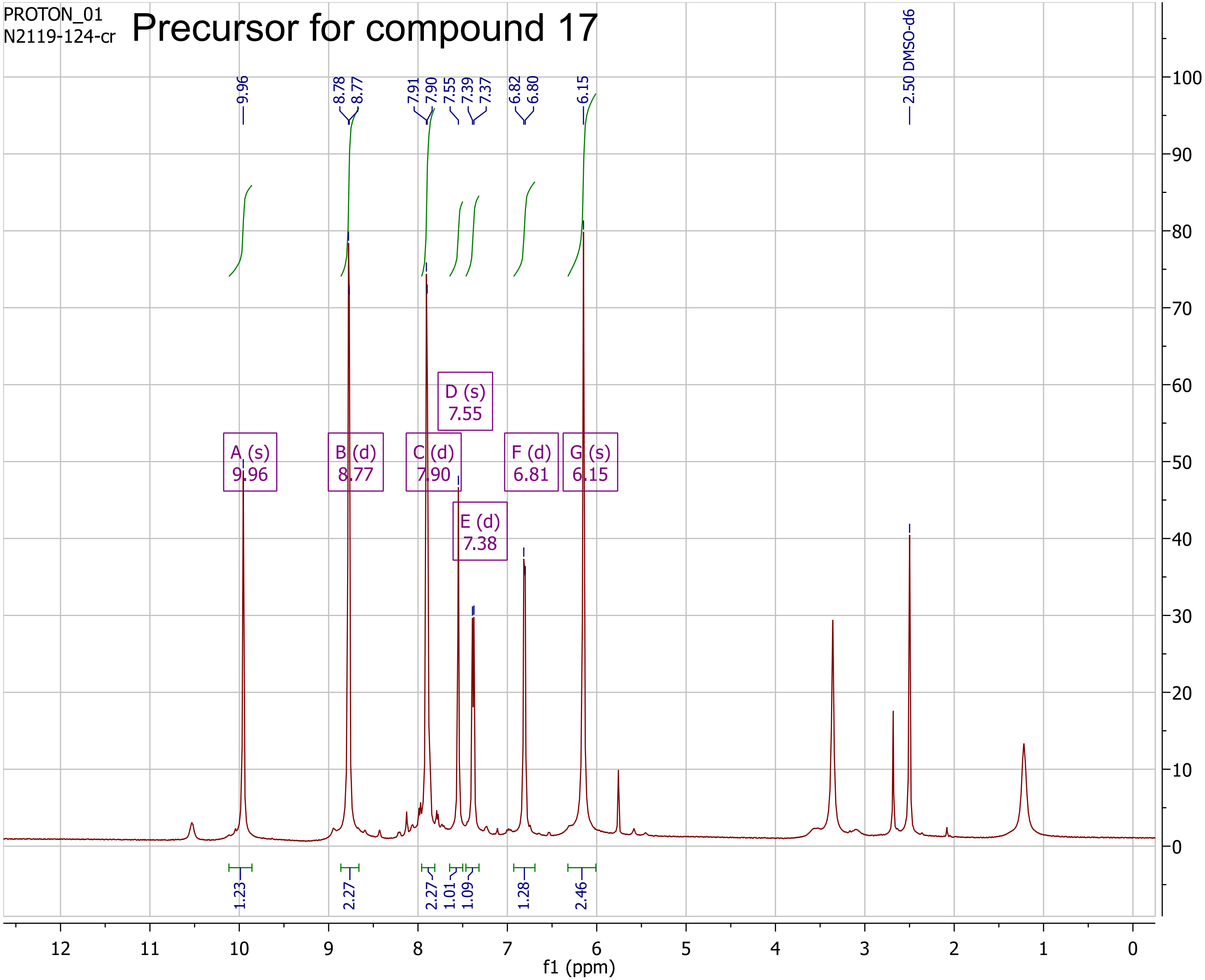

Sussex Drug Discovery Centre

	Parameter	Value
1	Data File Name	Y:/ walkup/ sew/ 20170912/ N2119-124-cr_01/ PROTON_01.fid/ fid
2	Title	PROTON_01
3	Comment	N2119-124-cr
4	Origin	Varian
5	Owner	
6	Site	
7	Spectrometer	vnmrs
8	Author	
9	Solvent	dmso
10	Temperature	30.0
11	Pulse Sequence	s2pul
12	Experiment	1D
13	Probe	P8898_walkup
14	Number of Scans	8
15	Receiver Gain	30
16	Relaxation Delay	1.0000
17	Pulse Width	4.3000
18	Presaturation Frequency	
19	Acquisition Time	2.0447
20	Acquisition Date	2017-09-12T11:50:46
21	Modification Date	2017-09-12T11:51:26
22	Class	
[illegible]	[illegible]	[illegible]

^{1}H NMR (500 MHz, DMSO-d_6) δ 9.96 (s, 1H, H-9), 8.77 (d, J = 4.2 Hz, 2H, H-2' and H-6'), 7.90 (d, J = 4.4 Hz, 2H, H-3' and H-5'), 7.55 (s, 1H, H-2), 7.38 (d, J = 8.1 Hz, 1H, H-6), 6.81 (d, J = 8.4 Hz, 1H, H-5), 6.15 (s, 2H, H-12).

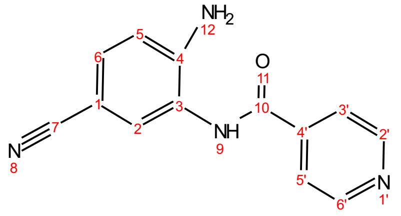

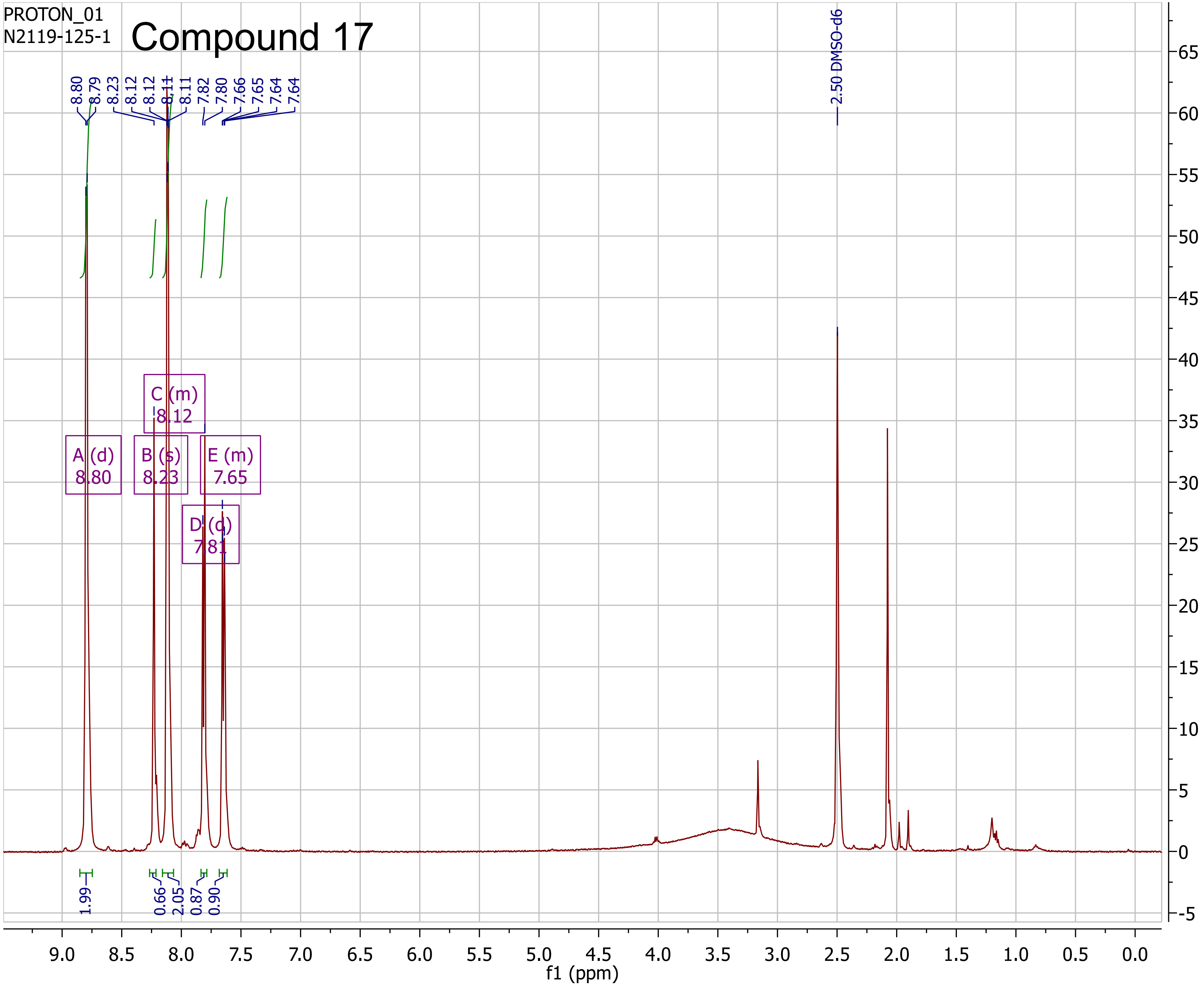

Sussex Drug Discovery Centre

	Parameter	Value
1	Data File Name	Y:/ walkup/ sew/ 20170915/ n2119-125-1_01/ PROTON_01.fid/ fid
2	Title	PROTON_01
3	Comment	N2119-125-1
4	Origin	Varian
5	Owner	
6	Site	
7	Spectrometer	vnmrs
8	Author	
9	Solvent	dmso
10	Temperature	30.0
11	Pulse Sequence	s2pul
12	Experiment	1D
13	Probe	P8898_walkup
14	Number of Scans	8
15	Receiver Gain	30
16	Relaxation Delay	1.0000
17	Pulse Width	4.3000
18	Presaturation Frequency	
19	Acquisition Time	2.0447
20	Acquisition Date	2017-09-15T23:46:09
21	Modification Date	2017-09-15T23:46:36
22	Class	

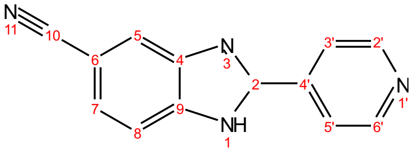

^{1}H NMR (500 MHz, DMSO-d_6) δ 8.80 (d, J = 5.7 Hz, 2H, H-2' and H-6'), 8.23 (s, 1H, H-5), 8.15 – 8.05 (m, 2H, H-3' and H-5'), 7.81 (d, J = 8.4 Hz, 1H, H-7), 7.66 – 7.62 (m, 1H, H-8).

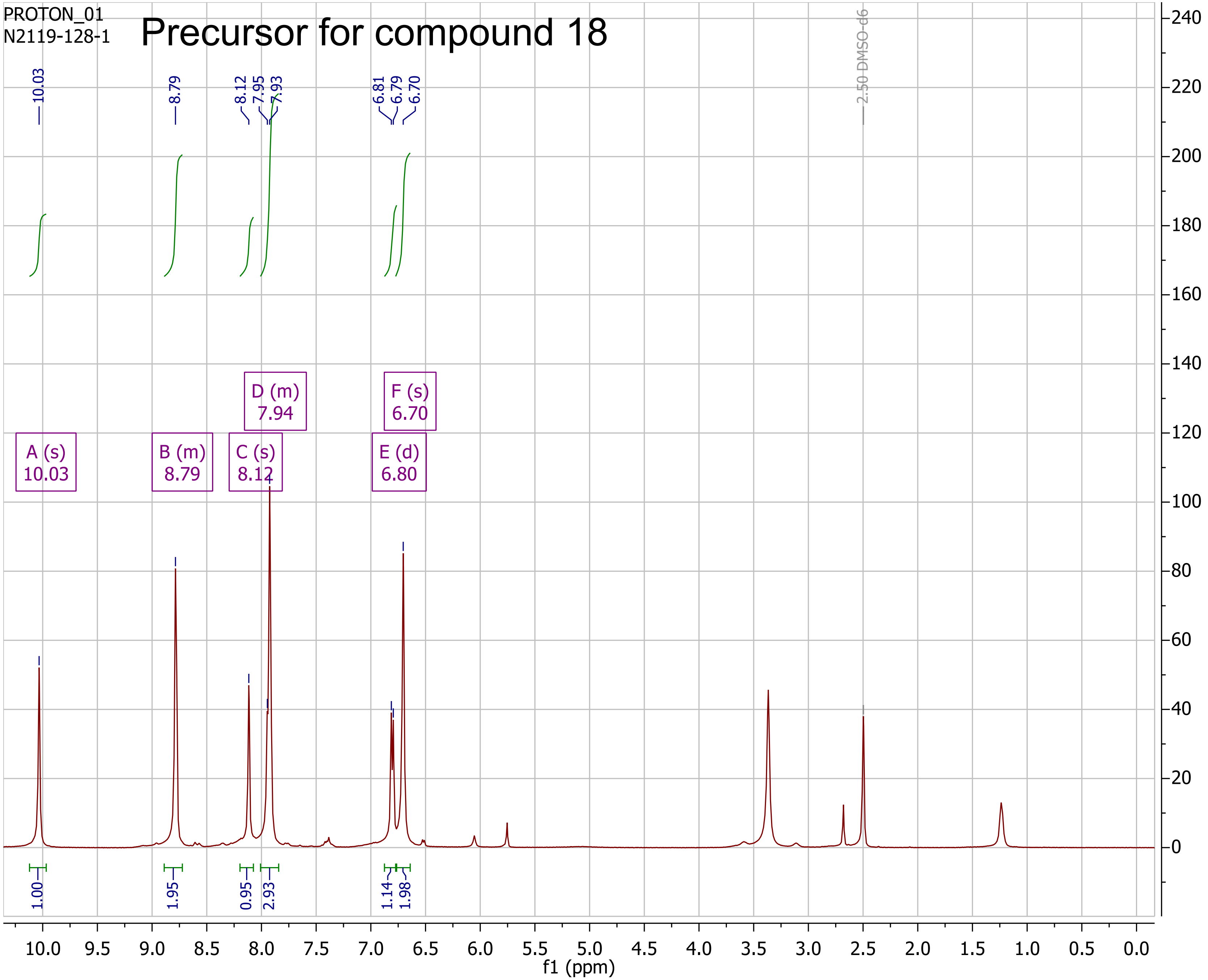

Sussex Drug Discovery Centre

	Parameter	Value
1	Data File Name	X:/ walkup/ sew/ 20170919/ N2119-128-1_01/ PROTON_01.fid/ fid
2	Title	PROTON_01
3	Comment	N2119-128-1
4	Origin	Varian
5	Spectrometer	vnmrs
6	Solvent	dmso
7	Temperature	30.0
8	Pulse Sequence	s2pul
9	Experiment	1D
10	Probe	P8898_walkup
11	Number of Scans	8
12	Receiver Gain	30
13	Relaxation Delay	1.0000
14	Pulse Width	4.3000
15	Acquisition Time	2.0447
16	Acquisition Date	2017-09-19T15:05:43
17	Modification Date	2017-09-19T15:06:19
18	Spectrometer Frequency	499.91
19	Spectral Width	8012.8
20	Lowest Frequency	-996.5
21	Nucleus	1H
22	Acquired Size	16384
23	Spectral Size	65536

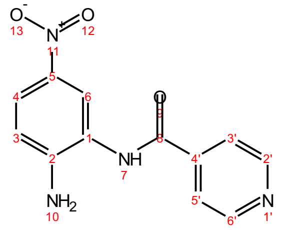

^{1}H NMR (500 MHz, DMSO-d_6) δ 10.03 (s, 1H, H-7), 8.88 – 8.70 (m, 2H, H-2' and H-6'), 8.12 (s, 1H, H-6), 8.00 – 7.86 (m, 3H, H-4, H-3' and H-5'), 6.80 (d, *J* = 8.9 Hz, 1H, H-3), 6.70 (s, 2H, H-10).

PROTON_01
N2119-134-1

Compound 18

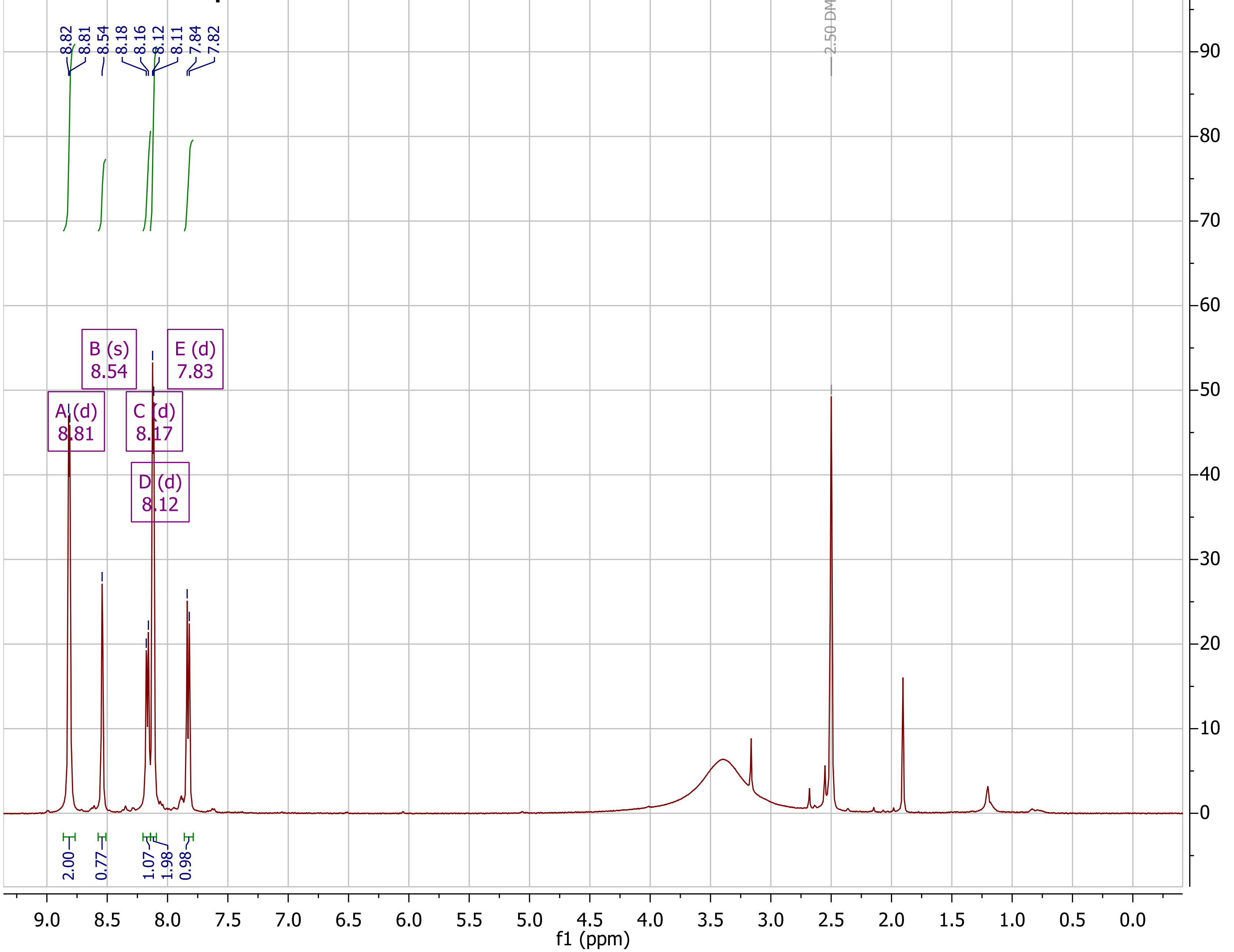

Sussex Drug Discovery Centre

	Parameter	Value
1	Data File Name	Y:/ walkup/ sew/ 20170921/ N2119-134-1_01/ PROTON_01.fid/ fid
2	Title	PROTON_01
3	Comment	N2119-134-1
4	Origin	Varian
5	Owner	
6	Site	
7	Spectrometer	vnmrs
8	Author	
9	Solvent	dmso
10	Temperature	30.0
11	Pulse Sequence	s2pul
12	Experiment	1D
13	Probe	P8898_walkup
14	Number of Scans	8
15	Receiver Gain	30
16	Relaxation Delay	1.0000
17	Pulse Width	4.3000
18	Presaturation Frequency	
19	Acquisition Time	2.0447
20	Acquisition Date	2017-09-21T12:28:26
21	Modification Date	2017-09-21T12:28:55
22	Class	
23	[illegible]	[illegible]

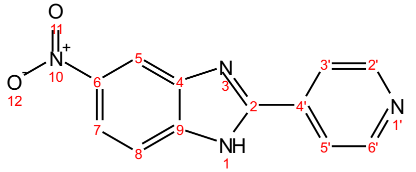

^{1}H NMR (500 MHz, DMSO-d_6) δ 8.81 (d, J = 4.9 Hz, 2H, H-2' and H-6'), 8.54 (s, 1H, H-5), 8.17 (d, J = 8.9 Hz, 1H, H-7), 8.12 (d, J = 4.8 Hz, 2H, H-3' and H-5'), 7.83 (d, J = 8.8 Hz, 1H, H-8).

Sussex Drug Discovery Centre

Precursor for compound 19

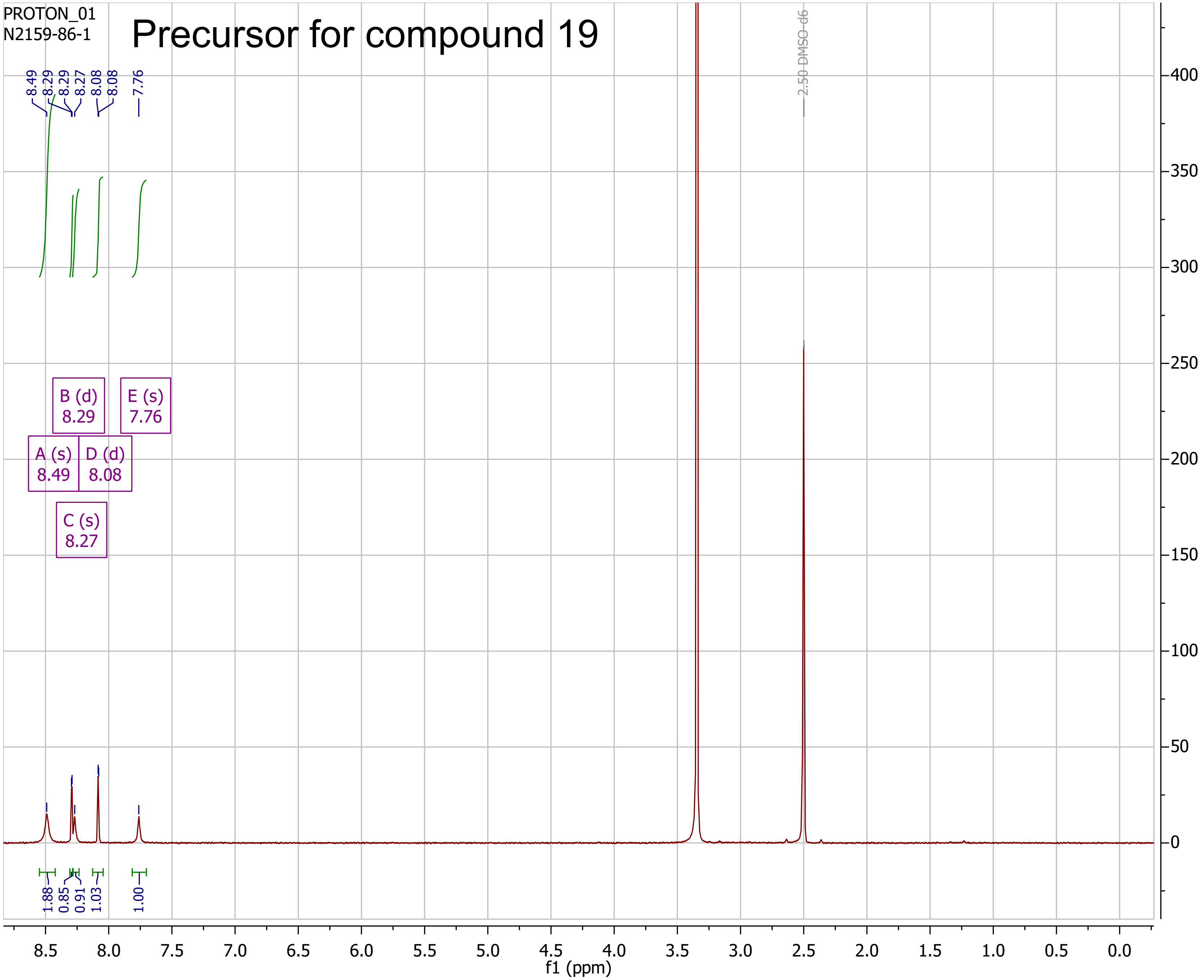

	Parameter	Value
1	Data File Name	X:/ walkup/ sew/ 20180905/ N2159-86-1_01/ PROTON_01.fid/ fid
2	Title	PROTON_01
3	Comment	N2159-86-1
4	Origin	Varian
5	Owner	
6	Site	
7	Instrument	vnmrs
8	Author	
9	Solvent	dmso
10	Temperature	25.0
11	Pulse Sequence	s2pul
12	Experiment	1D
13	Probe	P965_loanprobe
14	Number of Scans	8
15	Receiver Gain	48
16	Relaxation Delay	1.0000
17	Pulse Width	5.7812
18	Presaturation Frequency	
19	Acquisition Time	2.0447
20	Acquisition Date	2018-09-05T16:34:36
21	Modification Date	2018-09-05T16:35:12
22	Class	
23	[illegible]	[illegible]

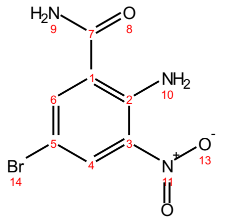

^{1}H NMR (500 MHz, DMSO-d_6) δ 8.49 (s, 2H, H-9), 8.29 (d, J = 2.3 Hz, 1H, H-4), 8.27 (s, 1H, H-10), 8.08 (d, J = 2.3 Hz, 1H, H-6), 7.76 (s, 1H, H-10).

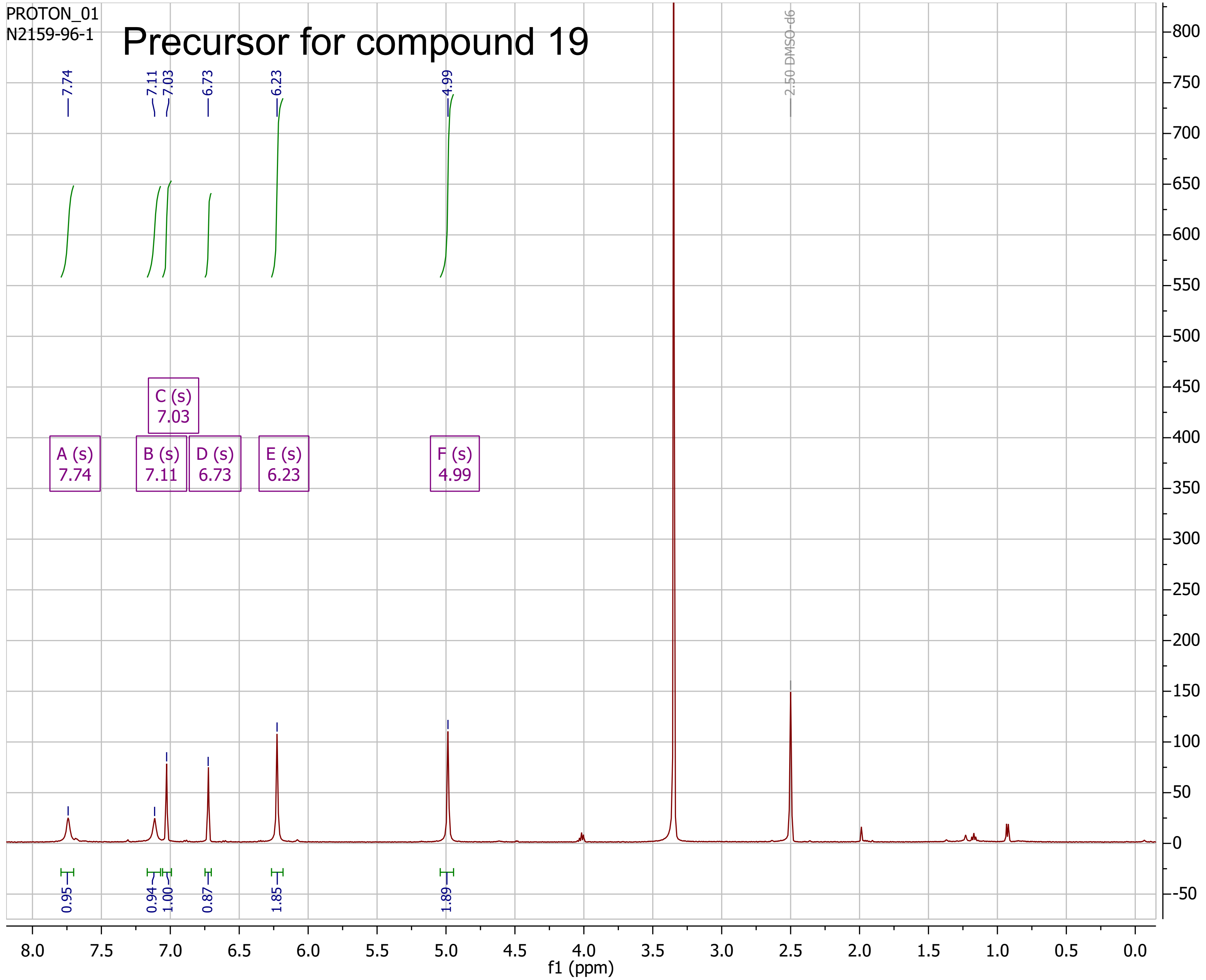

Sussex Drug Discovery Centre

	Parameter	Value
1	Data File Name	E:/ N2159-96-1_01/ PROTON_01.fid/ fid
2	Title	PROTON_01
3	Comment	N2159-96-1
4	Origin	Varian
5	Owner	
6	Site	
7	Instrument	vnmrs
8	Author	
9	Solvent	dmso
10	Temperature	25.0
11	Pulse Sequence	s2pul
12	Experiment	1D
13	Probe	P965_loanprobe
14	Number of Scans	8
15	Receiver Gain	46
16	Relaxation Delay	1.0000
17	Pulse Width	5.7812
18	Presaturation Frequency	
19	Acquisition Time	2.0447
20	Acquisition Date	2018-09-21T10:54:01
21	Modification Date	2018-09-21T09:54:36
22	Class	
23	Spectrometer Frequency	499.91
24	Spectral Width	8012.8
25	Lowest [illegible]	-998.5

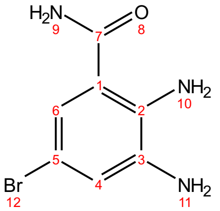

^{1}H NMR (500 MHz, DMSO-d_6) δ 7.74 (s, 1H, H-10), 7.11 (s, 1H, H-10), 7.03 (s, 1H, H-6), 6.73 (s, 1H, H-4), 6.23 (s, 2H, H-9), 4.99 (s, 2H, H-11).

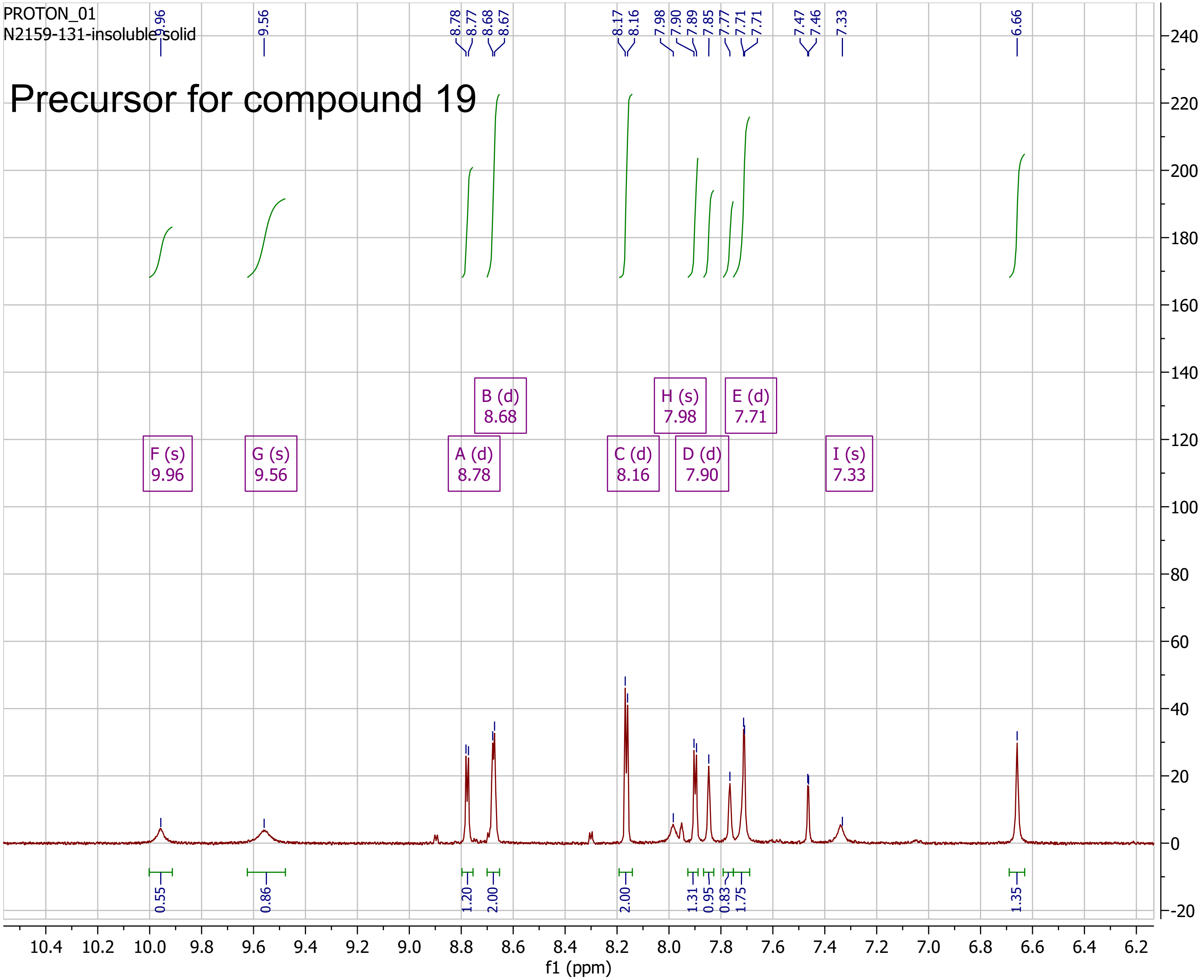

Sussex Drug Discovery Centre

	Parameter	Value
1	Data File Name	E:/ N2159-131-insoluble_solid_01/ PROTON_01.fid/ fid
2	Title	PROTON_01
3	Comment	N2159-131-insoluble solid
4	Origin	Varian
5	Owner	
6	Site	
7	Instrument	vnmrs
8	Author	
9	Solvent	dmso
10	Temperature	25.0
11	Pulse Sequence	s2pul
12	Experiment	1D
13	Probe	P8891_walkup
14	Number of Scans	8
15	Receiver Gain	52
16	Relaxation Delay	1.0000
17	Pulse Width	6.4688
18	Presaturation Frequency	
19	Acquisition Time	1.7039
20	Acquisition Date	2018-11-21T17:08:17
21	Modification Date	2018-11-21T17:08:52
22	Class	
23	Spectrometer Frequency	599.68
24	Spectral Width	9615.4

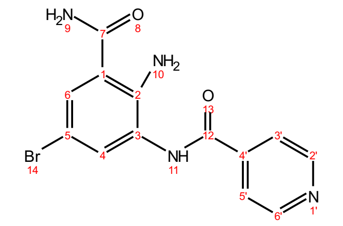

^{1}H NMR (600 MHz, DMSO-d_6) δ 9.96 (s, 1H, NH), 9.56 (s, 1H, NH), 8.78 (d, J = 5.8 Hz, 1H, H-6), 8.68 (d, J = 4.3 Hz, 2H, H-2' and H-6'), 8.16 (d, J = 5.4 Hz, 2H, H-3' and H-5'), 7.98 (s, 1H, NH), 7.90 (d, J = 5.8 Hz, 1H, H-4), 7.71 (d, J = 2.1 Hz, 2H, NH2), 7.33 (s, 1H, NH). Multiple peaks due to crude mixture.

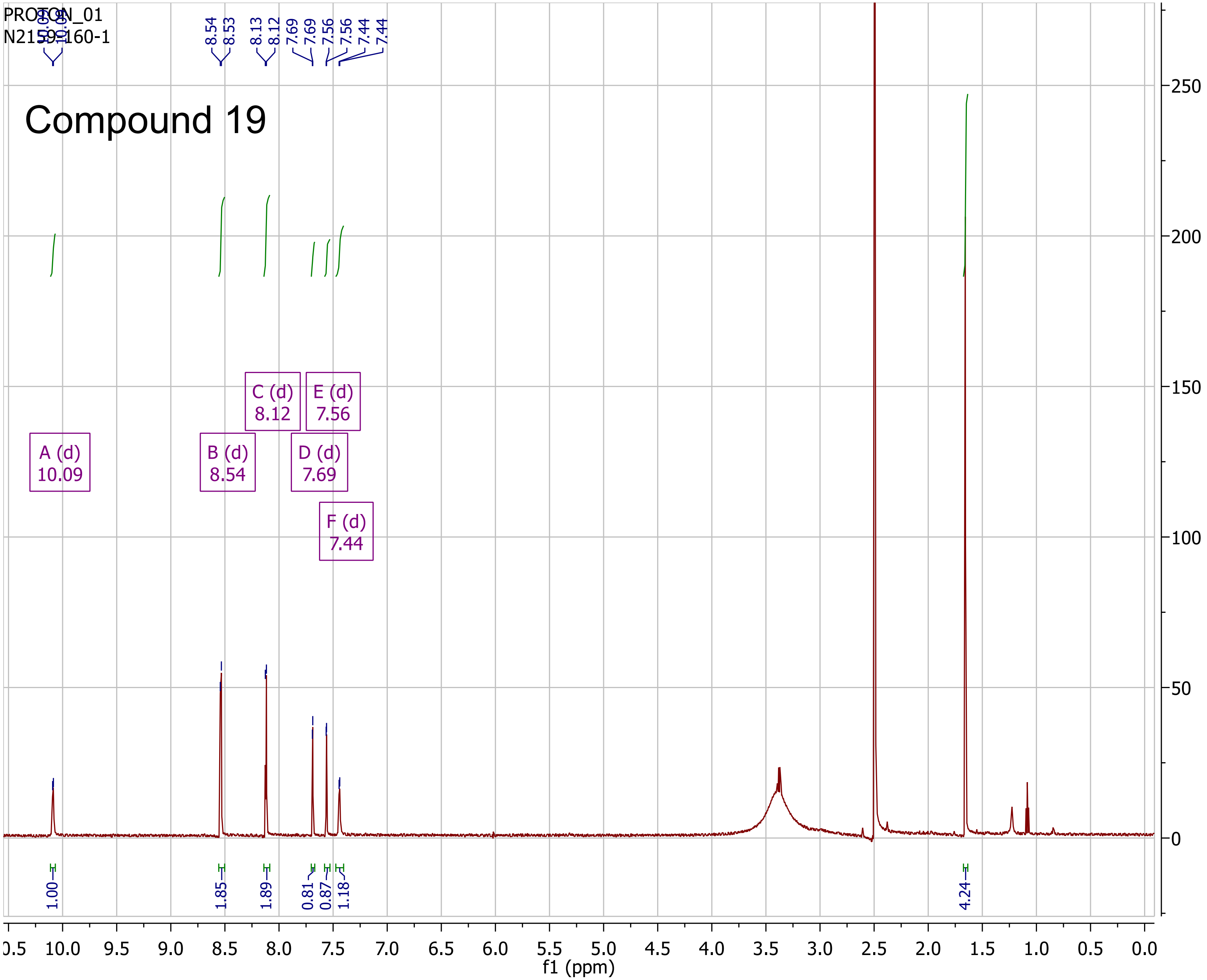

Sussex Drug Discovery Centre

	Parameter	
1	Data File Name	
2	Title	PROTON_01
3	Comment	N2159-160-1
4	Origin	Varian
5	Owner	
6	Site	
7	Instrument	vnmrs
8	Author	
9	Solvent	dmso
10	Temperature	25.0
11	Pulse Sequence	s2pul
12	Experiment	1D
13	Probe	P8891_walkup
14	Number of Scans	8
15	Receiver Gain	52
16	Relaxation Delay	1.0000
17	Pulse Width	6.4688
18	Presaturation Frequency	
19	Acquisition Time	1.7039
20	Acquisition Date	2019-03-05T14:53:24
21	Modification Date	2019-03-05T14:53:58
22	Class	
23	Spectrometer Frequency	599.68

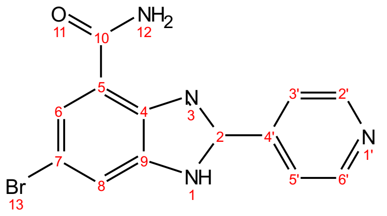

^{1}H NMR (600 MHz, DMSO-d_6) δ 10.09 (d, J = 4.2 Hz, 1H, H-12), 8.54 (d, J = 6.2 Hz, 2H, H-2' and H-6'), 8.12 (d, J = 6.0 Hz, 2H, H-3' and H-5'), 7.69 (d, J = 2.0 Hz, 1H, H-6), 7.56 (d, J = 2.0 Hz, 1H, H-8), 7.44 (d, J = 3.6 Hz, 1H, H-12).

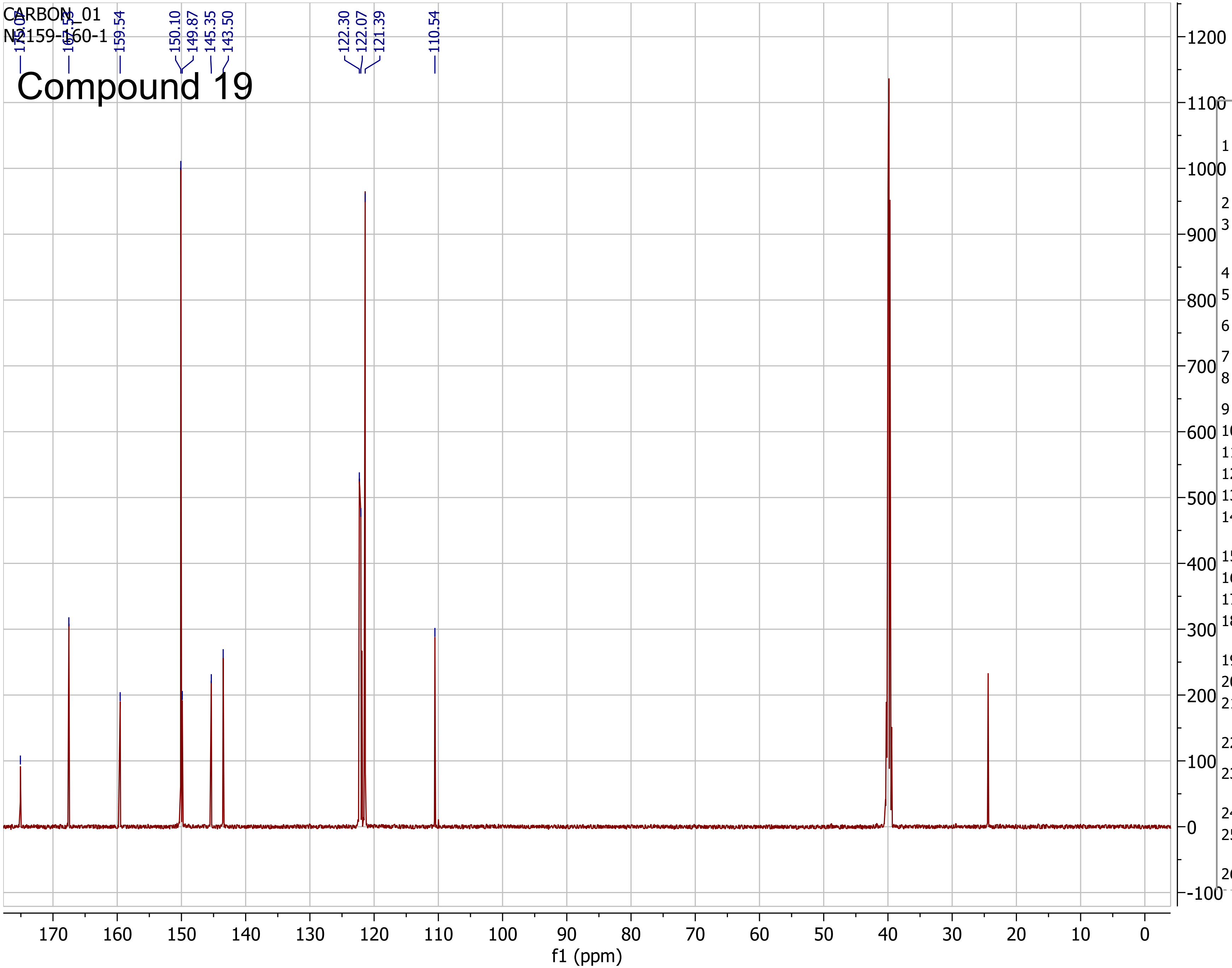

Sussex Drug Discovery Centre

	Parameter	Value
1	Data File Name	D:/ NMR/ N2159/ N2159-160-1_01/ CARBON_01.fid/ fid
2	Title	CARBON_01
3	Comment	N2159-160-1
4	Origin	Varian
5	Owner	
6	Site	
7	Spectrometer	vnmrs
8	Author	
9	Solvent	dmso
10	Temperature	25.0
11	Pulse Sequence	s2pul
12	Experiment	1D
13	Probe	P8891_walkup
14	Number of Scans	10000
15	Receiver Gain	30
16	Relaxation Delay	1.0000
17	Pulse Width	6.2500
18	Presaturation Frequency	
19	Acquisition Time	0.8651
20	Acquisition Date	2019-09-15T03:49:22
21	Modification Date	2019-09-15T08:00:18
22	Class	
23	Spectrometer Frequency	150.81
24	Spectral Width	37878.8
25	Lowest Frequency	-2400.7
26	Nucleus	13C
27	[illegible]	[illegible]

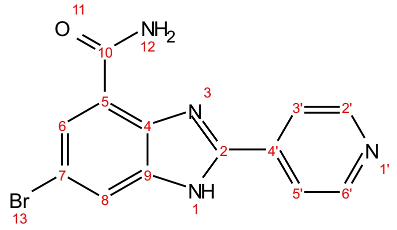

^{13}C NMR (151 MHz, DMSO-d6) δ 175.1 (C-10), 167.5 (C-7), 159.5 (C-2), 150.1 (C-2' and C-6'), 149.9 (C-5), 145.4 (C-4), 143.5 (C-4'), 122.3 (C-6), 122.1 (C-8), 121.4 (C-3' and C-5'), 110.5 (C-9).
